# Enhancing link prediction in biomedical knowledge graphs with BioPathNet

**DOI:** 10.1038/s41551-025-01598-z

**Published:** 2026-01-20

**Authors:** Emy Yue Hu, Svitlana Oleshko, Samuele Firmani, Hui Cheng, Zhaocheng Zhu, Maria Ulmer, Matthias Arnold, Maria Colomé-Tatché, Jian Tang, Sophie Xhonneux, Annalisa Marsico

**Affiliations:** 1 Computational Health Center, Helmholtz Center Munich, Oberschleißheim, Germany; 2 School of Life Sciences, Technical University of Munich, Freising, Germany; 3 School of Computation, Information and Technology, Technical University of Munich, Munich, Germany; 4 Mila – Québec AI Institute, Montréal, Quebec Canada; 5 Université de Montréal, Montréal, Quebec Canada; 6 Department of Psychiatry and Behavioural Sciences, Duke University, Durham, NC USA; 7 Faculty of Biology, Ludwig-Maximilian University of Munich, Planegg-Martinsried, Germany; 8 CIFAR AI Chair, Toronto, Ontario Canada; 9 HEC Montréal, Montréal, Quebec Canada

**Keywords:** Computational models, Predictive medicine, Machine learning

## Abstract

Understanding complex interactions in biomedical networks is crucial for advancements in biomedicine, but traditional link prediction (LP) methods are limited in capturing this complexity. We present BioPathNet, a graph neural network framework based on the neural Bellman–Ford network (NBFNet), addressing limitations of traditional representation-based learning methods through path-based reasoning for LP in biomedical knowledge graphs. Unlike node-embedding frameworks, BioPathNet learns representations between node pairs by considering all relations along paths, enhancing prediction accuracy and interpretability, and allowing visualization of influential paths and biological validation. BioPathNet leverages a background regulatory graph for enhanced message passing and uses stringent negative sampling to improve precision and scalability. BioPathNet outperforms or matches existing methods across diverse tasks including gene function annotation, drug–disease indication, synthetic lethality and lncRNA–target interaction prediction. Our study identifies promising additional drug indications for diseases such as acute lymphoblastic leukaemia and Alzheimer’s disease, validated by medical experts and clinical trials. In addition, we prioritize putative synthetic lethal gene pairs and regulatory lncRNA–target interactions. BioPathNet’s interpretability will enable researchers to trace prediction paths and gain molecular insights.

## Abstract (web summary)

BioPathNet predicts drug–disease indications, synthetic lethality and lncRNA–mRNA interactions using a deep learning framework.

## Main

Biological entities interact in complex ways, crucial for sustaining life^1^. Understanding these interactions is central to systems biology, with network analysis playing a key role^2^. Biological networks are represented as graphs, where nodes can represent genes, proteins, diseases and more, and edges denote associations between them. Edges in a biological graph can signify co-regulation between genes or causal relationship (regulatory network)^3,4^, physical interactions (in protein–protein interaction networks (PPI)^5,6^), as well as disease–gene associations (such as in disease–gene networks^7,8^), among many.

Despite increasing high-throughput experiments, our grasp of biological networks is incomplete, leaving many interactions undiscovered. Due to the expense and time involved in wet lab experiments, computational methods such as link prediction (LP) are very important for inferring potential associations within these networks based on the underlying topology^9^. LP is applied across network biology for diverse tasks ranging from predicting protein interactions to inferring gene regulatory networks^10^. By revealing hidden connections, LP facilitates the discovery of biomarkers, drug targets and insights into biological interactions^11,12^. To predict potential relationships between unconnected nodes, one prevalent class of methods uses similarity metrics from traditional graph analysis, such as Personalized PageRank, Jaccard or Katz index^13,14^. These metrics have been used for predicting disease–gene associations^15^, including non-coding RNA–disease relationships and drug–disease associations^16^.

While traditional graph metrics have been successful in biological LP, representation-based learning offers greater expressiveness for capturing the nuances and complexity of nodes in a graph. Nodes are mapped to low-dimensional vector representations called embeddings using shallow and deep nonlinear transformations. Optimized embeddings position nodes with similar network neighbourhoods closely in the embedding space so that links between nodes can be predicted on the basis of their similarity in this space^17^. Methods include matrix factorization (for example, Mashup^18^) and random walk approaches (for example, DeepWalk^19^, node2vec^20^, struc2vec^21^).

Network embedding techniques have been successful in drug repurposing, adverse drug reaction prediction, gene function prediction and protein–protein interaction network completion^22–25^. For instance, ref. ^26^ introduced the multiscale interactome, integrating disease-associated proteins, drug targets and biological functions using biased random walks for node embeddings. GeneWalk predicts gene functions via network representation learning with random walks^27^. Reference ^28^ constructed a multimodal network of genes and polygenic risk scores for diseases to uncover associations between COVID-19 genes, co-morbidities and genetic predispositions^28–30^.

Recent efforts use heterogeneous multirelational networks, more specifically knowledge graphs (KGs), to better represent biological complexities by modelling facts as subject–predicate–object (SPO) triplets. KG research is increasingly applied to tasks such as question answering and information retrieval, with a key challenge being LP by estimating missing triplet components. Knowledge Graph Embedding (KGE) effectively learns low-rank representations of entities and relations, preserving graph structure and encoding relation semantics by optimizing a training loss that maximizes scores for positive triplets while minimizing those for corrupted triplets^31^. KGE methods, such as TransE^31^, DistMult^32^, ComplEx^33^ and RotatE^34^, have shown notable performance in several benchmark tests, but they are often limited to one-hop relations. In large biomedical KGs, relationships between entities are intricate and may involve multihop paths. Methods such as SEAL^35^ and Grail^36^ address this by embedding subgraph structures around links, but their scalability is limited due to the need to generate subgraphs for each link, creating bottlenecks in large-scale predictions.

Unlike KGEs, deep learning methods—particularly graph convolutional networks (GCNs)^37^, a class of message passing neural networks (MPNNs)—have substantially advanced KGEs^38,39^. These models have greatly improved performance in biological tasks such as drug–target interaction prediction^40,41^, polypharmacy side effect modelling^25^, tissue-specific protein function prediction^23,24^ and drug–disease indication discovery^42^. In addition, they have been applied to multirelational and heterogeneous graphs to capture diverse entity association semantics.

For example, the relational graph convolutional network (R-GCN) for multirelational KGs^38^ learns node embeddings by aggregating transformed feature vectors of neighbouring nodes via a normalized sum and uses the DistMult factorization model for LP. While R-GCN incorporates relation-specific transformations based on edge type and direction, making it well suited for multirelational data in KGs, it does not explicitly learn a representation for relations. More expressive methods include relation-aware graph attention network (RAGAT), a GAT-based model that utilizes relation-specific network parameters to learn embeddings for both entities and relations, combining them into joint embeddings for each relation type^43^. Similarly, the heterogeneous graph transformer (HGT) models graph heterogeneity by employing node- and edge-type-dependent parameters, including a node- and edge-specific attention mechanism. This approach preserves distinct representations for nodes and edges while leveraging its architecture to incorporate information from higher-order neighbourhoods to be used in downstream tasks^44^.

With the rapid accumulation of biomedical data, understanding disease biology and molecular factors’ roles in phenotypic outcomes is crucial for personalized diagnostics and treatments. KGs have also become the dominant knowledge representation in biomedicine, leveraging databases such as UniProt^45^, Gene Ontology^46,47^ and DrugBank^48^. LP tasks in biomedical KGs, such as the COVID-19 drug candidate exploration^49^ with RotatE and DistMult, OntoProtein’s Gene Ontology-based KG for protein language model pretraining^50^, and the node-embedding algorithms for multimodal biomedical KGs^51^, enhance drug discovery and predict disease co-morbidities. Further, task-specific KGs and frameworks such as BioCypher^52^ further support KG construction, aiding predictive modelling for drug adverse reactions, repurposing and biological concept associations. One of the latest state-of-the-art biomedical KG, PrimeKG, integrates 20 primary resources including DisGeNet, MONDO Disease Ontology and DrugBank^53^, encompassing 17,080 diseases and 4 million relationships. It includes diverse disease-associated data such as protein perturbations, biological processes, pathways, phenotypes and drug therapeutic actions, including ‘indications’ and ‘contraindications’ drug–disease edges. The method TxGNN^54^ leverages PrimeKG via the R-GCN framework, to perform zero-shot inference of drug–disease indications and contraindications on diseases with no known treatment and minimal molecular understanding, efficiently handling therapeutic tasks through adaptive aggregation and iterative optimization of embeddings.

On the basis of the assumption that representing head entities in a graph while considering their path to specific tail nodes might enhance representation learning and lead to more accurate LPs, researchers started developing representation learning frameworks for LP based on the paths between two nodes. The first application of this concept is the study from ref. ^55^, which introduce KG4SL, a graph neural network (GNN) model that integrates KG message-passing for synthetic lethality (SL) prediction, leveraging a KG with 11 entity types and 24 relevant relationships associated with SL. Further, the neural Bellman–Ford network (NBFNet) introduces a framework for LP inspired by traditional path-based methods^56^. It represents node pairs as the sum of all path representations (of a given length), each derived from edge representations, and it employs a graph neural network with learned operators for efficient path formulation solutions, scalable to large graphs with low time complexity. NBFNet works with both homogeneous and multirelational graphs, supporting LP across different graph types. Combining traditional path-based methods with GNNs, NBFNet demonstrates superior performance compared with node-embedding methods. In addition, path-embedding methods offer better interpretability by visualizing important paths used for prediction, facilitating verification of biological plausibility.

To tackle LP in biological KGs, we introduce BioPathNet, a message-passing neural network for path representation learning, built on NBFNet. As opposed to node-embedding approaches, BioPathNet uses path-based reasoning to learn representations between source and target nodes on the basis of relations along the path. We introduce key design choices in BioPathNet to adapt and extend the NBFNet algorithm for LP tasks on biomedical KGs, which are inherently ‘noisy’ due to false positives from experiments, data sparsity, redundant edges and relations, and knowledge bias (for example, well-studied disease genes being overrepresented and highly connected). A crucial enhancement is the incorporation of a background regulatory graph (BRG) for message passing, which not only improves BioPathNet’s performance but also enhances its scalability. This makes biomedical LP feasible for tasks such as drug repurposing, where running NBFNet out of the box is computationally prohibitive. In addition, we implement a biological entity-type-aware negative sampling scheme, which can be combined with adaptive sampling based on local graph density. This improves decision boundary accuracy across tasks and further boosts BioPathNet’s performance. We evaluated BioPathNet on diverse biomedical KGs with varying network properties, relation types and node types, demonstrating its effectiveness in LP across multiple tasks, including gene function prediction, zero-shot disease–drug target interaction prediction, SL gene pair identification and long non-coding RNA (lncRNA)–gene regulatory inference. We show that the BRG not only improves BioPathNet’s scalability over NBFNet but also enhances performance across all tasks. In addition, the node-type-aware (NTA) negative sampling scheme improves decision boundary accuracy and further boosts performance in most LP tasks.

BioPathNet was benchmarked against the original NBFNet, three KGE baselines and seven state-of-the-art methods, including task-agnostic models for heterogeneous graphs and long-range relationship learning, as well as LP task-specific approaches. We show that BioPathNet consistently outperforms these methods across tasks or, in a few cases, achieves comparable performance.

Finally, we showcase BioPathNet’s ability to naturally interpret predictions through biological pathways and uncover biological knowledge. We illustrate this with two examples in disease–drug indication prediction, focusing on acute lymphoblastic leukaemia (ALL) and Alzheimer’s disease (AD).

## Results

### BioPathNet: path embedding as alternative to node embedding for graph completion on biomedical KGs

**Fig. 1:**
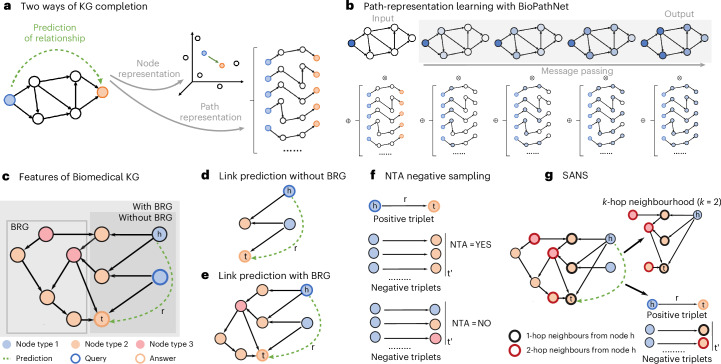
Link prediction in biological KGs. **a**, Node embedding vs path-representation learning. **b**, Illustration of how the generalized Bellman–Ford algorithm operates in NBFNet and BioPathNet to solve the shortest path problem between a head (h) and tail (t) entity through specific relationships (r). The method leverages message-passing GNNs to learn path representations, while a subsequent multilayer perceptron differentiates between positive and negative relationships (more details in Methods). **c**, Illustration of BioPathNet’s first design choice: integrating a BRG to enrich gene connections, improving message passing and information flow beyond supervised training edges. **d**,**e**, Illustration of prediction paths between head nodes (blue) and tail nodes (orange) in two scenarios: without BRG (**d**) and with BRG connections (**e**). **f**, Illustration of the NTA negative sampling procedure, where negative triplets are sampled only if their node types match the positive triplets, compared to a non-type-aware approach, which samples negatives uniformly without considering tail node type. **g**, Illustration of the SANS procedure, where negative triplets are selected on the basis of both the node type of positive triplets and the local graph density surrounding each positive triplet.

A KG is a heterogeneous directed graph comprising various types of entity (nodes) connected by relationships (edges). For instance, nodes might represent diseases, genes and drugs, and relationships such as ‘indication for’ or ‘involved in’. KGs are typically structured as triplets (head node, relationship, tail node), such as (drug A, indication, disease B) or (gene C, involved in, disease D). The task of KG completion involves estimating the missing components of these triplets. For example, one might predict the tail entities given a head entity and a specific relationship, such as predicting drugs as indications for diseases, on the basis of existing triplets (that is, existing knowledge). KG completion methods can be broadly categorized into embedding-based and path-based approaches (Fig. 1a). Embedding-based approaches encode entities in a KG into lower-dimensional spaces, preserving graph structure by minimizing distances or maximizing similarities between head, relation and tail embeddings.

A path embedding-based LP approach, instead, captures the structural information of KGs by learning representations for pairs of nodes (instead of single nodes) through paths. Intuitively, path embeddings capture logical relationships between head and tail nodes in a graph. Instead of explicitly enumerating rules, as in traditional logic-based LP, they represent paths as high-dimensional vectors and leverage neural networks for a nonlinear learning process. NBFNet^56^ learns node pair representations by parameterizing them as the generalized sum of path representations, with each path representation as the generalized product of edge representations along the path (Figs. 1b and 7). This path formulation can be efficiently solved using the generalized Bellman–Ford algorithm based on dynamic programming. Moreover, the efficiency is further enhanced by learning the operators of the generalized Bellman–Ford algorithm with a message-passing graph neural network (see Methods).

BioPathNet extends the NBFNet framework to address the unique challenges of biomedical KGs, which are large, incomplete, prone to noisy relations due to experimental errors or false positive assignments, and enriched with diverse yet often redundant information. First, on top of a primary KG (G1), for which we want to learn and generate predictions, it introduces a secondary KG, the BRG or G2. The BRG serves the goal of improving entity connectivity during message passing (Fig. 1c–e) and thereby enriching the representation of triplets through the incorporation of additional paths and relationships, without affecting loss computation, guaranteeing a scalable solution also with very large KGs. In fact, unlike NBFNet, which targets general graph completion, BioPathNet optimizes the model’s loss only on the links of interest, that is, training edges in G1 (and corresponding negative triplets). Predictions can be made without or with leveraging a BRG during the message-passing step (see Methods) BRG (Fig. 1c). For example, as illustrated in Fig. 1d, when predicting the missing link between a head node and a tail node, messages can be passed between type 1 and type 2 nodes, resulting in a certain prediction path (Fig. 1d). Alternatively, as illustrated in Fig. 1e, a BRG can be integrated, and predictions are made going through the relation edges involving node types 2 and 3. The integration of a BRG in BioPathNet enhances both prediction accuracy and interpretability, demonstrated in the following sections, by enriching the representation of the primary task. This enables the exploration of subnetworks, such as interaction partners around specific node pairs, facilitating the derivation of mechanistic hypotheses from the broader biological context. BioPathNet also introduces a stricter NTA negative sampling strategy (Fig. 1f), where negative triplets are drawn with preserved tail node types, allowing not only to improve the model’s decision boundary but also ensuring biologically meaningful sampling. In addition, to improve decision boundary, we integrate the SANS strategy from ref. ^57^ into our NTA sampling (Fig. 1g), accounting for local graph structure and density instead of uniformly sampling negative triplets.

### BioPathNet accurately predicts links across different biomedical tasks

#### Overview of the different LP tasks

We evaluate BioPathNet on biomedical LP across four distinct tasks, framing the problem as learning the distribution of logical rules from a KG, and use the learned rules to perform inference, that is, predict new links on the same graph (transductive setting) or on a new graph (inductive setting). We evaluate BioPathNet across diverse biomedical tasks, testing its adaptability to KGs of varying size, complexity, sparsity, noise and bias.

**Fig. 2:**
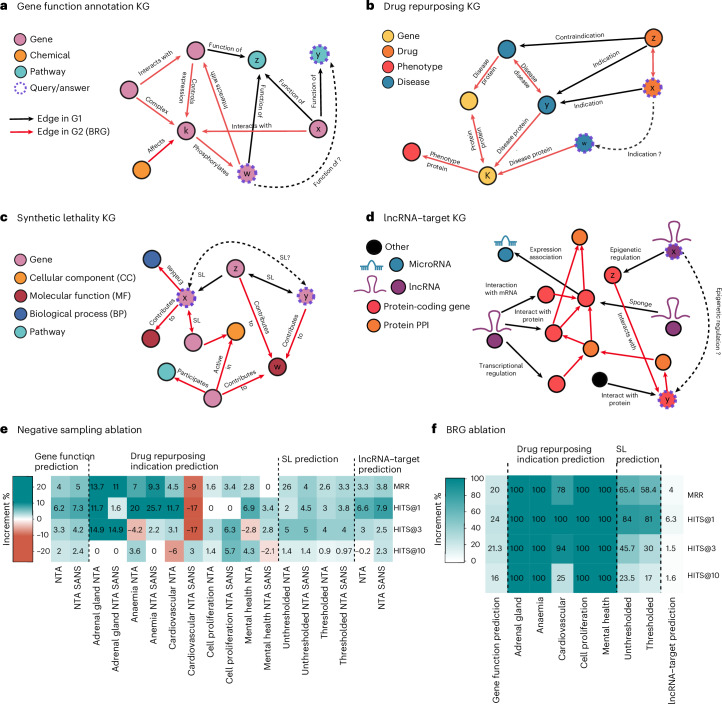
Overview of the four LP tasks across different KGs and impact of BioPathNet’s design choices on performance. **a**, Schematic of the gene function prediction KG, linking genes, chemicals and cellular pathways. Training triplets (G1: gene–pathway links extracted from KEGG) are in black; BRG edges (extracted from Pathway Commons for message passing) are in red. **b**, Simplified schematic of the PrimeKG graph for disease–drug indication prediction, connecting, for example, genes, diseases, drugs and phenotypes. G1 edges (disease–drug links) are in black; BRG edges (all other relations) are in red. **c**, Schematic of synthetic lethality KG for gene pair prediction. G1 edges (SL links) are in black; BRG edges are in red and include additional SL interactions alongside associated nodes (cellular components, molecular functions, biological processes and pathways). **d**, LncRNA-mediated regulation KG from LncTarD 2.0, with BRG from Pathway Commons. The graph features six node types (lncRNAs, microRNAs, mRNAs, pseudogenes, transcription factors and proteins), with regulatory relationships in black and BRG protein interactions in red. **e**,**f**, Ablation study results on the NTA and SANS (**e**) and on the BRG (**f**) showing performance changes vs original BioPathNet, that is, increases in green and decreases expressed as percentages in red across five seeds for each experimental task.

The first LP task we evaluate with BioPathNet is ‘gene function prediction’, which consists of assigning biological terms, such as molecular pathways, to specific genes (Fig. 2a). We use as G1, a KG linking genes to pathway terms from KEGG via the ‘function of’ relation, sourced from ConsensusPathDB^58^ and consisting of 32,000 edges (Supplementary Table 1). G1 is then split into training, validation and testing edges. During training, training triplets are used for message passing (that is, referred to as training graph from G1), while removing the specific triplets of the batch for learning path representations. This graph is also used during validation and testing, in addition to a potential G2/BRG (see below). In this context, if gene *x* has functions *y* and *z*, and gene *w* is assigned to function *z*, then gene *w* is also likely to have function *y*, and we can think of BioPathNet as learning logical rules such as: ‘function_of(y, x) ∧ function_of(z, x) ∧ function_of(z, w) → function_of(y, w)’ (Fig. 2a). Introducing a BRG for message passing (G2) (connected to the original G1, extracted from Pathway Commons^59–61^ encompassing ~1.8 million edges and 13 different relation types between genes and chemicals, including PPIs (Supplementary Tables 1 and 2)) allows BioPathNet to infer gene–function links through alternative paths, enabling the learning of more expressive rules. For example, if gene *x* has function *k*, interacts with gene *k* in a PPI network, and gene *z* phosphorylates gene *w*, then gene *w* is also likely to be assigned to function *y*. This can be expressed as the following logical rule: ‘function_of(y, x) ∧ interact(x, k) ∧ phosphorylation(k, w) → function(y, w)’ (Fig. 2a).

The next LP task predicts disease–drug indications by repurposing existing drugs for new conditions using PrimeKG (Supplementary Fig. 1a)^53^ (see Methods). This task is more challenging than the previous one, as it is conducted in a zero-shot setting—where the target disease has minimal molecular characterization and no existing treatments—following the approach from TxGNN^54^, a state-of-the-art node- and relation-based embedding method for this task (see Methods). We applied BioPathNet to five disease areas: ‘adrenal gland,’ ‘anaemia,’ ‘cardiovascular,’ ‘cell proliferation,’ and ‘mental health’, splitting the task into corresponding subtasks. BioPathNet was trained on drug–disease edges (for example, ‘indication’, ‘contraindication’) of all other diseases except for the disease area of interest, while the remaining PrimeKG graph served as the BRG (G2) for message passing. A BRG with ~5.7 million edges and 30 relation types for message passing, including drug–drug, protein–protein and disease–disease interactions from PrimeKG, was solely used for message passing (Supplementary Table 3). In this zero-shot prediction context (Fig. 2b), if drug *x* is indicated for disease *y*, which shares the same disease protein *k* with disease *w*, then drug *x* is probably an indication for *w*: ‘indication(x, y) ∧ disease_protein(y, k) ∧ disease_protein(w, k) → indication(x, w)’.

The third LP task focuses on predicting links between synthetic lethality gene pairs (Fig. 2c). SL occurs when the simultaneous mutation of two genes leads to cell death, while the mutation of either gene alone is non-lethal^62^, and this is of high interest in anti-cancer therapies. Data from SynLethDB-v.2.0, pre-processed by KR4SL^55^, includes SL pairs curated from biochemical assays, databases, predictions and text mining, with confidence scores prioritizing experimental evidence. The G1 graph contains ~20,000 gene–gene SL edges, and learning logical rules in this setting (without a BRG) corresponds to, for example: ‘synth_leth(x, z) ∧ synth_leth(z, y) → synth_leth(x, y)’, that is, if *x* and *z* are an SL pair and *z* and *y* are an SL pair, then *x* and *y* are likely to be an SL pair. When including a BRG (G2), this adds ~380,000 connections across 48 relation types, including additional SL links and gene–Gene Ontology (GO) term association links under ‘molecular function’, ‘cellular process’ and ‘cellular compartment’ categories on top of the training graph from G1. In this case, BioPathNet can learn more sophisticated rules of the type: ‘synth_leth(x, z) ∧ contributes_to(z, w) ∧ contributes_to(y, w) → synth_leth(x, y)’, where a path through genes *z* and *y* and their shared molecular function *k* under the relation ‘contributes_to’ is chosen to infer an SL relationship between *x* and *y*.

The final LP task applies BioPathNet to predicting regulatory interactions between long non-coding RNAs (lncRNAs) and genes (Fig. 2d). LncRNAs—transcripts over 200 nucleotides long that do not encode proteins—play essential roles in gene regulatory networks and are implicated in numerous diseases. However, most remain functionally non-characterized despite genomic consortia^63^ estimating over 200,000 potential transcripts. Identifying lncRNA targets is a crucial step in understanding their functions, and predictive methods are invaluable in this process to guide experiments. The G1 graph for this task was built from regulatory lncRNA–target (gene–mRNA) interactions sourced from LncTarD (2.0)^64^, which contains ~6,000 experimentally validated lncRNA–target interactions in human diseases, categorized into seven regulatory mechanisms (including ‘epigenetic’, ‘transcriptional’ and ‘sponge regulation’) (Supplementary Tables 4 and 5). The BRG (G2), sourced from Pathway Commons, is the same G2 used for gene function prediction. Since small chemical molecules lack direct links to lncRNAs, we included only the PPI component, resulting in ~1.1 million edges and 7 relation types. In this context, if lncRNA *x* regulates gene *z*, and gene *z* interacts with gene *y* in a PPI network (Fig. 2d), it is likely that lncRNA *x* also regulates gene *y*, and the corresponding logical rule can be formulated as: ‘lncRNA_regulation(x, z) ∧ interact(z, y) → lncRNA_regulation(x, y)’.

For each of the four LP tasks, BioPathNet was trained, validated and tested on parts of the data of G1 (see Methods). Performance was evaluated using the NTA negative sampling scheme, with and without G2 for message passing. In more detail, for gene function prediction, BioPathNet was trained on gene–function triplets, with negatives generated only from non-occurring gene–function pairs (same node type as positives), excluding gene–gene, function–function or gene–compound interactions to preserve positive node types. For drug repurposing, BioPathNet was trained on drug–disease indication triplets, on each disease split separately as in TxGNN^54^, to simulate zero-shot conditions, a scenario where no approved drugs for the disease of interest were seen in training. These splits provide challenging yet realistic evaluation scenarios, mimicking zero-shot drug repurposing (see Methods). Negatives were sampled exclusively from non-occurring drug–disease pairs, excluding gene–gene, disease–disease, gene–GO term and other interactions to preserve positive node types. For the SL task, BioPathNet was trained in both transductive and inductive settings on: (1) gene–gene pairs with an SL confidence score above 0.3 (thresholded data, filtered by confidence score, see Methods) and (2) gene–gene SL pairs regardless of confidence score (unthresholded data). This allowed us to evaluate the impact of noisy labels on model performance. Negatives were sampled exclusively from non-occurring gene–gene lethality pairs, avoiding gene–GO term or other cross-type triplets. For this task, we also trained BioPathNet in an inductive setting, predicting SL links between gene pairs on an inference graph distinct from the training graph, following a split based on nodes of Knowledge Representation for Synthetic Lethality (KR4SL). This setup allowed us to assess BioPathNet’s ability to generalize to unseen data. For lncRNA target prediction, BioPathNet was trained on triplets where the head node is an lncRNA and the tail node a target of various types (for example, microRNA, protein-coding gene, another lncRNA or other; see Fig. 2d), linked by a regulatory mechanism (for example, ‘epigenetic regulation’ or ‘sponge interaction’). Negatives were generated as above by corrupting positive lncRNA–target triplets while preserving node types; for instance, if a positive triplet involved an lncRNA and a protein-coding gene, only non-occurring lncRNA–protein interactions were sampled. We report for each task the mean reciprocal rank (MRR), which measures how well the model ranked correct pairs among negatives, and Hits@k (*k* = 1, 3, 10), which quantifies the proportion of positive triplets among the top-*k* predictions—both standard metrics for KG–LP problems (Table 1).

**Table 1:** BioPathNet performance on all four LP tasks, reported as MRR, and Hits@k for *k* = 1, 3 and 10

LP task	MRR	Hits@1	Hits@3	Hits@10
Gene function prediction	0.547 ± 0.002 (0.01)	0.457 ± 0.004	0.593 ± 0.006	0.723 ± 0.009
Drug–disease indication (adrenal gland)	0.83 ± 0.04 (0.0001)	0.67 ± 0.06	1.0 ± 0.0	1.0 ± 0.0
Drug–disease indication (anaemia)	0.458 ± 0.004 (0.0001)	0.417 ± 0.0	0.44 ± 0.01	0.58 ± 0.04
Drug–disease indication (cardiovascular)	0.23 ± 0.008 (0.0001)	0.19 ± 0.01	0.24 ± 0.02	0.30 ± 0.02
Drug–disease indication (cell proliferation)	0.61 ± 0.009 (0.0001)	0.55 ± 0.03	0.65 ± 0.009	0.725 ± 0.007
Drug–disease indication (mental health)	0.361 ± 0.006 (0.0001)	0.312 ± 0.005	0.35 ± 0.009	0.49 ± 0.006
Synthetic lethality, transductive, thr = 0.0	0.311 ± 0.002 (0.001)	0.1815 ± 0.0005	0.2933 ± 0.0009	0.4434 ± 0.0008
Synthetic lethality, transductive, thr = 0.3	0.3586 ± 0.0002 (0.001)	0.2151 ± 0.0004	0.3388 ± 0.0002	0.5094 ± 0.0002
Synthetic lethality, inductive, thr = 0.0	0.354 ± 0.002 (0.002)	0.209 ± 0.003	0.379 ± 0.001	0.541 ± 0.001
Synthetic lethality, inductive, thr = 0.3	0.403 ± 0.002 (0.002)	0.246 ± 0.003	0.4321 ± 0.0007	0.6083 ± 0.0007
lncRNA–target gene pairs	0.189 ± 0.002 (0.01)	0.095 ± 0.003	0.204 ± 0.003	0.383 ± 0.003

Errors represent the s.e.m. values across five seeds. Values in brackets correspond to expected MRR for random performance (random MRR) (see ‘Model evaluation’ in Methods).

#### BioPathNet’s performance and evaluation of design choices

Table 1 summarizes the results of KG completion across the four tasks, demonstrating that BioPathNet achieves an MRR consistently better than random for all LP tasks (see Methods), indicating strong predictive capability. The model performs particularly well in drug–disease indication prediction for the disease categories of adrenal gland, anaemia and cell proliferation, achieving an MRR above 0.4 in all three cases and Hits@10 scores ranging from 58% to 100%. Performance is slightly lower for other disease categories, such as ‘mental health’, with ‘cardiovascular’ diseases posing the greatest challenge, yielding an MRR of 0.2 and a Hits@10 score of 30%. While the drug repurposing tasks primarily focus on predicting disease–drug indications, BioPathNet also performs well in identifying disease–drug contraindications, with an average MRR of 0.53 (Supplementary Table 6). BioPathNet performs well on the gene function prediction task, achieving an MRR of 0.6 and a Hits@10 score of 72%. For the SL pair prediction task, performance improved substantially when low-confidence SL edges were filtered out from the graph, with MRR increasing from 0.31 to 0.36 and Hits@10 rising from 44% to 50%. Finally, for the lncRNA–target prediction task—one of the most challenging due to graph sparsity and relational uncertainty—BioPathNet achieved an MRR of 0.19, outperforming random predictions. These results underscore BioPathNet’s versatility in handling diverse LP tasks across different biomedical KGs, regardless of task-specific complexities.

To assess the impact of key design choices, such as BioPathNet’s NTA negative sampling or SANS, as well as the use of a BRG, we conducted ablation studies. We compared performance with and without NTA and SANS and evaluated BioPathNet with and without a BRG for message passing alone on all four benchmarked tasks. BioPathNet benefits significantly from incorporating NTA sampling and BRG (Supplementary Tables 6–9). In the gene prediction task, sampling negatives from the same node type as the tail node improves MRR by 4% and Hits@k by 2–6.2% compared to random sampling (Supplementary Table 7). Similar gains are observed for SL and lncRNA–target KGs (Supplementary Tables 8 and 9, respectively). For drug–disease indication prediction, MRR improves across all disease splits, with increases ranging from 1.6% (cell proliferation) to 13.7% (adrenal gland), alongside larger Hits@k improvements compared with other tasks in almost all cases (Supplementary Table 6). While applying SANS alone resulted in minimal performance gains, the greatest improvement occurred when SANS—accounting for local graph structure during negative sampling—was combined with NTA sampling (Fig. 2e). This combination enhanced performance in gene function, SL and lncRNA–target LP tasks. However, for drug–disease indication prediction (and contraindication), the benefits were only partially observed across some metrics, with a notable exception in the cardiovascular disease split, where SANS reduced prediction performance. In addition, SANS sampling was computationally expensive due to the random walk search (even when limited to length 2, see Methods), so we report experiments with this setting disabled for the remaining part of the section.

Using a BRG for message passing in addition to the training graph of G1 improved performance across all tasks (Fig. 2f and Supplementary Table 10), with metrics gains ranging from 1.5–6.3% for lncRNA–target target prediction, 16–24% for gene–function prediction, and up to 84% for SL. In the zero-shot disease–indication LP task, removing the BRG and running BioPathNet solely on the G1 bipartite training disease–drug graph severely disrupted connectivity. In fact, here we tackle a zero-shot drug repurposing problem, where a disease in the test set is simulated by removing all known treatments and similar diseases in the training set, making message passing very difficult. This prevented BioPathNet from identifying meaningful paths for prediction and led to a drastic performance drop—an expected outcome by design. Given that this task is originally meant to make full use of the PrimeKG graph^53^ (training graph from G1+BRG), we considered the ablation experiment unfair in this context.

**Fig. 3:**
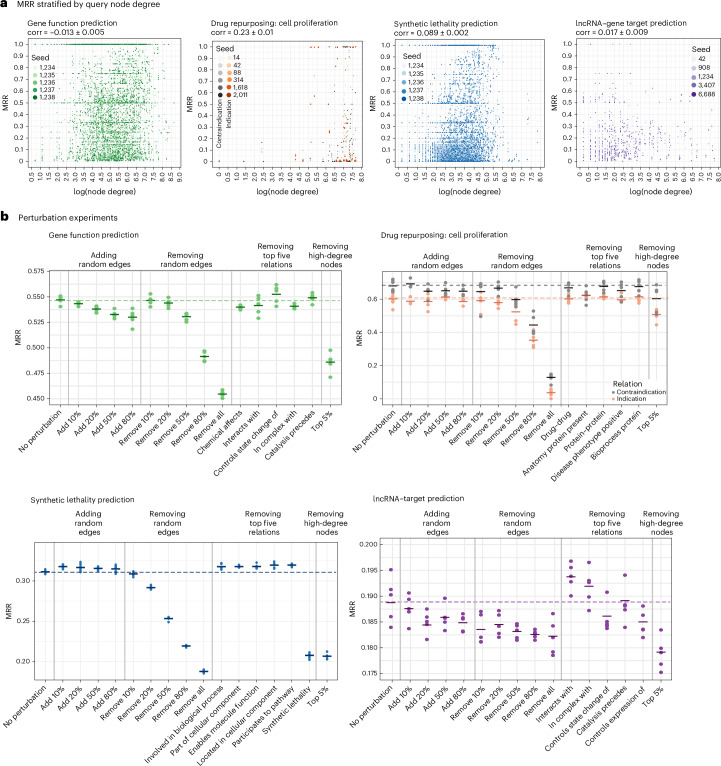
Bias evaluation and edge perturbation. **a**, MRR on the test set from G1 stratified by query node degree across the four LP tasks, with scatterplots showing values from five seeds and average Spearman correlation ± s.e. **b**, MRR on the test set for the four LP tasks following systematic BRG edge perturbation, including progressive random edge addition and edge removal at varying percentages, as well as individual removal of the top five relation types.

To evaluate BRG quality and potential biases in LP, we conducted perturbation experiments. Biological networks, such as PPIs, often exhibit study bias, where well-studied disease-related proteins have higher node degrees. We first analysed bias toward high-degree nodes, a common issue in LP, where models favour well-connected nodes, potentially reducing generalization to low-degree nodes. By computing and visualizing MRR stratified by node degree across all tasks (Fig. 3a), we find no correlation between performance and query node degree for three of the four tasks: gene function prediction, SL and lncRNA, suggesting no bias toward genes, lncRNAs or pathway hubs. However, for drug repurposing, we observe a weak positive correlation (0.23), indicating a tendency of the model to associate drug indications with well-characterized drugs or diseases.

We next assessed the impact on BioPathNet performance (measured by the MRR) of introducing random noise into the BRG by either adding random edges at varying proportions (10–80%) or removing edges at varying proportions (10–100%), as well as removing the top 5% highest-degree nodes. In addition, we investigated whether specific relation types contribute more to performance or whether BioPathNet primarily benefits from the overall connectivity provided by the BRG. To test this, we removed the top 5 relation types (based on edge count) for each task and evaluated performance changes. The results show that the effects of these perturbations vary by task and depend on the specific BRG (Fig. 3b). For gene function prediction, adding random edges slightly decreases performance, suggesting BioPathNet’s robustness to noise. However, the biggest performance drops occur with progressive random edge deletions, ultimately reducing performance to levels seen without the BRG (Fig. 3b, top left corner). The impact of removing individual relation types on BioPathNet’s performance for gene function prediction varied by relation. Some had a greater effect than others, particularly ‘chemical affects’ (gene–chemical compound interactions), ‘interacts with’ (protein–protein interactions) and ‘in complex with’ (protein complexes). For drug repurposing, adding random edges had no significant impact on BioPathNet’s performance, while performance degradation was only observed when 50–80% of random connections were removed (Fig. 3b, top right corner). Among the top 5 relations, removing individual relation types from the PrimeKG BRG had no noticeable effect for drug–disease indication prediction, except a modest decrease for ‘disease phenotype positive’, indicating whether a disease is linked to the presence of a related phenotype. The same relation has an even stronger effect on the performance of contraindication prediction, together with relation type ‘anatomy protein present’, which specifies the body part or tissue where a gene is expected to be expressed or, conversely, absent. Although we cannot exclude the possibility that any other relation type of the 25 remaining relation types from PrimeKG might have a stronger effect on BioPathNet performance, these results suggest that biomedical KGs exhibit redundancy, with certain connection types being more critical than others for this task.

For the lncRNA–target prediction task, we observe trends similar to those in gene function prediction, where adding or removing edges leads to modest but consistent performance declines (Fig. 3b, bottom right corner). In addition, different BRG relation types, such as ‘control state change of’ and ‘controls expression of’, appear more informative compared with gene function, pointing to the regulatory relationship being more relevant in the context of lncRNA–target gene regulation.

Finally, across all tasks, we observe a substantial performance drop when removing the top 5% of high-degree nodes from the BRG. This aligns with expectations, as predictive paths are more likely to traverse network hubs. For SL, the BRG, constructed by the authors of KR4SL, includes various gene, pathway and GO relations, along with an additional SL-specific component. Our findings suggest that non-SL relations in the BRG do not improve performance and may even be detrimental. Instead, only the additional SL component significantly enhances performance, highlighting the overall low quality of the SynLethDB graph (Fig. 3b, bottom left corner). Overall, our analysis suggests that the connectivity of the graph, augmented by the BRG, seems to be more critical for performance, rather than individual relation types. To test this, we computed the average shortest path between head and tail nodes (as a proxy for connectivity) across all perturbation experiments for each LP task. We observed a negative correlation between task-specific MRR performance and shortest path length (−0.15 and −0.31 for drug repurposing, contraindication and indication; −0.06 for gene function; −0.33 for lncRNA–target prediction; and −0.58 for SL). These findings support our hypothesis that performance drops are primarily driven by disruptions in graph connectivity.

### Comparison with baselines

We compared BioPathNet’s performance to various baselines, including general LP models applied across all four LP tasks and task-specific methods designed for individual LP challenges. We selected general-purpose baselines to evaluate their ability to handle various biomedical tasks compared to BioPathNet, which is also designed and tested on different LP tasks. In contrast, task-specific baselines are optimized for a particular task, potentially giving them an advantage on that task over more generalized models.

#### BioPathNet vs general GNN-based LP method

We systematically compared BioPathNet to general LP prediction methods, such as R-GCN, which generates node embeddings on heterogeneous graphs; HGT, a GNN-based framework with node- and edge-type dependent attention for heterogeneous graphs; and RAGAT, a GAT-based model incorporating node- and edge-type-aware message passing. Finally, we compared BioPathNet to the original NBFNet across all tasks. To ensure fairness, baselines were trained on the full KG (training graph from G1+BRG) whenever computationally feasible. For R-GCN, RAGAT and HGT, we also applied the same NTA-based negative sampling as BioPathNet, allowing a direct assessment of node embedding on heterogeneous graphs versus path embedding, without confounding effects from differing negative sampling strategies. Hyperparameter tuning to find the best-performing configuration was done for all general baselines, when possible, to utilize these methods at their best (Extended Methods ‘Baseline methods used for comparison’).

For SL and lncRNA prediction, BioPathNet and NBFNet performed similarly across metrics. BioPathNet slightly outperformed other baselines on SL prediction, while HGT matched its MRR on the lncRNA task but lagged in Hits@3–10. Interestingly, while BioPathNet and NBFNet remained consistent across tasks, HGT struggled with SL prediction, showing the lowest performance, whereas RAGAT performed surprisingly well. Conversely, for lncRNA prediction, HGT was clearly superior to RAGAT. Overall, R-GCN was the weakest performer across all LP tasks.

On the function prediction task, BioPathNet outperformed all KGE baselines. It achieved a sustantial performance increase, averaging 42% higher MRR and 24% higher Hits@10 over RAGAT, the weakest baseline. However, its improvement over HGT, the second-best model, was modest, with only a 2.2% gain in MRR and 3.2% gain in Hits@10. HGT remained competitive, showing performance close to that of BioPathNet across all evaluated metrics (MRR and Hits@1,3,10).

**Fig. 4:**
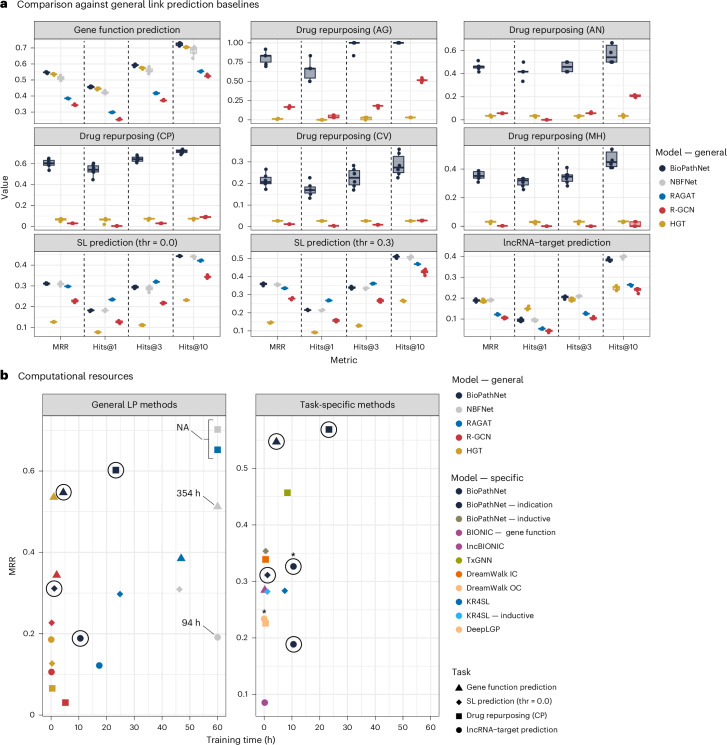
Comparison with general LP baselines across tasks and runtime analysis. **a**, BioPathNet benchmarked against R-GCN, HGT, RAGAT and NBFNet across multiple LP tasks, including gene function prediction, drug repurposing (indications for adrenal gland (AD), anaemia (AN), cell proliferation (CP), cardiovascular (CV) and mental health (MH)), synthetic lethality gene pair prediction (unfiltered, thr = 0; filtered, thr = 0.3) and lncRNA–target prediction. Performance metrics include MRR and Hits@1, 3, 10 for each task. Individual data points represent different seeds (*N* = 5) and are summarized in boxplots, with the bottom and top edges indicating the 25% and 75% percentiles, the line in the box representing the median, and whiskers extending to 1.5× the interquartile range on both sides. **b**, MRR vs training time (hours) for BioPathNet compared to general LP methods across all four tasks (left) and task-specific baselines (right). The circles highlight BioPathNet, and the asterisk indicates that, for the lncRNA interaction task, the KG made from the node intersection between DeepLGP and BioPathNet was used to ensure a fair comparison.

For the more challenging drug repurposing task, specifically, zero-shot disease–drug prediction, BioPathNet demonstrated a clear advantage over GNN-based methods such as HGT and R-GCN across all disease splits. Our experiments show that these methods struggle to capture the complexity of the KG, often yielding extremely low performance, with some metrics approaching random values (Fig. 4a). In addition, due to the large scale of the drug repurposing graph (~5 million edges), baselines such as RAGAT and NBFNet were computationally infeasible, requiring over 48 h per epoch on GPUs. Since computational efficiency is crucial in practical applications, we analysed the relationship between model training time (as a proxy for efficiency) and performance. While it became obvious that node embedding-based methods such as R-GCN and HGT are computationally efficient for biomedical LP tasks, compared to path-based methods, even on large graphs, this efficiency comes at the cost of lower performance in most cases (Fig. 4).

Although NBFNet performed similarly to BioPathNet on smaller tasks such as lncRNA–target prediction and SL, its training time was, on average, 80 times higher (for example, gene function prediction). A similar trend was observed for RAGAT, which had, on average, the second-highest runtime across all baselines. In addition, despite utilizing GPU resources, NBFNet was infeasible to run on the drug repurposing task, and RAGAT failed on both drug repurposing and gene function prediction due to memory limitations, with per-epoch training times exceeding 48 h. Overall, BioPathNet demonstrated a strong balance between accuracy and computational efficiency.

#### BioPathNet vs task-specific baselines

**Fig. 5:**
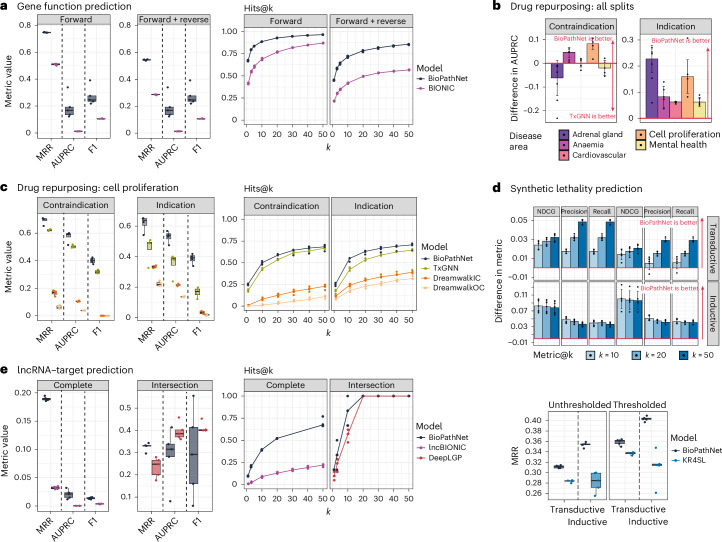
Benchmarking BioPathNet against task-specific baselines. **a**, Performance comparison between BioPathNet and BIONIC for gene function prediction, reported as MRR, AUPRC and F1 (left), and Recall@k (right), evaluated on both forward and forward+reverse edges. **b**, Difference in AUPRC values between BioPathNet and TxGNN across disease area splits (adrenal gland, anaemia, cardiovascular, cell proliferation, mental health). **c**, Drug repurposing performance comparison between BioPathNet, DREAMwalk_IC (inferred connections) and DREAMwalk_OC (original connections), reported as MRR, AUPRC and F1 (left), and Recall@k (right), evaluated on both indication and contraindication edges. **d**, Top: performance differences between BioPathNet and KR4SL in both transductive and inductive settings, evaluated on unfiltered (thr = 0, left panels) and filtered (thr = 0.3, right panels) data for NDCG, precision and recall at different *k* values. Bottom: MRR comparison between BioPathNet and KR4SL in both transductive and inductive settings. **e**, Performance comparison of BioPathNet with lncBIONIC and DeepLGP for lncRNA–target prediction, reported as MRR, AUPRC and F1 (left), and Recall@k (right). In all plots, individual data points (corresponding to different seeds, *N* = 5) are shown. Box bounds are defined in Fig. 4. Bar plots display the mean, with error bars representing the standard deviation.

Gene function prediction has been approached through various paradigms, but only recently have deep learning methods framed it as an LP task, assigning biological functions to genes via pathway or GO term annotation. To benchmark BioPathNet for this task, we compared it to BIONIC, a proven method for generating gene embeddings from multiple networks and applying them to downstream tasks, particularly gene function assignment in a semi-supervised setting. We trained and evaluated BIONIC using the same data split as BioPathNet and optimized its settings for peak performance (see Extended Methods). Since BIONIC classifies genes into functional categories, we assessed both models on forward relations (gene-to-function) and the bidirectional setting used in BioPathNet. While BIONIC trained much faster (0.04 min per epoch, Fig. 4b, right panel), BioPathNet consistently outperformed it across all metrics (Fig. 5a).

We benchmarked BioPathNet on drug repurposing, predicting drug–disease indications and contraindications in a zero-shot setting, against two state-of-the-art models, DREAMwalk and TxGNN. DREAMwalk is a deep learning framework that predicts drug–disease indications by integrating associations through a random walk algorithm to identify therapeutic connections^42^. TxGNN^54^ is a state-of-the-art graph neural network model designed to predict drug–disease relationships in zero-shot scenarios, which builds embeddings of nodes and relations from PrimeKG (Supplementary Fig. 1a)^53^. As the hierarchical graph used in DREAMwalk was unavailable, we reconstructed it via two methods (see Methods), resulting in two versions: DREAMwalk_IC (inferred connections via semantic similarity) and DREAMwalk_OC (importing original PrimeKG connections). Following TxGNN’s data split^54^ (commit ‘1000aac’), we evaluated BioPathNet, DREAMwalk and TxGNN on five zero-shot disease areas: adrenal gland, anaemia, cardiovascular, cell proliferation and mental health. For each split, we assessed performance in predicting ground-truth drugs for ‘indication’ and ‘contraindication’ relations, ranking drugs (tail nodes) on the basis of their probability *p*(drug∣disease, relation), for all three methods. BioPathNet outperformed DREAMwalk across all tasks and achieved higher area under the precision-recall curve (AUPRC) value than TxGNN in two of five disease areas for contraindications and all five for indications (Supplementary Fig. 1b). The performance difference, *Δ* (Fig. 5b), calculated as BioPathNet’s AUPRC minus TxGNN’s, was positive for indications (5.9–22.6 percentage points). For contraindications, TxGNN performed better in adrenal gland, cardiovascular and mental health with *Δ* values of −4.6, −0.2 and −2.0 percentage points, respectively. Aggregating AUPRC across indications and contraindications, BioPathNet surpassed DREAMwalk by 61% and TxGNN by 20.2% on average (Supplementary Table 11). Despite being the least performant model, DREAMwalk remains notable for its efficiency, significantly outperforming random predictions while requiring minimal training time, making it a viable option for practical applications (Fig. 4b, right panel) A detailed breakdown of BioPathNet, DREAMwalk and TxGNN in terms of MRR, AUPRC, F1 and Recall@k for the cell proliferation disease area split is illustrated in Fig. 5c. BioPathNet outperformed DREAMwalk across all metrics and exceeded TxGNN in indication prediction for cell proliferation. This trend was consistent across all disease splits, with BioPathNet demonstrating a more substantial performance gain in indication prediction compared with contraindication (Fig. 5b and Supplementary Table 11). In the cell proliferation split, 178 diseases had known indications, with an average of 5.58 indications per disease. For 60 out of 178 diseases, all known treatments were prioritized within the top 10 predictions sampled from a list of 7,000 drug candidates. Recall@k quantifies the proportion of ground-truth items found within the top-*k* predictions. For instance, at *k* = 20, the recall for indication across all diseases was 0.619 for BioPathNet, meaning that 61.9% of the ground-truth drugs were found within the top 20 predictions. In contrast, Recall@20 for TxGNN was 0.539 and 0.259 for DREAMwalk.

For the SL task, we benchmarked BioPathNet in both transductive and inductive settings against KR4SL^55^, a state-of-the-art GNN-based method that leverages path-representation learning to predict and explain SL gene pairs (Extended Methods). We tested thresholds from 0 to 0.8 (in 0.1 increments) from SythLethDB, corresponding to increasing confidence scores on reported SL interactions, to assess their impact on BioPathNet and KR4SL (Supplementary Fig. 2b). However, our primary evaluation focused on thresholded data (SL confidence score >0.3, to balance data quality and performance) and unthresholded data, which corresponds to the original KR4SL dataset with unfiltered interactions. BioPathNet outperformed KR4SL in MRR across both transductive and inductive settings, as well as for both thresholded and unthresholded data (Fig. 5d), while having a considerably lower training time compared with KR4SL (Fig. 4b). While gains in NDCG@k, Precision@k and Recall@10-20-50 were modest (Fig. 5d, and Supplementary Tables 4, 5, 13 and 14), they remained consistently positive.

Despite the availability of databases on RNA interactions, global methods for predicting lncRNA–target interactions remain limited, with most focusing on specific interaction types. BioPathNet addresses a broader challenge: prioritizing lncRNA–target interactions (physical, regulatory, direct and indirect) using LP. Among recent deep learning frameworks, we selected DeepLGP^65^ for benchmarking due to its superior performance in lncRNA–target classification. DeepLGP integrates genomic location, expression and miRNA interactions to construct a gene interaction network. It employs a GCN encoder to extract features, which are combined with original lncRNA and gene features and processed by a convolutional neural network classifier. Trained on LncRNA2Target v.2.0, DeepLGP utilizes additional public datasets for feature computation. Since BioPathNet and DeepLGP use different datasets, each model was trained separately but evaluated on a shared test set. In addition, we adapted BIONIC, originally designed for gene function prediction, for lncRNA–target prediction (lncBIONIC). BioPathNet was benchmarked against lncBIONIC, following the same training procedure and evaluating both forward and forward+reverse interactions (see ‘Comparison with baselines’ in Extended Methods). lncBIONIC achieved a test set MRR of just 0.04, significantly lower than BioPathNet’s 0.19, and performed worse across all metrics (Fig. 5e). However, it still demonstrated some predictive capability despite not being designed for this task. DeepLGP, while achieving a lower MRR than BioPathNet (0.25 vs 0.33 on the intersection test set), nearly matched BIONIC in Hits@k and outperformed it in AUPRC and F1 score (Fig. 5e). Overall, task-specific baselines performed better for their intended tasks than general LP methods, even when compared with more general complex models.

### BioPathNet’s interpretability and human-curated evaluation

One key advantage of NBFNet is its ability to provide interpretable predictions through paths, which are crucial for understanding the rationale behind specific predictions. Intuitively, these interpretations should highlight paths that influence the prediction the most. In BioPathNet, this follows the NBFNet framework (see Methods), where the top-*k* path interpretations for a prediction are formally defined as the first derivative (gradient of the prediction) with respect to each path between a head and a tail node. We present below a general overview and quantitative evaluation of the most important nodes and node types in top explanation paths across the different LP tasks, aimed at assessing the biological plausibility of the model’s outputs. In addition, we provide several interpretability examples, each illustrating how BioPathNet’s top-ranked paths for a given triplet can be naturally understood.

#### Global explanation analysis

We conducted a global analysis of the key node types driving top predictions for a specific LP task. For each predicted triplet, we selected the top 50 test-set predictions consistently recovered in at least 3 out of 5 random seeds, ensuring robustness before extracting explanations. For each, we identified the top 10 most influential explanation paths (see Methods), then summarized the most frequent node types across these paths (Supplementary Fig. 3), highlighting the 5 most commonly traversed nodes within each type. This analysis was performed for all 5 disease-specific drug indication tasks and synthetic lethality prediction. We excluded aggregation for tasks involving general cellular processes (for example, gene or lncRNA function prediction), where the broader functional diversity made such summaries less informative.

Our results show that node types such as ‘drug’ and ‘disease’ from PrimeKG most strongly explain disease–drug indications, followed by ‘gene’ and ‘phenotype’ node types (Supplementary Fig. 3). From a qualitative assessment, the most frequent nodes along explanation paths align well with the prediction tasks. For example, in the cell proliferation disease split, top disease nodes relate to cancer subtypes or processes such as somatic mutations and uncontrolled growth, while top drug nodes such as tetramin, rucaparib and altretamine are chemotherapeutics used in oncology, suggesting that the model repurposes existing cancer drugs plausibly. In the mental health task, top-ranked nodes include antipsychotic drugs and central nervous system-related genes (for example, *HTR1D*, *HTR1F*, *HDAC6*) associated with depression, anxiety and neurological disorders^66,67^. While less dominant than ‘disease’ and ‘drug’ nodes, ‘phenotype’ and ‘anatomy’ types play a notable role, especially in mental health (where drugs often treat symptoms such as seizures) and anaemia (where liver-related phenotypes are common), as shown in Supplementary Fig. 3.

Consistent with perturbation experiments on synthetic lethality prediction, gene–gene interactions, and thus the ‘gene’ node type, are the primary drivers of BioPathNet’s predictions, with much smaller contributions from ‘cellular component (CC)’, ‘molecular function (MF)’, ‘biological process (BP)’ and ‘pathway’. Despite their lower overall influence, the most frequently traversed nodes are highly relevant to cancer. Notably, proto-oncogenes *KRAS*, *NRAS* and *HRAS*—key members of the RAS family and frequently mutated in cancer—are among the top contributors. Other influential nodes include the plasma membrane, crucial for cancer cell homeostasis, and signalling pathways such as the RAS/BRAF cascade, which are central to tumour progression.

To more quantitatively compare our explanations with established biomedical knowledge, we conducted overrepresentation analysis on the most influential genes—those most frequently appearing in the top 10 explanation paths (as described above). Specifically, we analysed DisGeNET terms, including human diseases, symptoms and associated molecular mechanisms from the literature, for four disease splits from the drug repurposing task, and KEGG pathways for the synthetic lethality and cell proliferation tasks, the latter to enable a better comparison with known cancer pathways. Results, shown in Supplementary Fig. 4, highlight the top 10 enriched (adjusted *P* < 0.01 from Fisher’s exact test, Benjamini–Hochberg correction) and least enriched terms (as controls). We observe strong concordance between our explanation nodes and established knowledge: for example, statistically significant terms align with known KEGG cancer pathways in the synthetic lethality and cell proliferation tasks. DisGeNET analysis further supports the biological relevance of our explanations: genes from top explanation paths are highly enriched for disease-relevant terms. In the cardiovascular diseases split, enriched terms include ‘hypertensive disease’, ‘atherosclerosis’, ‘coronary heart disease’, ‘myocardial infarction’ and ‘hypertension’. Similar patterns emerge for mental health, adrenal gland and anaemia, reinforcing the plausibility and domain alignment of the model’s explanations.

#### Case study 1: interpreting disease–drug indication predictions for ALL

**Fig. 6:**
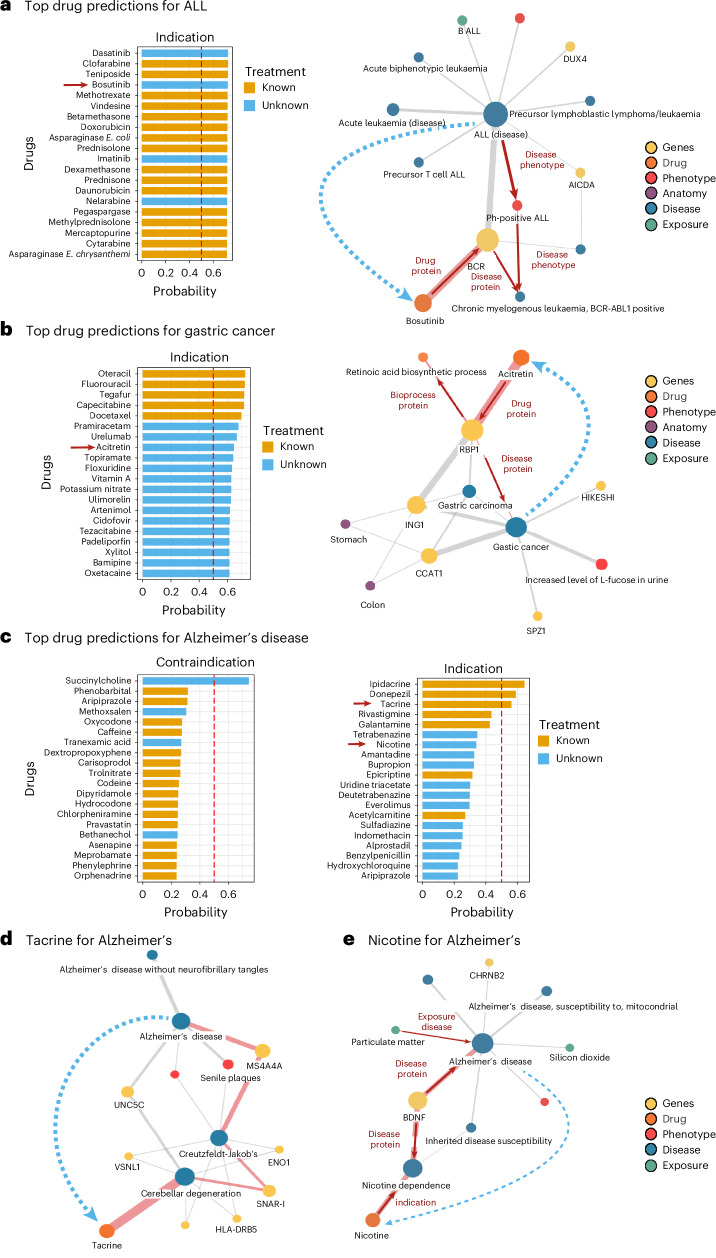
BioPathNet uncovers drug–disease indications and molecular mechanisms. **a**,**b**, ALL (**a**) and gastric cancer (**b**) in the cell proliferation domain. Left: predicted drug indications ranked by BioPathNet probability (orange, known; light blue, newly predicted). Right: gradient-based path importance for bosutinib in ALL (**a**) and acitretin in gastric cancer (**b**), showing the top ten most important paths (edge width = weight defined as the number of paths that pass through this edge, highest-weight path in red). **c**, Top 20 contraindications and indications for Alzheimer’s disease, ranked by BioPathNet probability (orange, known treatments in PrimeKG; light blue, newly predicted). **d**,**e**, Path importance visualizations for tacrine (**d**, known) and nicotine (**e**, newly predicted), showing the top ten most important paths (edge width = weight defined as the number of paths that pass through this edge, highest-weight path in red).

As a first example, in the context of the drug repurposing task, we closely examined individual disease predictions within the cell proliferation split, focusing on drug associations for ALL, a complex cancer characterized by the abnormal proliferation of lymphoid cells in the blood and bone marrow, leading to impaired immune function^68^. BioPathNet correctly ranked the only known contraindication, Aprostadil, at rank 1 with a probability score of 0.73 and placed all 21 known indications within the top 34 predictions (Fig. 6a). Among the highest-ranked known treatments (in orange) were clofarabine, teniposide and methotrexate, while the top-ranked unknown treatments (in blue) included dasatinib and bosutinib (Fig. 6a, left). Dasatinib, ranked with a probability score of 0.724, is absent in PrimeKG but is already used for Philadelphia chromosome-positive ALL (Ph+ ALL). Bosutinib, predicted for ALL with a probability score of 0.721, was further analysed through path visualization to explain its ranking.

While BioPathNet uses all paths from source to target node for the prediction, the visualization highlights the ten most important paths ranked by gradient in an ad hoc pseudo-linear explanation, with edge width indicating recurrence across these paths. The most important path, marked in red, links bosutinib to ALL through key disease genes *AICDA* and *DUX4* (refs. ^69–71^). This path traverses ‘Ph+ ALL’, ‘chronic myelogenous leukaemia’, ‘BCR-ABL1 positive’ and the gene *BCR*, before connecting to ‘bosutinib’ via the ‘drug protein’ relation (Fig. 6d, right). Originally approved for chronic myeloid leukaemia in 2012^72,73^, bosutinib is now being investigated for ALL^74^.

#### Case study 2: hypothesis generation for treatment of gastric cancer

To demonstrate BioPathNet’s ability to generate hypotheses for lab testing and evaluation, we investigated gastric cancer. Similar to ALL, both known contraindications and indications were ranked highly (all 5 contraindications in the top 6, and 5 out of 6 indications in the top 5) (Fig. 6b, left). One previously undiscovered drug predicted for gastric cancer treatment was acitretin, an oral retinoid similar to Vitamin A, indicated for skin diseases such as psoriasis^73^. Although untested for gastric cancer, acitretin has been considered in combination with clarithromycin for cutaneous squamous cell carcinoma due to its apoptosis-inducing properties^75,76^. Interestingly, all paths from gastric cancer to acitretin in the interpretability plot pass through *RBP1* (retinol-binding protein 1), annotated with the ‘retinoic acid biosynthesis process’ (Fig. 6b, right). Recent studies suggest this pathway’s involvement in gastric cancer treatment and provide pre-clinical evidence supporting the use of all-trans retinoic acid (ATRA)^77,78^. By visualizing important paths between drugs and diseases, researchers can verify the predictions’ plausibility and generate hypotheses for further laboratory validation.

#### Case study 3: predicting drug indications for AD

For the final experiment in the drug repurposing task, we aimed to investigate a disease not analysed by TxGNN to evaluate how well BioPathNet generalizes to another case study. Here we examined the indication predictions for Alzheimer’s disease together with medical experts. AD is a neurodegenerative disorder characterized by extracellular amyloid beta and intracellular tau protein accumulation in the brain. While AMYLOID and TAU proteins are central to AD, the exact mechanisms remain unclear. Emerging evidence suggests that additional pathways, such as immunoinflammation and bioenergetic dysregulation, may offer promising therapeutic targets^79–81^. Presently, US Food and Drug Administration (FDA)-approved treatments include only two disease-modifying and five symptomatic treatments, none of which provide a cure for AD. To explore the potential of BioPathNet for such complex and heterogeneous diseases, we trained BioPathNet on a data split tailored for zero-shot prediction on a custom-defined Alzheimer’s disease area split. Here we followed the disease evaluation code as provided by TxGNN to exclude all treatments for Alzheimer’s, as well as closely related diseases (for example, dementia) (Supplementary Table 15). We then evaluated the top 20 predictions for indications and contraindications for Alzheimer’s disease.

Among the top 14 predictions, BioPathNet successfully retrieved 7 out of 8 drugs classified as known treatments in PrimeKG, including 4 out of 7 FDA-approved AD treatments (Fig. 6c). It also identified epicriptine, a nootropic with an unknown mode of action, and acetylcarnitine, involved in *β*-oxidation of fatty acids^82^. Known AD drugs with low probability scores included pramiracetam (ranked 344), memantine (ranked 412), lecanemab (ranked 2,250) and aducanumab (ranked 2,216)^83–86^. Notably, two drugs in clinical trials, nicotine (Phase II trial for cognition; NCT02720445) and bupropion (Phase III trials for agitation in AD; NCT05557409, NCT04947553)^87^, were among the top 20 newly predicted indications. These predictions were linked to *BDNF*, a gene crucial for synaptic maintenance and plasticity^88^, with both drugs shown to elevate BDNF protein levels^89,90^. In addition, everolimus, a selective mTOR inhibitor, was predicted with high probability. Rapamycin and its analogue everolimus are being evaluated for AD treatment^91,92^, with everolimus offering favourable pharmacokinetics^93^. Therefore, everolimus may present an additional candidate for targeting the hyperactivated mTOR pathway in AD.

#### Additional examples

Supplementary Figs. 5–7 provide additional examples of BioPathNet’s predictions and their biological interpretation in gene function prediction, SL pairs and lncRNA–target target prediction. For instance, the association between *CRY1* and the ‘circadian rhythm’ pathway is explained by the key path highlighted in red: CRY1 protein in complex with → PER3 interacts with → ARNTL function of → ‘Circadian rhythm’ (Supplementary Fig. 5). This path is biologically meaningful, as it captures the well-established mechanism by which the essential transcription factors ARNTL and CLOCK regulate circadian rhythm. They upregulate *PER3* and *CRY2* gene expression^94,95^, which then form heterodimers to repress their own transcription, establishing a negative feedback loop^96,97^.

In SL prediction, two notable examples involve putative SL gene pairs consistently identified across model runs (seeds) with an average MRR of 0.75. The first pair, *EYA4* and *MUS81*, connects transcription, eye development and DNA repair with a key DNA repair enzyme (MUS81). BioPathNet ranks *MUS81* as *EYA4* genes as the seventh strongest SL partner (Supplementary Fig. 6c), revealing an explanation subgraph with multiple paths through shared processes such as DNA repair, supporting their SL relationship (Supplementary Fig. 6a). The second gene pair, *POLB* and *BRCA1*, involves POLB, a base-excision repair polymerase linked to Werner syndrome and oesophageal cancer. BioPathNet consistently ranks *BRCA1* among *POLB*’s top 20 SL gene partners (Supplementary Fig. 6d). The explanation subgraph (Supplementary Fig. 6b) highlights the top 10 paths between *POLB* and *BRCA1*, emphasizing shared biological processes such as DNA repair, replication, cellular homeostasis and apoptotic signalling, relevant to SL in cancer.

For the lncRNA–target prediction task, we analysed PVT1, a MYC regulator frequently overexpressed in cancers and critical for tumour progression. The top 5 novel predictions are reported in Supplementary Fig. 7a,b, together with the top 10 most relevant paths, ranked by gradient (Supplementary Fig. 7c–g). BioPathNet inferred relationships for all 5 predictions through bipartite graphs, with PVT1 and other lncRNAs clustering separately from target and co-regulated genes, reflecting the biological principle that genes in the same regulatory network share common factors.

Literature supports PVT1’s involvement in cervical cancer via EZH2-mediated repression of MIR200B^98^ and its potential role in melanoma through MIR200C^99^. PVT1–EZH2 regulation appears in all 5 predictions (Supplementary Fig. 7c–g), highlighting EZH2 protein’s role in PVT1 regulation across cancers such as gastric, thyroid, glioma and hepatocellular carcinoma. BioPathNet also predicts an interaction between PVT1 and SUZ12 (Supplementary Fig. 7a,b), a PRC2 member, identifying a path via APTR, an oncogenic lncRNA that represses CDKN1A/p21 through PRC2, involving EZH2 and SUZ12.

## Discussion

Biomedical KGs structure information by representing entities (genes, proteins, diseases, drugs) as nodes and their relationships (interactions, associations, regulations) as edges. Despite high-throughput experiments, many relationships in these graphs remain undiscovered. Link prediction (LP) methods are crucial for inferring missing or potential associations by analysing network topology.

In this work, we introduce BioPathNet, in which we extend NBFNet^56^, a message-passing neural network designed to leverage the power of path representation learning for LP on biomedical KGs. BioPathNet introduces several advancements compared to NBFNet, including the use of a BRG for improved message passing and a NTA negative sampling strategy to improve learning precision and address graph heterogeneity, design choices that were crucial to improving the performance of specific tasks. We evaluated BioPathNet on LP tasks across diverse biomedical KGs, varying in size, sparsity and quality, including gene function assignment using KEGG pathways from Pathway Commons, zero-shot disease–drug indication prediction from PrimeKG, SL gene pair prediction from SynLethDB, and inference of regulatory relationships between lncRNAs and target genes from LncTarD 2.0. Our results demonstrate BioPathNet’s versatility in biomedical LP, consistently performing better than random, with varying degrees of success depending on the task. Ablation studies further highlight the advantages of key design choices, such as NTA negative sampling and the use of a BRG for message passing. Unlike standard NBFNet, NTA enables biologically meaningful sampling by ensuring that negative triplets share the same node type as positives, improving MRR performance by 5–9% across tasks and up to 15–20% for Hits@1–3 in specific cases. Since Hits@k measures how often positive triplets rank among the top predictions, this improvement is especially crucial for biological practical applications, where top-ranked predictions may be prioritized for experimental validation. The SANS procedure, which dynamically adjusts negative sample selection based on graph density, modestly improved BioPathNet’s performance in tasks such as gene function prediction, lncRNA–target prediction and SL. However, its impact on drug–disease indication and contraindication prediction was variable, enhancing performance in some cases while proving detrimental in others. Among the four tasks used to evaluate BioPathNet, two (gene function prediction and lncRNA–target prediction) use Pathway Commons as a BRG for message passing. The other two (drug repurposing and SL prediction) leverage unused edges (excluding those in supervised training) as the BRG. Our ablation study confirms the BRG’s impact across all tasks. For gene function prediction, the Pathway Commons-derived BRG significantly boosted performance (16–24% improvement in MRR, Hits@1-3-10), while its effect on lncRNA–target prediction was more modest (1.5–6.3%), suggesting that a tailored BRG, perhaps incorporating a chromatin graph to account for lncRNA nuclear regulatory functions, could further enhance results for this task. The ablation of the BRG was especially crucial for the zero-shot drug repurposing task, where its removal severely disrupted PrimeKG’s connectivity, leading to poor performance (although still above random). For SL, other BRG relations did not directly benefit the task, but the additional SL links within the BRG were essential, as demonstrated by full BRG ablation and individual edge-type ablation experiments. Perturbation experiments across the four tasks further revealed that no single relation type within the BRG significantly impacted performance, except for minor effects in a few cases. This suggests that graphs often contain redundancy and noise, underscoring the need for the biomedical KG community to focus on KG denoising techniques and manual curation (for example, filtering SL links in SynLethDB improved performance). In addition, connectivity between the training graph from G1 and the BRG is the most critical factor, with a strong positive correlation between their connectivity and model performance (MRR). BioPathNet’s performance was minimally affected by redundant connections, demonstrating its robustness to noise. This suggests that meaningful paths between head and tail nodes can still be identified using path-embedding methods, even in noisy or progressively sparser graphs. Since other path-based and KGE-based methods lack ablation analyses on edges and relation types, it is difficult to generalize this conclusion, highlighting the need for further baseline evaluations in this area. The advantage of using the BRG in BioPathNet extends beyond improving LP task performance. While NBFNet could theoretically be trained on the full training graph from G1+BRG graph (as attempted here) to achieve comparable or slightly lower performance than BioPathNet, doing so is computationally impractical—even across multiple GPUs—except perhaps for SL, as demonstrated by our runtime and resource consumption analysis. As a result, training NBFNet on G1 alone remains the only viable option. By leveraging the BRG solely for message passing (without evaluating BRG edges in the loss), BioPathNet not only improves or maintains performance but also ensures a feasible training process. This is one of the key advantages of BioPathNet, which ensures not only sustainability and lower infrastructure costs but also scalability, guaranteeing its future use on even larger datasets and evolving biomedical KGs without becoming prohibitive.

When benchmarked against general-purpose baselines, BioPathNet not only outperformed one-hop KGE methods such as TransE, DistMult and RotatE, as expected, but also consistently surpassed more expressive node-embedding models such as R-GCN, RAGAT and HGT across all tasks, with performance gains in MRR ranging from 2% for gene function prediction to 87% for drug repurposing. This underscores the strength and expressiveness of path representation learning over methods that compute node and relation embeddings separately, even when incorporating graph heterogeneity in their representations. However, the superior accuracy and performance of path representation learning in BioPathNet come at the cost of higher computational training time and resource requirements compared with node embedding methods, which are faster to train (for example, HGT is up to 20 times faster, while R-GCN is 3 times faster on the gene function prediction task). However, BioPathNet was the most efficient among path-based baselines, with a per-epoch training time 23 times shorter than that of KR4SL and, on average, 9–80 times lower than that of NBFNet across tasks.

When compared against task-specific baselines across the four different LP tasks, BioPathNet outperformed drug repurposing-specific baselines DREAMwalk and state-of-the-art TxGNN across all five zero-shot disease splits (adrenal gland, anaemia, cardiovascular, cell proliferation and mental health), with an average AUPRC increase of 60.8% over DREAMwalk and 23.2% increase over TxGNN. In addition, BioPathNet achieved higher Recall@k values, prioritizing known treatments better in the top predictions. Specifically, BioPathNet recovered 61.9% of known treatments at *k* = 20, compared with 53.9% with TxGNN. This is especially valuable in biology, as BioPathNet’s enhanced prioritization reduces the number of predictions requiring verification for biological plausibility during hypothesis generation or experimental validation. It must be noted that performance on the drug indication task varies markedly across disease splits, with MRR ranging from >0.6–0.8 for cell proliferation and adrenal gland to 0.23–0.36 for cardiovascular and mental health. Similar trends were reported in the original TxGNN paper^54^, suggesting that this variability reflects intrinsic properties of the disease splits, such as disease complexity and the amount of available knowledge (for example, known drugs or molecular mechanisms in the graph), rather than differences in modelling approaches. Supporting this, we observed a strong Pearson correlation (*r* = 0.81) between task performance and the average connectivity of drugs in the G1 graph (Supplementary Fig. 8), indicating that performance is closely tied to how well subdiseases are linked to drugs known from other indications. While in predicting contraindications, BioPathNet largely outperformed DREAMwalk which largely fails on this task, it showed comparable or slightly worse results compared with TxGNN (BioPathNet MRR of 0.53 vs 0.56 for TxGNN) and higher performance variance.

BioPathNet outperforms both general and task-specific GNN methods due to its unique encoding approach, which captures path representations rather than individual entity embeddings. Its biomedical KG-specific design and enhanced scalability improve upon its NBFNet baseline (Fig. 4b). When compared to node embedding methods such as DREAMwalk and TxGNN, additional advantages and limitations emerge. BioPathNet’s superior prediction accuracy comes at the cost of longer training time. DREAMwalk, while the least accurate for drug indication prediction, is extremely fast to train, making it valuable for practical applications. TxGNN, although slower than DREAMwalk due to its pre-training phase, enhances disease nodes with limited molecular characterization using a gated auxiliary embedding. In contrast, BioPathNet leverages non-drug–disease triplets for message passing within a BRG but does not require separate pre-training and fine-tuning, offering greater flexibility across different regulatory graphs. However, its higher variance compared with TxGNN may result from the absence of pre-training, which helps stabilize predictions.

Path-embedding methods such as BioPathNet enhance representations with multihop relationships and offer greater interpretability than node embeddings by tracing and visualizing paths as well as influent nodes, which aids in verifying predictions and hypothesis generation. Incorporating a BRG further improves path expressiveness and interpretability, revealing crucial paths and validating predictions, such as in the case of Alzheimer’s disease. Node-embedding methods, although easy to understand (via *t*-distributed stochastic neighbor embedding or uniform manifold approximation and projections), lack intrinsic interpretability, requiring post hoc frameworks such as TxGNN. Path embeddings capture richer context and can be interpreted more easily, but are more abstract and less intuitive for downstream tasks.

We demonstrate the versatility of BioPathNet in addressing diverse problems across various KGs and in SL, also in the inductive setting. However, the task of inferring lncRNA–target regulatory relationship is the hardest for all methods, including BioPathNet, maybe due to the quality and uncertainty of both training data and BRG. Here we attempt this task making use of an lncRNA–target-specific KG coupled with a BRG. Despite a test MRR of 0.19 (lower than for tasks such as drug repurposing or SL) indicating that BioPathNet could probably benefit from more training data, BioPathNet still strongly learns the structure of the lncRNA–targets regulatory graph by leveraging multiple paths in the sparse graph and by that, compensating for the lack of direct connections.

As an additional remark, under the open-world assumption, evaluating model performance on incomplete KGs may not fully reflect their capabilities. Metrics, such as MRR, can degrade in scenarios with high incompleteness, where missing links correlate with specific entities, as discussed in ref. ^100^. Biomedical KGs exhibit uneven gaps in knowledge distribution, influenced by factors such as prevalence and complexity, potentially underestimating BioPathNet’s performance, particularly in tasks such as lncRNA–target prediction.

Limitations of BioPathNet include potential biases in the training data. For example, while BioPathNet successfully retrieved almost all known treatments for AD in its top predictions, it missed FDA-approved drugs such as pramiracetam, memantine and lecanemab, which were not listed in the PrimeKG database and lacked disease indications. As a result, the model could not learn their connections and did not identify them as potential treatments. Predictions for known symptom-treating drugs focused on neuropsychiatric-related diseases, but emphasizing molecular interactions could uncover more disease-modifying treatments. Future improvements might involve excluding message passing over dominant relations such as indications and prioritizing molecular interactions to elucidate mechanisms underlying less-understood diseases such as Alzheimer’s. Lastly, BioPathNet uses a distinct BRG for each task, which may introduce biases based on the chosen BRG. While we assess the impact of node degree, relation type removal and random edge perturbations, task performance may highly depend on BRG selection. Although an ideal solution would be a unified, denoised biomedical KG for all tasks, the complexity of biomedical KGs makes this challenging. Preliminary results suggest that task-specific BRGs yield better performance. For instance, replacing the SL BRG with the gene function BRG led to comparable or lower MRR, while substituting the PrimeKG portion with Pathway Commons dramatically reduced drug repurposing performance due to missing drug–gene connections. As highlighted in ref. ^52^, task-specific KGs are crucial for the success of certain tasks, and future work should explore in more detail the extent to which different tasks benefit from distinct previous knowledge.

In summary, BioPathNet is a path-embedding method for link prediction on biological KGs, achieving state-of-the-art performance in tasks such as gene function prediction, drug indication, synthetic lethality and mRNA–lncRNA interactions. Its interpretability framework highlights key prediction paths, improving biological plausibility and bias detection. Future directions include refining and denoising biomedical KGs, leveraging condition-specific knowledge and integrating node features. Ultimately, BioPathNet could serve as a foundation for predictive models in biomedical KGs, accelerating hypothesis generation across biology and medicine.

## Methods

### KG completion

A knowledge graph KG = {(*u*, *r*, *v*)}_*u*,*v*∈*E*,*r*∈*R*_ is a heterogeneous directed graph where nodes *E* represent entities, relations *R* represent edges, and triplets (*u*, *r*, *v*) define connections, with *u* (head) linking to *v* (tail) via *r* (relation). The graph is considered heterogeneous because different entities may have different types, for example, a node representing a gene versus a node representing a disease. The graph is directed because (*u*, *r*, *v*) being in the KG does not imply (*v*, *r*, *u*) is contained as well. KG completion involves predicting the missing links, that is, tail prediction (*u*, *r*, ?), predicting the tail entity given the head entity and the relationship^101^.

### NBFNet

**Fig. 7:**
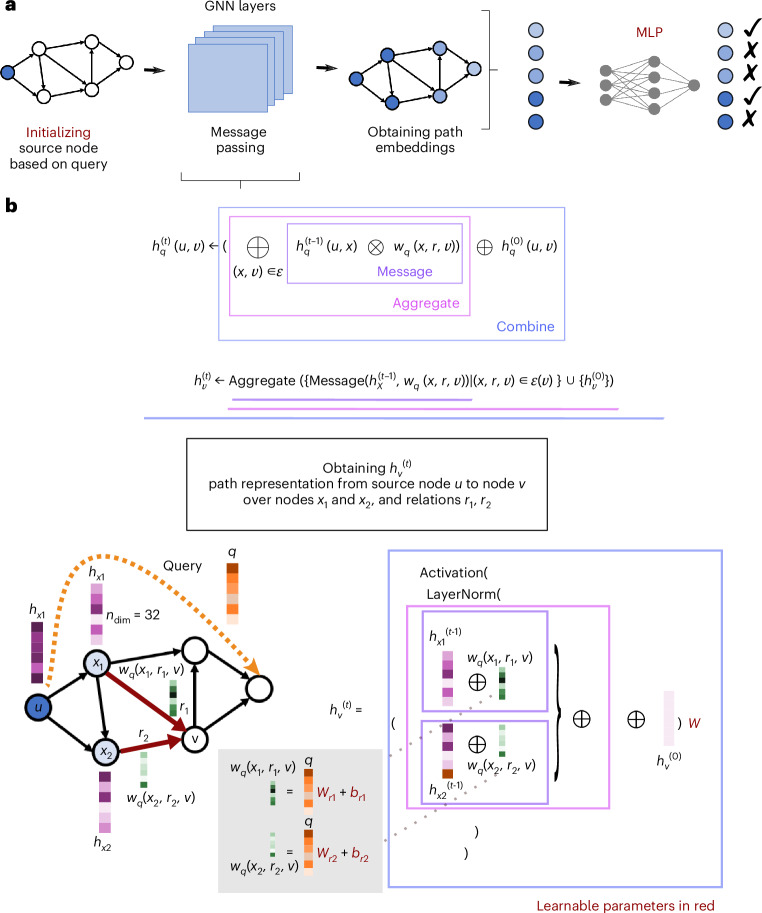
Model architecture. **a**, Overview: to predict a query relation between a source node and other nodes, the source node is initialized with the query embedding, while others are set to zero (boundary condition). Message passing is performed over *t* GNN layers (for example, *t* = 4), producing embeddings that represent paths from the source node. These embeddings are then fed into a multilayer perceptron (MLP) to predict the query relation. **b**, Message passing (for example, using the DistMult function (multiplication) for messages and summation for aggregation): the embedding of node *v* (relative to source node *u*) is updated on the basis of incoming messages from neighbours *x*_1_, *x*_2_ and the boundary condition. Messages are computed by multiplying neighbour embeddings (${h}_{{x}_{1}}^{(t-1)}$, ${h}_{{x}_{2}}^{(t-1)}$) with relation embeddings (*w*_*q*_(*x*_1_, *r*_1_, *v*), *w*_*q*_(*x*_2_, *r*_2_, *v*)) (purple), aggregated via summation (pink) and combined with ${h}_{v}^{(0)}$ through a linear layer, layer norm and activation function (blue). Relation embeddings (*w*_*q*_(*x*_1_, *r*_1_, *v*), *w*_*q*_(*x*_2_, *r*_2_, *v*)) can be learned dynamically dependently on the query *q* as *W*_*r*_*q* + *b*_*r*_.

BioPathNet is a path-representation learning-based method for graph completion built upon the NBFNet framework^56^, which performs link prediction (LP) by learning representations for each path from the query entity *u* to potential tail entities *v*. More specifically, in NBFNet, the path formulation is represented by a generalized sum of path representations between *u* and *v* (Fig. 7a,b and neural Bellman–Ford network (NBFNet) in Extended Methods).

NBFNet’s learning framework relies on the generalized Bellman–Ford algorithm for path representation and its neural abstraction.

#### Generalized Bellmann–Ford path representation

To achieve a scalable path formulation, NBFNet utilizes a generalized version of the Bellman–Ford dynamic programming algorithm^102^.

This generalization transforms the original Bellman–Ford algorithm for shortest path calculation into a versatile framework that simultaneously computes pair representations *h*_*q*_(*u*, *v*) for a given entity *u*, query relation *q*, and all entities *v* in a graph.

1 $${h}_{q}^{(0)}(u,v)\leftarrow {{\mathbb{1}}}_{q}(u=v)$$

2 $${h}_{q}^{(t)}(u,v)\leftarrow \left({\bigoplus }_{(x,v)\in {\mathcal{E}}}{h}_{q}^{(t-1)}(u,x)\otimes {w}_{q}(x,r,v)\right)\oplus {h}_{q}^{(0)}(u,v)$$

In this formulation, the first equation initializes the boundary condition on the source node (equation (1)), representing the shortest path between *u* and *v* at the start. If the head and tail nodes coincide (*u* = *v*), the boundary condition is set to the generalized ${\mathbb{1}}$, which corresponds to 0 in the shortest path context (that is, the shortest distance between a node and itself is zero) and to *∞* in the case *u* ≠ *v*. Equation (2) describes the Bellman–Ford iteration, updating the shortest path distance between *u* and *v*. In each iteration, the representation from the previous layer (*t* − 1) is multiplied by the transition edge representation *w*_*q*_ to obtain the new representation *h*_*q*_(*u*, *v*). The algorithm propagates the boundary condition from the source node to its neighbours. It is important to note that there is a distinction between query relation *q* and the relation *r* in the graph. The query relation *q* is used to initialize the source node (boundary condition), while the transition edge representations *w*_*q*_(*x*, *r*, *v*) are obtained by the multiplication of relation *r* in the graph. Both embeddings are learned. Using the distributive properties of multiplication, all prefixes are computed simultaneously. This iterative process continues, assessing potential target nodes, until all paths from the source to the tail node are covered after *t* iterations, where *t* is the path length (Fig. 7b). For a more detailed description, see Extended Methods in Supplementary Information.

#### Neural formulation

By abstracting the boundary condition in equation (1) to an indicator function, the multiplication operator in equation (2) to a message passing formulation, and the summation operator to a general aggregation function, NBFNet extends the generalized path formulation of the Bellman–Ford algorithm into a graph neural network framework.

3 $${h}_{v}^{(0)}\leftarrow \mathrm{Indicator}(u,v,q)$$

4 $${h}_{v}^{(t)}\leftarrow \mathrm{Aggregate}(\{\mathrm{Message}({h}_{x}^{(t-1)},{w}_{q}(x,r,v))| (x,r,v)\in {\mathcal{E}}(v)\}\cup \{{h}_{v}^{(0)}\})$$

For the indicator function, NBFNet learns the query relation embedding *q* and assigns *q* to node *v* if *v* equals the source node *u*. Message passing uses the DistMult^32^ relational operator (multiplication) for messages and summation for aggregation over *t* GNN layers, generating path embeddings from the source node. Each node embedding *h*_*v*_ (always relative to source *u*) is updated by multiplying neighbour embeddings with relation embeddings, aggregating via permutation-invariant functions (for example, summation or principal neighbourhood aggregation), and combining with *h*_*v*_(0) through a linear layer, normalization layer (LayerNorm) and activation sigmoid function. Relation embeddings can be dynamically learned on the basis of the query *q* (Fig. 7b). LayerNorm standardizes node embeddings across features by normalizing activations, and by that, acts as a regularizer by reducing sensitivity to small variations in node features, helping prevent overfitting.

Unlike typical GNNs, which compute pair representations as independent node embeddings *h*(*u*) and *h*(*v*), NBFNet conditions each node’s representation *h*_*q*_(*u*) and *h*_*q*_(*v*) on the source node and query relation *q*. The resulting pair representation *h*_*q*_(*u*, *v*) is then used for LP, predicting the tail entity *v* given the head entity *u* and relation *q*. This is formulated as the conditional likelihood of the tail entity *v* as:

5 $$p(v| u,q)=\sigma (\,f({h}_{q}(u,v)))$$

where *σ*(_) is the sigmoid function and *f*(_) is a feed-forward neural network. Practically, the model weights are learned using the training edge distribution (Fig. 7a), and the trained model is then applied to the test edge distribution to infer new edges.

### BioPathNet: biomedical KG completion

Considering the unique characteristics of biological KGs, we introduce BioPathNet, a graph neural network framework based on NBFNet^56^. BioPathNet is designed to predict missing links in biomedical KGs.

#### Path representation in BioPathNet

As a path-based reasoning method, path representations *h*_*q*_(*u*, *v*) in BioPathNet are learned starting at a source node *u* to all potential target nodes *v* on the basis of the relations *r* along the path, following the NBFNet parametrization (equations (3) and (4)) but with important enhancements that make BioPathNet more suited for biological KGs. We improve NBFNet in two key ways: first, by refining its negative sampling strategy to consider entity-type information, and second, by introducing a BRG, a message-passing-only graph that enhances efficiency and learning in LP tasks. These design choices enable BioPathNet to effectively handle diverse biomedical KGs.

#### Negative sampling in BioPathNet

During training, for each positive triplet (*u*, *r*, *v*), *n* negative samples $(u,r,{v}^{{\prime} })$ are generated by corrupting one of the entities *v* in the positive sample (that is, substituting the true node tail *v* with another node *v*′) to create a negative triplet. In the basic NBFNet, the ‘self-adversarial negative sampling’ from RotatE^34^ is used, where instead of uniformly sampling *n* negative samples per positive sample, the weights of negative samples are softly redistributed on the basis of the current model embeddings, thereby forcing the model to discriminate on the basis of hard negative samples from positive samples. A parameter called ‘adversarial temperature’ controls the softness of this weight distribution and represents a hyperparameter for BioPathNet.

To further improve the decision boundary and ensure biologically meaningful sampling, BioPathNet implements a NTA negative sampling scheme. This means that when sampling negative *v*′ for training, we consider the node type and sample *v*′ only from the same type as *v*. This approach also reduces the sample space and challenges the learning task by compelling the model to learn a decision boundary with sufficiently difficult negative samples.

In addition to NTA, BioPathNet incorporates SANS, as proposed in ref. ^57^. This method generates negative samples on the basis of graph density, restricting them to the *k*-hop neighbourhood of the head node and using random walks to approximate local neighbourhoods. In our case we used *k* = 2, which is a reasonable choice given that biomedical networks are typically well connected and shallow, with an average diameter of ~4–6. Sampling negatives from similar-density regions can mitigate biases due to local graph structure and improves generalization in sparser graph areas. Negative sampling strategies were tested in ablation experiments.

#### Incorporation of a BRG

Compared to the original NBFNet, BioPathNet incorporates additional entity and relation information, referred to as the BRG or G2, for message passing. Among the four tasks used to evaluate the performance of BioPathNet, gene function prediction and lncRNA–target prediction utilize Pathway Commons, an external database of protein–protein and protein–chemical interactions, as the BRG (see Supplementary Table 2). In contrast, drug repurposing and SL prediction define the BRG using the unused edges of the dataset, excluding relations explicitly used for supervised training, referred to as G1. The incorporation of a BRG provides additional edges (knowledge) that are used solely for message passing, enhancing the prediction of links of interest (Fig. 1c). For example, in predicting the missing link (*u*, *r*, *v*), where (*u*, *r*, *v*) always comes from the target KG G_1_, messages can be passed only along paths in G1, yielding a prediction path such as in Fig. 1d. Alternatively, a BRG G2 can be used to further inform the predictions, leveraging additional knowledge (for example, including relations between type 2 and 3 nodes), as illustrated in Fig. 1e. Consequently, in BioPathNet, equation (4) is modified to always take (*u*, *r*, ?) from G1 but to aggregate and send messages across all edges G1 ∪ G2. In equation (4), the edges $(x,r,z)\in {\mathcal{E}}(v)$ come from G1 ∪ G2 rather than just G1.

#### BioPathNet training and LP

During the training of the model in a supervised setting, the negative log-likelihood is minimized between positive samples (*u*, *r*, *v*) and negative samples $(u,r,{v}^{{\prime} })$, generated according to the sampling scheme outlined above.

6 $${L}_{\mathrm{KG}}=-\log p(u,q,v)-\mathop{\sum }\limits_{i=1}^{n}\frac{1}{n}\,\log (1-p(u,q,{v}_{i}^{{\prime} }))$$

where *n* is the number of negative samples per positive sample and $(u,q,{v}_{i}^{{\prime} })$ is the *i*th negative sample for KGs.

Importantly, the additional edges from the BRG are not used for sampling positive and negative triplets, ensuring that the computation of the loss in equation (6) remains unchanged. After performing LP with the modified message passing scheme, BioPathNet ranks all candidate tail entities according to their likelihood *p*(*v*∣*u*, *q*) to form a true triplet with a given head entity and relation *q* as the query. The same approach is used for the prediction of *v* given *u* and *r*^−1^ for the reverse relation of *r*. It is important to notice that in the transductive setting, the same BRG is used for training and inference, ensuring identical graphs. In contrast, inductive tasks such as SL predictions use separate BRGs, with part reserved for training and another for inference, where the inference graph includes unseen nodes.

### Interpretation of prediction: visualization of most important paths

Leveraging the NBFNet framework, BioPathNet predictions can be directly interpreted through paths. This feature is crucial for biomedical tasks, where understanding the mechanisms behind each prediction is essential. These interpretations highlight the paths that contribute the most to the prediction *p*(*v*∣*u*, *q*). Using local interpretation methods, we approximated the local landscape of BioPathNet with a linear model over the set of all paths, and the importance was then defined by its weight in the linear model, which can then be computed as the partial derivative of the prediction with respect to the path^56^. Formally, the top-*k* path interpretations for *p*(*u*, *q*, *v*) are defined as:

7 $${P}_{1},{P}_{2},\ldots ,{P}_{k}=\mathrm{top}-{k}_{P\in {P}_{uv}}\frac{\partial p(u,q,v)}{\partial P}$$

While directly computing the importance of all paths is intractable, NBFNet approximates them with edge importance. Specifically, the importance of each path is approximated by the sum of the importance of edges in that path, and therefore intuitively, the top-*k* path interpretations are equivalent to the top-*k* longest paths on the edge importance graph.

For the visualization plot, we considered the top 10 most important paths ranked by gradient, with the edge width reflecting the number of times an edge appears in paths. Furthermore, the most important path is highlighted in red. In summary, our interpretability allows predictions to be assessed by their biological plausibility for hypothesis generation or validation in the laboratory.

### BioPathNet training on four different biomedical tasks

#### Gene function prediction task: identifying gene–pathway associations

For this task, we used the KG from the KEGG database (G1), extracted from ConsensuPathDB, to train the model on gene (G)–pathway (P) interactions in transductive setting. In addition, we utilized a BRG containing regulatory relationships between gene–gene (G–G), gene–chemical (G–C) and chemical–chemical (C–C) interactions obtained from Pathway Commons (G2)^59,60^. These interactions were represented as triplets in the format (node1, relationType, node2), such as (BABAM1, interacts-with, PSMD14) (Supplementary Tables 1 and 2). During data preprocessing of G1, we removed KEGG pathways with fewer than 10 annotations to genes and retained only the biggest connected component of the BRG, thereby removing 11 nodes present in components of only 2–3 nodes. We trained BioPathNet using 70% of the P–G triplets from the G1, which were randomly split, with and without incorporating the underlying BRG as a message-passing graph. We used 10% of the G1 P–G triplets for validation, and the remaining 20% of G1 were reserved for testing. During training, training triplets were used as the message-passing graph (that is, training graph), while removing the specific triplets of the batch for learning path representations. This graph was also used during validation and testing, in addition to a potential G2/BRG. Hyperparameters, including adversarial temperature and number of negatives per positive sample, were optimized on the basis of the validation MRR, resulting in an optimal set of parameters for downstream analysis (Supplementary Table 16).

#### Drug repurposing task: predicting unknown drug–disease associations

This task is based on PrimeKG^53^ (Supplementary Fig. 1a), splitting the data into training, validation and test sets (Supplementary Table 3). For training, validation and testing of BioPathNet, we used the same data and data splits as defined by TxGNN^54^, reflecting a zero-shot prediction scenario at a proportion of 0.83:0.12:0.05. By using the TxGNN code (https://github.com/mims-harvard/TxGNN from 12 Apr 2023, commit ‘1000aac’), the splits were created by removing all indication and contraindication triplets for a disease area from training, along with 95% of connections to biomedical entities (for example, proteins, phenotypes)^54^, simulating minimal molecular characterization and no therapeutic knowledge. While TxGNN constructs reverse edges, we removed them beforehand, as BioPathNet inherently adds reverse triplets during reasoning. Five zero-shot disease areas were used: adrenal gland disorders, anaemia, cardiovascular diseases, cell proliferation diseases (for example, various cancers) and mental health conditions (Supplementary Table 3). BioPathNet was trained in a supervised manner using only drug–disease edges (indication, contraindication) in G1, while the rest of PrimeKG served as the BRG (G2) for message passing. After removing reverse relations, 5.7 million directed edges were used for message passing per prediction setting, consisting of protein–protein or disease–disease relations. The training and validation sets averaged 33,000 and 4,000 edges, respectively, across five disease areas (Supplementary Table 3).

We further created our custom data split with TxGNN’s ‘disease_eval’ code to evaluate performance in predicting drugs for the neurodegenerative disorder Alzheimer’s disease. Drugs that were associated with various AD diseases were moved to the test set (Supplementary Table 17). All models were trained for 10 epochs, employing an early stopping mechanism that retained the best model based on validation set performance (MRR, see below). The final hyperparameters, including the number of negatives per positive sample for all five disease area splits, are reported in Supplementary Table 12. All experiments were repeated for five different data-split seeds, using the exact seeds employed by TxGNN to ensure a fair comparison. Each data split seed resulted in slightly different training and validation sets for each disease area due to the random removal of edges to simulate the zero-shot scenario for the disease under study every time.

#### SL prediction task: identifying unknown SL gene pairs

For this task, we used the SynLethDB-v.2.0 (ref. ^103^) data as a KG (Supplementary Table 18). In transductive setting, BioPathNet was trained on pre-processed data, that is, the SL gene pairs extracted from SynLethDB-v.2.0 (G1), as provided by KR4SL^55^ and downloaded from their GitHub repository (https://github.com/JieZheng-ShanghaiTech/KR4SL from 8 Dec 2023, commit ‘61b5c84’). In detail, the SL gene pairs from SynLethDB-v.2.0 for humans were randomly split into train, validation and test triplets at a ratio of 7:1:2, following the data split of KR4SL (Supplementary Tables 19 and 20). The pre-processed data were modified to fit the BioPathNet format by removing the reverse edges introduced by KR4SL for SL gene pairs (following the same preprocessing scheme as in the drug repurposing task), as reverse edges are implicitly added by BioPathNet by default.

On top of the SL pairs, SynLethDB-v.2.0 constructs a KG including relations between gene entities, pathways and three types of GO term: biological processes (BP), molecular functions (MF) and cellular components (CC) augmented using OntoProtein^50^. While SL pairs were used for supervised training (G1), the rest of the KG (G2), augmented by additional SL pairs, was used as BRG for message passing only, following the same scheme from previous tasks (Supplementary Table 18). Hyperparameters, including the number of negatives per positive sample, were optimized on the basis of the validation MRR, resulting in an optimal set of parameters for downstream analysis (Supplementary Table 21). In transductive setting, BioPathNet was trained and evaluated on SynLethDB SL pairs across confidence thresholds from 0.1 to 0.8. For final training, pairs with confidence thresholds below 0.3 were excluded, removing most if not all computationally predicted interactions (Supplementary Fig. 2a). The final dataset included 8,770 training, 3,172 validation, 6,254 test, and 13,161 known SL pairs in the BRG (Supplementary Table 18). The filtered set is referred to as ‘thresholded data,’ while the original is ‘unthresholded data’, which includes all SL pairs from SynLethDB-v.2.0 without filtering by confidence score. For both thresholded and unthresholded data, we also trained BioPathNet in the inductive setting, using the data split from the KR4SL study, ensuring that training and testing graphs’ node sets were not overlapping (Supplementary Table 22). All experiments were run for five random seeds and the optimal hyperparameters are reported in Supplementary Table 21.

Since SL relationships are symmetric between genes, the final score for a gene *v* to be an SL partner of gene *u* was computed by considering both the feed-forward neural network transformed representation of tail node *v* given head node *u* and the SL relation, *f*(*h*_*q*_(*u*, *v*)), as well as the transformed symmetric representation, $f({h}_{{q}^{-1}}(u,v))$. Thus, the final SL score for a gene *v* to be an SL partner of gene *u* from BioPathNet is reported as:

8 $$p(v| u)=\sigma \left(\frac{f({h}_{q}(u,v))+f({h}_{{q}^{-1}}(u,v))}{2}\right)$$

#### LncRNA–target prediction task: identifying unknown lncRNA–gene relationships

For this task, we used the LncTarD 2.0 database^64^, where interactions are categorized into seven mechanisms of lncRNA–target regulation: ‘ceRNA or sponge’, ‘chromatin looping’, ‘epigenetic regulation’, ‘expression association’, ‘interact with mRNA’, ‘interact with protein’ and ‘transcriptional regulation’. Missing gene information (for example, Ensembl IDs, gene names) was resolved using Gencode and HGNC mapping. Due to the scarcity of chromatin looping interactions (12 pairs), we relabelled them as ‘transcriptional regulation’ after a manual literature review, consolidating interactions into six distinct types.

On top of the lncRNA–target interactions from LncTarD 2.0 KG, we added the BRG derived from Pathway Commons (G2), the same BRG used for the gene function prediction task. As there is no direct link between small molecules and lncRNA, we only used PPI, discarding other types of relation (Supplementary Tables 23 and 24). To enable NTA negative sampling, node entities in G1 were labelled with six different categories: ‘lncRNA‘, ‘mRNA’, ‘microRNA’, ‘transcription factor’, ‘protein’ and ‘protein_ppi’ (this last one to specifically identify nodes from PPI interactions from the BRG, whose edges were only used for message passing and not for supervised training). The optimal parameters of BioPathNet in this setting, determined through the MRR on the validation set, are reported in Supplementary Table 25.

### Comparison with baselines

We compared BioPathNet against several general-purpose baselines, namely: the graph neural network-based R-GCN, which extends GCNs for multirelational data by using relation-specific weight matrices to aggregate neighbour information while preserving relational semantics^104^; HGT, a GNN model which captures graph heterogeneity with type-dependent parameters, preserving distinct representations while integrating high-order neighbour information^44^; RAGAT, an embedding method which introduces node- and edge-type specific message passing functions on a heterogeneous graph^43^; and the original NBFNet^56^.

For gene function and lncRNA–target prediction, we compared BioPathNet to BIONIC, a GCN-based biological network integration framework that learns gene embeddings in an unsupervised or semi-supervised manner^42^. BIONIC generates separate gene embeddings from multiple networks and integrates them into a unified representation for downstream tasks. Here, it was trained to predict functional classes for genes and multiple targets for each lncRNA (referred to as lncBIONIC in the latter case).

For the drug repurposing prediction task, we compared BioPathNet to TxGNN^54^ and DREAMwalk^42^. TxGNN is a state-of-the-art model for predicting drug–disease relationships in zero-shot scenarios where minimal previous information or treatment history is available^53^. Leveraging PrimeKG^53^, a comprehensive biomedical KG, TxGNN uses R-GCNs to learn embeddings of drugs and diseases, capturing complex interactions by mapping them into a shared latent space. DREAMwalk constructs a fixed biomedical KG including a drug–drug and disease–disease similarity network, along with links connecting drugs and diseases to a PPI network. It generates embeddings via a heterogeneous Skip-gram model and classifies drug–disease associations with XGBoost. Since the original graph (built from the ATC classification and from the Medical Subject Heading (MeSH)) was unavailable, we reconstructed it in two ways: (1) DREAMwalk_IC, mapping DrugBank and MONDO (MeSH-inclusive) hierarchies used in PrimeKG to compute drug–drug and disease–disease semantic similarity, and (2) DREAMwalk_OC, directly extracting connections from PrimeKG.

For the SL pair prediction task, BioPathNet was benchmarked against KR4SL, a path-representation learning GNN-based method designed specifically for the explainable prediction of SL gene pairs in cancer^55^ in both transductive and inductive setting.

For the lncRNA–target prediction task, BioPathNet was benchmarked against BIONIC, as described earlier, and DeepLGP, a task-specific baseline. DeepLGP^65^ prioritizes lncRNA target genes by integrating lncRNA and gene features (such as genomic location, expression and miRNA interactions) with graph features extracted via a GCN encoder.

All baseline methods were retrained using optimal parameters, either selected on the basis of validation MRR when possible, or set to the default values recommended by the original authors, to ensure a fair comparison. Detailed descriptions of each baseline and the specifics of the final model parameters are provided in ‘Baseline methods used for comparison’ in Extended Methods.

### Model evaluation

Various metrics were used to evaluate BioPathNet on a held-out test set (part of the G1) across different tasks and compare its performance with baseline methods. For all tasks, methods were evaluated on the basis of: mean rank (MR), the average rank of the true positive among all predicted candidates; mean reciprocal rank (MRR), the average of the reciprocal ranks of the first relevant item; and Hits@k, the proportion of true positives ranked within the top-*k* predictions. Values for these metrics range [0, 1], and the larger the value, the better the model (for an extensive explanation of these metrics, see Extended Methods in Supplementary Information). The random MRR represents the expected mean reciprocal rank if predictions were made by randomly guessing, meaning the correct answer is equally likely to appear in any possible ranking position among all negatives, with the final value depending on the number of available negative candidates for each positive sample.

While node embedding models the conditional probability of predicting the tail entity *v* given the head entity *u* and relation *r*, evaluating the joint probability of *u*, *v* and *r* may be more comprehensive. To ensure consistency with TxGNN in drug prediction, we also used AUPRC to summarize precision and recall across thresholds, along with specificity and F1 score at a 0.5 threshold, using TxGNN’s evaluation code. This approach assesses the performance of each disease node. For details on computing AUPRC in the comparison between BioPathNet and TxGNN, see Extended Methods in Supplementary Information.

For the SL prediction task, we compared the seed-wise performance of our model with the performance of KR4SL using metrics inherent to the KR4SL framework’s code, specifically NDCG@k, Recall@k and Precision@k (see ‘Model evaluation’ in Extended Methods). Moreover, we computed MRR for both BioPathNet and KR4SL by first calculating MRR for each query gene and then averaging gene-wise MRRs over all query genes. For the SL task, we ensured that predicted triplets (SL relationships) already present in the BRG were excluded from the performance evaluation, guaranteeing a reliable assessment of generalization performance.

### Reporting summary

Further information on research design is available in the Nature Portfolio Reporting Summary linked to this article.

## Supplementary information

Supplementary Information Extended Methods and Supplementary Tables 1–25 and Figs. 1–8.

Reporting Summary

Peer Review file

## Data Availability

Gene function prediction data are available from Pathway Commons and ConsensusDB (see Methods for details). For drug–disease prediction, PrimeKG data and splits were downloaded via TxGNN. SynLethDB data, processed by KR4SL, were obtained from their GitHub, and lncRNA target prediction data from LncTarD 2.0. Further preprocessing was done to fit BioPathNet’s data format, and all scripts can be found on GitHub at https://github.com/emyyue/BioPathNet (ref. ^105^). A bash script to download all necessary data from the corresponding locations is also provided on GitHub at https://github.com/emyyue/BioPathNet/blob/main/data/download.sh (ref. ^106^).

## Code availability

The BioPathNet model and all code necessary for reproducing our results are publicly available via GitHub at https://github.com/emyyue/BioPathNet (ref. ^105^).
