# Supplementary material for: Enhancing link prediction in biomedical knowledge graphs with BioPathNet
Source: Nat Biomed Eng. 2026 Jan 20;10(8):1747–69. doi: 10.1038/s41551-025-01598-z (PMC13472943; doi:10.1038/s41551-025-01598-z)
Supplement: Supplementary file 1 [file 41551_2025_1598_MOESM1_ESM.pdf]

nature biomedical engineering

Supplementary information

https://doi.org/10.1038/s41551-025-01598-z

Enhancing link prediction in biomedical knowledge graphs with BioPathNet

In the format provided by the authors and unedited

Contents

1 Extended Methods **2**
1.1 Neural Bellman-Ford Network (NBFNet) . . . 2
1.2 Gene function prediction task . . . 3
1.3 Drug repurposing task . . . 4
1.4 Synthetic lethality gene pair prediction task . . . 4
1.5 LncRNA-target prediction task . . . 4
1.6 Baseline methods used for comparison . . . 4
1.7 Model Evaluation . . . 7

2 Supplementary Tables **10**
2.1 Supplementary Table 1 . . . 10
2.2 Supplementary Table 2 . . . 10
2.3 Supplementary Table 3 . . . 10
2.4 Supplementary Table 4 . . . 11
2.5 Supplementary Table 5 . . . 11
2.6 Supplementary Table 6 . . . 12
2.7 Supplementary Table 7 . . . 13
2.8 Supplementary Table 8 . . . 13
2.9 Supplementary Table 9 . . . 13
2.10 Supplementary Table 10 . . . 14
2.11 Supplementary Table 11 . . . 15
2.12 Supplementary Table 12 . . . 15
2.13 Supplementary Table 13 . . . 16
2.14 Supplementary Table 14 . . . 16
2.15 Supplementary Table 15 . . . 16
2.16 Supplementary Table 16 . . . 17
2.17 Supplementary Table 17 . . . 17
2.18 Supplementary Table 18 . . . 18
2.19 Supplementary Table 19 . . . 19
2.20 Supplementary Table 20 . . . 20
2.21 Supplementary Table 21 . . . 20
2.22 Supplementary Table 22 . . . 21
2.23 Supplementary Table 23 . . . 22
2.24 Supplementary Table 24 . . . 22
2.25 Supplementary Table 25 . . . 22

3 Supplementary Figures **23**
3.1 Supplementary Figure 1 . . . 23
3.2 Supplementary Figure 2 . . . 24
3.3 Supplementary Figure 3 . . . 25
3.4 Supplementary Figure 4 . . . 26
3.5 Supplementary Figure 5 . . . 27
3.6 Supplementary Figure 6 . . . 28
3.7 Supplementary Figure 7 . . . 29
3.8 Supplementary Figure 8 . . . 30

1 Extended Methods

1.1 Neural Bellman-Ford Network (NBFNet)

Our newly developed BioPathNet is a path-representation learning-based method for graph completion built upon the NBFNet framework. Below, we describe the foundations of NBFNet to provide an understanding of how BioPathNet operates.

Unlike node embedding methods or node GNN encoders that infer links between entities in a KG by learning node representations in an embedding space, NBFNet is a general graph neural network framework that performs link prediction by learning representations for each path from the query entity to potential tail entities. This is done by considering the relations along each possible path between the query entity and the potential tail entity. Given a KG, NBFNet learns to predict the tail node for a query $(u, q, ?)$. It does this by learning a node pair representation $h_q(u, v)$ for nodes u and v, which captures all the possible paths between u and v conditioned on q. In NBFNet, the path formulation is represented by a generalized sum of path representations between u and v, denoted by $h_q(P)$, with a commutative summation operator $\bigoplus$:

$$h_q(u, v) = h_q(P_1) \oplus h_q(P_2) \oplus ...... \oplus h_q(P_M) \tag{1}$$

where M denotes the possible number of paths between u and v. Each path representation $h_q(P)$ is defined as the generalized product of the edges (or edges representations) belonging to that path, with the multiplication operator $\bigotimes$.

$$h_q(P) = w_q(e_1) \otimes w_q(e_1) \otimes .... \otimes w_q(e_p) \tag{2}$$

where p is number of edges $e_1, ..., e_p$ belonging to path P. In compact form, the path formulation can be written as:

$$h_q(u, v) = \bigoplus_{p \in P_{u,v}} \bigotimes_{e \in P} w_q(e) \tag{3}$$

This, in practice, means that we compute the representation between nodes u and v under query relation q by considering every path between the source node u and the target node v. Each path is represented by the product of edge representations w_q along that path, and we sum these products to obtain the final representation.

Two key factors contribute to NBFNet's scalability for large graphs and its effectiveness in learning tasks: the use of the generalized Bellman-Ford dynamic programming framework for path representation and the abstraction of this process into a neural formulation.

Generalized Bellman-Ford path representation The approach above, which predicts a link between a head and tail node by enumerating all possible paths and summing their contributions, is not scalable. As the network grows, the number of possible paths between the head and tail nodes increases exponentially, making this method impractical for larger networks. To achieve a scalable path formulation, NBFNet utilizes the Bellman-Ford dynamic programming algorithm, which efficiently finds the shortest path from a single source node to all other nodes in a weighted graph through a recursive process [1]:

$$d[v] = min(d[u] + w(u, v)) \tag{4}$$

By extending this equation to generalize the addition operator to any summation operator $\bigoplus$ and the minimum operator to any multiplication operator $\bigotimes$, we derive the generalized Bellman-Ford algorithm [2]. This generalization transforms the original Bellman-Ford algorithm for shortest path calculation into a versatile framework that simultaneously computes pair representations $h_q(u, v)$ for a given entity u, query relation q, and all vertices v in a graph. This approach reduces the computational cost to polynomial time relative to the number of nodes and edges in the graph. The formulation of the generalized Bellman-Ford algorithm is as follows:

$$h_q^{(0)}(u,v) \leftarrow \mathbb{1}_q(u=v) \tag{5}$$

$$h_q^{(t)}(u,v) \leftarrow \Big(\bigoplus_{(x,v)\in\mathcal{E}} h_q^{(t-1)}(u,x) \otimes w_q(x,r,v)\Big) \oplus h_q^{(0)}(u,v) \tag{6}$$

It first initializes the boundary condition on the source node (equation 5), representing the shortest path between u and v at the start. If the head and tail nodes coincide ($u = v$), the boundary condition is set to the generalized $\mathbb{1}$, which corresponds to 0 in the shortest path context (i.e., the shortest distance between a node and itself is zero) and to ∞ in the case $u \neq v$. Equation 6 describes the Bellman-Ford iteration, updating the shortest path distance between u and v. In each iteration, the representation from the previous layer $(t-1)$ is multiplied by the transition edge representation w_q to obtain the new representation $h_q(u,v)$. Here, W_q is a function of the relation between u and v. The boundary condition is added at each step to ensure accurate path formulation. The algorithm starts by propagating the boundary condition from the source node to its neighbors. Thanks to the distributive properties of the multiplication operator, all prefixes sharing this propagation are computed simultaneously. This iterative process continues, assessing potential target nodes, until all paths from the source node to the tail node are covered after t iterations, where t represents the path length.

Neural formulation The generalized summation and multiplication operators are handcrafted. By abstracting the boundary condition in equation 5 to an indicator function, the multiplication operator in equation 6 to a message passing formulation, and the summation operator to a general aggregation function, NBFNet extends the generalized path formulation of the Bellman-Ford algorithm into a neural network framework. In this formulation, neural network functions parametrize the boundary condition, multiplication, and summation functions, allowing a generalized GNN framework to learn these three neural functions effectively.

$$h_v^{(0)} \leftarrow INDICATOR(u,v,q) \tag{7}$$

$$h_v^{(t)} \leftarrow AGGREGATE(\{MESSAGE(h_x^{(t-1)}, w_q(x,r,v)) | (x,r,v) \in \mathcal{E}(v)\} \cup \{h_v^{(0)}\}) \tag{8}$$

For the indicator function, NBFNet learns the embedding of the query relation q and assigns q to node v if v equals the source node u. For message passing, NBFNet borrows relational operators from KG embeddings, such as translation from TransE, multiplication from DistMult, and rotation from RotatE. Aggregation functions are implemented using permutation-invariant functions from GNN literature, including sum, mean, max, and principal neighborhood aggregation (PNA). Traditionally, edge representations are defined as transition probabilities or lengths. However, since an edge's contribution varies with the query relation, NBFNet parameterizes edge representations as a linear function of the query relation [3]. NBFNet can be interpreted as a novel GNN framework for learning pair representations. Unlike common GNN frameworks, which compute pair representations as two independent node representations $h_q(u)$ and $h_q(u)$, in NBFNet, each node learns a representation conditioned on the source node. The learned pair representation $h_q(u,v)$ is then used to solve the link prediction problem, i.e., predicting the tail entity v given the head entity u and relation q. This is formulated as the conditional likelihood of the tail entity v as:

$$p(v|u,q) = \sigma(f(h_q(u,v))) \tag{9}$$

where $\sigma(\_)$ is the sigmoid function and $f(\_)$ is a feed-forward neural network.

1.2 Gene function prediction task

The following hyperparameters of BioPathNet were optimized for: adversarial temperature (corresponding to the temperature in self-adversarial negative sampling) $\{0.5, 1.0, 2.0\}$; negative samples: $\{32, 64\}$; aggregation function: sum, PNA (Principal Neighborhood Aggregator [4]; number of hidden layers: $\{4, 5, 6\}$; hidden layer dimension: $\{32, 64\}$; learning rate $\{5e-3, 1e-3, 5e-4\}$. Optimal

hyperparameter set of BioPathNet {'layers': 6, 'hidden_dim': 32, 'num_negatives': 32, 'lr': 0.005, 'adversarial_temperature': 0.5, 'batch_size': 8, 'epochs': 8}. Hyperparameters were optimized on the validation MRR.

1.3 Drug repurposing task

For this task, the hyperparameter search for the BioPathNet model considered the parameters dependent $= \{yes, no\}$, number of hidden layers $= \{2, 4, 6, 8\}$, aggregator function $= \{sum, pna\}$, adversarial temperature $= \{0.5, 1, 2, 5\}$, and number of negative samples $= \{32, 64, 128\}$. The best MRR performance in the KG completion validation set determined the final parameter set. The experiments were repeated for five different data split seeds in the TxGNN code. The mean ± standard deviation of performance metrics were reported.

1.4 Synthetic lethality gene pair prediction task

We train BioPathNet for 15 epochs and for five random seeds and tuned hyperparameters such as the number of hidden layers in $\{4, 5\}$, the number of sampled negatives per positive example in $\{32, 64\}$, and the adversarial temperature in $\{0.5, 1.0, 2.0, 5.0\}$. Optimal hyperparameter set of BioPathNet {'layers': 5, 'hidden_dim': 32, 'num_negatives': 64, 'lr': 0.005, 'adversarial_temperature': 0.5, 'batch_size': 64, 'epochs': 15}.

1.5 LncRNA-target prediction task

The KG constructed by LncTarD 2.0 consists mainly of a densely connected subgraph of 2,646 genes and 6,084 involved regulations, surrounded by many isolated subgraphs containing only two or three genes. Only the largest connected component was kept and split into train, validation, and test set using the python package PyKeen [5], at a ratio of 0.8, 0.1, and 0.1, while ensuring that all nodes were included in the training set with at least one triplet. The optimal hyperparameter set of BioPathNet in this setting was: {'layers': 6, 'hidden_dim': 32, 'num_negatives': 32, 'lr': 0.005, 'adversarial_temperature': 0.5, 'batch_size': 64, 'epochs': 10}.

1.6 Baseline methods used for comparison

Relational Graph Convolutional Networks (R-GCNs) R-GCN is a variant of the Graph Convolutional Network (GCN) specifically designed to handle graphs with multiple types of relations. R-GCN extends GCN to incorporate relational data, making it suitable for KGs. R-GCN employs a neural encoder to learn the representation for each node, while the decoder retains the scoring functions from the KGE models. Initially, the embeddings for each node are initialized [6]. During each layer of the GNN's message passing, messages are passed, aggregated, and used to update the node's embedding. The messages are obtained by applying a $W_{r,M}^{(l)}$ matrix on the embeddings of the previous layer $h_i^{(l-1)}$ separately for each relation r, allowing R-GCN to handle graphs with multiple types of edges by learning separate weight matrices for each relation type: $m_{r,i}^{(l)} = W_{r,M}^{(l)} h_i^{(l-1)}$. Next, the incoming messages of each node v_i from the neighboring nodes $N_r(i)$ are aggregated by the taking the average: $\widetilde{m_{r,i}^{(l)}} = \frac{1}{|N_r(l)|} \sum_{j \in N_r(i)} m_{r,j}^{(l)}$. Finally, the node's embedding from the previous layer is updated by $h_i^{(l)} = h_i^{(l-1)} + \sum_{r \in T_R} \widetilde{m_{r,i}^{(l)}}$. This process ensures that each node's embedding is updated with information from its neighbors, considering the type of relation connecting them. The final embeddings of the nodes are derived after L layers of propagation. Hyperparameters were optimized via grid search to maximize the MRR performance on the validation set. Both layer normalization and shortcut connections were enabled to stabilize training. Final sets of parameters were:

Gene function: {'learningrate': 10^{-5}, 'input_dim': 128, 'hidden_dims': 128, 'embedding_dims': 128, 'hidden_layers': 5, 'binary cross entropy loss with positive weight': 10, 'negatives_per_positive': 32 }

Drug repurposing (all disease split): {'learningrate': 10^{-5}, 'input_dim': 512, 'hidden_dims': 512, 'embedding_dims': 128, 'hidden_layers': 5, 'binary cross entropy loss with positive weight': 10, 'negatives_per_positive': 10 }

Synthetic lethality: {'learningrate': 10^{-5}, 'input_dim': 128, 'hidden_dims': 128, 'embedding_dims': 128, 'hidden_layers': 5, 'binary cross entropy loss with positive weight': 10, 'negatives_per_positive': 64 }

LncRNA: {'learningrate': 10^{-5}, 'input_dim': 128, 'hidden_dims': 128, 'embedding_dims': 128, 'hidden_layers': 5, 'binary cross entropy loss with positive weight': 10, 'negatives_per_positive': 64 }

The code for training is an efficient re-implementation of the original algorithm and can be found at the following link: https://github.com/DeepGraphLearning/torchdrug (inside /torchdrug/models/gcn.py).

Heterogeneous Graph Transformer (HGT) The Heterogeneous Graph Transformer (HGT) is a GNN-based framework that models heterogeneous graphs by incorporating node- and edge-type-dependent parameters. This enables HGT to capture heterogeneous attention while maintaining distinct representations for different node and edge types. Rather than assigning parameters to each edge type, HGT models mutual attention by decomposing each edge e(u,v)e(u,v) into meta-relation triples ⟨⟨node type u, edge type r, node type v⟩⟩. These meta-relations define weight matrices for attention computation, preserving distinct representation spaces while enabling cross-type message passing. Additionally, HGT introduces a relative temporal encoding technique to capture dynamic structural dependencies in heterogeneous graphs and employs a heterogeneous mini-batch sampling strategy for efficient and scalable training. Hyperparameters were optimized via grid search to maximize the MRR performance on the validation set. Specifically the input embedding dimension was tuned between 256 and 512 and various hidden dimension configurations were explored. Both layer and batch normalization were enabled to stabilize training while residual connections were evaluated for each task. Final hyperparameters were:

Gene function: {'learning_rate': 0.0008, 'weight_decay': 0.000001, 'dropout_rate': 0, 'input_dim': 256, 'hidden_dim': [128, 128], 'loss_margin': 20, 'epochs': 200, 'negatives_per_positive': 10}

Drug repurposing (all disease splits): {'learning_rate': 0.002, 'weight_decay': 0.0005, 'dropout_rate': 0.23, 'input_dim': 256, 'hidden_dim': [64, 64], 'loss_margin': 10, 'epochs': 50, 'negatives_per_positive': 10}

Synthetic lethality: {'learning_rate': 0.0005, 'weight_decay': 0.00001, 'dropout_rate': 0.1, 'input_dim': 128, 'hidden_dim': [128, 256], 'loss_margin': 20, 'epochs': 300, 'negatives_per_positive': 64}

LncRNA: {'learning_rate': 0.00005, 'weight_decay': 0.00001, 'dropout_rate': 0.1, 'input_dim': 64, 'hidden_dim': [64, 128], 'loss_margin': 20, 'epochs': 300, 'negatives_per_positive': 10}

Relational Aware Graph Attention Network (RAGAT) RAGAT is a GAT-based framework that constructs distinct message functions for different relations, leveraging relation-specific parameters to capture the heterogeneous nature of knowledge graphs. Specifically, RAGAT first combines entity and relation embeddings grouped by identical relations to generate relation-entity embeddings. These embeddings are then transformed into messages using shared weight matrices across the graph. To aggregate these messages, the model employs multi-head attention with distinct learned parameters, enabling it to attend to information from multiple representation subspaces simultaneously. Finally, the entity and relation embeddings are fed into a decoder to compute triplet scores. Due to computational constraints and runtime limitations, a comprehensive hyperparameter search for RAGAT across all three applications was not feasible. Instead, we experimented with two default parameter sets provided by the authors for the WN18RR and FB15k-237 datasets, respectively. As a first step, we trained RAGAT using these two sets of hyperparameters on a single seed. We then selected the best-performing model based on MRR validation performance. The hyperparameter combination yielding the highest MRR

was used for all subsequent training. While the default number of training epochs was set to 1500, we further optimized this in a second step by analyzing loss and MRR curves to determine the appropriate number of epochs for each task. Finally, using the best-performing hyperparameter combination from the first step and the optimal number of epochs from the second step, we trained the remaining four seeds on the full graph (G1+BRG). The code for training RAGAT can be found at the following link: https://github.com/liuxiyang641/RAGAT.

BIONIC BIONIC (Biological Network Integration using Convolutions) is a method for integrating biological networks using GCNs to learn unified gene embeddings from multiple input networks. It can operate in unsupervised or semi-supervised modes, incorporating functional annotations when available. BIONIC processes three input networks—a protein-protein interaction network, a gene co-expression network, and a genetic interaction network—through GCN layers, generating network-specific gene features. Residual connections from each layer to the final GCN layer enable learning across different neighborhood sizes. The features are then aggregated using stochastic gene dropout, producing unified gene embeddings for downstream tasks like gene function prediction. Training can be unsupervised, optimizing network reconstruction fidelity, or supervised when labels are available. Since the original BIONIC paper and repository provide input networks only for yeast, we used human networks from the BERTWalk repository (https://github.com/raminass/BERTwalk/tree/main/inputs), which employs an unsupervised learning scheme combining BERT-style masked language modeling with network propagation regularization. BERTWalk performed comparably to BIONIC in gene function prediction. Using BIONIC's default architecture and hyperparameters, we tested two training scenarios: (1) a semi-supervised approach using gene-KEGG functional triples from BioPathNet's training and validation set, as described in the BIONIC paper, and (2) an unsupervised approach where we extracted unified gene embeddings and trained an MLP to minimize cross-entropy loss between positives and sampled negatives. The latter achieved superior performance and was used for benchmarking throughout this study. The code for training BIONIC can be found at the following link: https://github.com/bowang-lab/BIONIC.

Transductive Graph Neural Network (TxGNN) TxGNN is a graph neural network-based model designed to predict drug-disease relationships in zero-shot scenarios where there is minimal prior information or treatment history. Utilizing PrimeKG, a comprehensive biomedical knowledge graph, TxGNN employs R-GCNs to learn embeddings of drugs and diseases, capturing complex interactions by mapping them into a shared latent space. This allows TxGNN to predict drug-disease interactions, focusing on indications and contraindications, even for diseases with limited molecular characterization. It achieves this through the use of disease signature vectors, adaptive embedding aggregation, and iterative optimization of non-linear transformations. TxGNN improves drug-disease prediction by splitting training into pre-training and fine-tuning phases. In pre-training, all triplets are used, while fine-tuning focuses on drug-disease relations like `indication`, `contraindications`, and `off-label use`. This is in contrast to BioPathNet, where we do not divide our approach into these two phases or perform pre-training. Instead, we use all triplets containing non-drug-disease relations in the message passing BRG without considering them for supervision. TxGNN was trained using the hyperparameters provided in the original publication: pretraining: {'lr': $1e-3$, 'batch_size': 1024, 'pretrain epochs': 2}; finetuning: {'lr': $5e-4$, 'hidden_dim': 512, 'batch_size': 1024, 'pretrain epochs': 500}. The hyperparameters used in fine-tuning were the same for all disease splits. The code used for training can be found here: https://github.com/mims-harvard/TxGNN.

DREAMWalk DREAMWalk is a deep learning algorithm for drug repurposing that explores associations through multi-layer random walks. Its fixed hierarchical biomedKG integrates drug-drug and disease-disease similarity networks with links to a PPI network. By leveraging semantic information and Guilt-by-Association, it identifies meaningful paths. Random walks generate embeddings via a heterogeneous Skip-gram model, which are fed into an XGBoost classifier to predict drug-disease associations. Since the original DreamWalk graph (built from ATC and MeSH hierarchies) was unavailable, we reconstructed its key components for comparability with TxGNN and BioPathNet. In one variant (DreamWalk_IC), we computed drug-drug and disease-disease semantic similarities

using PrimeKG's ATC and MONDO hierarchies, integrating PPIs and gene-protein interactions. In another (DreamWalk_OC), we extracted drug and disease connections directly from PrimeKG. Using DreamWalk's default settings, we generated random walks, created embedding vectors, and trained an XGBoost classifier on the same train/validation triples as BioPathNet and TxGNN, evaluating performance on the shared test set.

Knowledge Representation for Synthetic Lethality (KR4SL) KR4SL is a path-representation learning GNN-based method for the prediction of SL gene pairs. The framework begins with the construction of a heterogeneous KG that integrates genes, gene interactions, pathways, GO terms, and an SL graph augmented with reverse and identity edges. This graph is processed through an encoder that identifies relevant relational paths for each primary gene, combining structural graph information with textual semantics of entities. KR4SL utilizes a gated recurrent unit (GRU) to enhance the sequential semantics of the relational paths. As messages propagate through the network, they are enriched by fusing structural and textual information. An attentive aggregation mechanism evaluates the importance of different edges, focusing on the most essential paths for model explanations. Finally, a scoring decoder processes the semantic representations of candidate SL partners, generating scores to rank and select the top-k candidates as the most probable SL partners. The code used for trinaing can be found here: https://github.com/JieZheng-ShanghaiTech/KR4SL.

We use the code from GitHub https://github.com/JieZheng-ShanghaiTech/KR4SL to train KR4SL in the transductive and inductive settings. In transductive setting, we trained KR4SL on the original (unthresholded) data using the same hyperparameters specified by the authors on GitHub, 'weight decay rate': 0.000089, 'lr': 0.0011, 'batch_size': 50, 'epochs': 15, 'hidden_dim': 48, 'drop_out': 0}. The experiments were run five times with different seeds. The same set of parameters was used for thresholded data.To train KR4SL in inductive setting, we used the pre-processed KR4SL dataset, where synthetic lethality (SL) genes were split 60:40 into training and test sets. Subgraphs were then created from the original knowledge graph (KG) to ensure the test genes were unseen during training. Additionally, SL gene triplets from the training set were further split into training and validation sets, ensuring no overlap of SL genes or nodes from the BRG between these subsets. Training and validation losses were used for optimization, with validation loss guiding hyperparameter selection based on MRR, while final performance was assessed on the test set.

DeepLGP: a deep learning method for prioritizing lncRNA target genes DeepLGP was used to benchmark BioPathNet on the lncRNA-mRNA prediction task. It is a deep learning method for prioritizing lncRNA target genes by integrating genomic location, expression across 13 tissues, and miRNA-mediated lncRNA-gene pairs. These features are used to construct a gene interaction network, from which a GCN encoder extracts complex representations. The GCN-derived features, along with the original lncRNA and gene features, are then fed into a CNN classifier to distinguish true lncRNA-gene pairs from false ones. DeepLGP was trained on data from LncRNA2Target v2.0. To ensure a fair comparison, we converted the original code (https://github.com/zty2009/LncRNA-target-gene) to PyTorch and made modifications, including training on 5 seeds for 30 epochs and selecting the best checkpoint based on validation performance. Since BioPathNet and DeepLGP rely on different datasets and regenerating genomic location and expression features no a different set of lncRNA and genes would be too time-consuming, each model was trained on its own dataset but evaluated on their shared test set intersection. This included 6 common lncRNAs (head nodes), 19 common target genes (tail nodes), and no shared connections. While not ideal, training both methods on the small intersection of their training sets (common lncRNA-mRNA pairs) was unfeasible due to insufficient data for meaningful learning.

1.7 Model Evaluation

Models were evaluated by ranking positive triplets $\langle u, r, v \rangle$ of the test set against all negative triplets $\langle u, r, v' \rangle$ that are not present in the KG, following the filtered ranking protocol [7]. Equally, for the reverse relation direction r^{-1}, positive triplets of the form $\langle v, r, u \rangle$ are ranked against all negatives $\langle v, r^{-1}, u \rangle$. This approach yields a very stringent evaluation that forces the model to rank positive samples highly.

The metrics used are mean rank (MR), mean reciprocal rank (MRR), and hits at k (Hits@k). MR is calculated by averaging the ranks q of the positive samples among negatives. Lower values are ideal, with the best value at 1.

$$MR = \frac{1}{|Q|} \sum_{q \in Q} q \tag{10}$$

MRR is the average of the reciprocal rank $\frac{1}{q}$, placing less emphasis on lowly ranked triplets. The values range in $[0, 1]$, and the larger the value, the better the model.

$$MRR = \frac{1}{|Q|} \sum_{q \in Q} \frac{1}{q} \tag{11}$$

Finally, the metric Hits@k provides the probability of correct predictions in the top k predicted triplets made by the model. It can also be considered the percentage of positive triplets in the top k and is, therefore, equivalent to Recall@k. Like MRR, values range in $[0, 1]$, and the larger the value, the better the model.

$$H@k = \frac{\big|\{q \in Q : q < k\}\big|}{\big|Q\big|} \tag{12}$$

The best model is selected based on the highest MRR in a validation set.

While the conditional probability is modeled in KGC by predicting the tail entity v given the head entity u and the relation r, it might be sensible to evaluate under the joint probability of u, v, and r. Therefore, to remain consistent with the TxGNN we compared against in the drug prediction task, we also used AUPRC, which summarizes the precision and recall at different probability thresholds, as well as specificity and F1 score at the probability threshold of 0.5 (equation 13) using code inherent to TxGNN. They evaluate the performance per disease node.

$$Precision = \frac{TP}{TP + FP},\ Recall = \frac{TP}{TP + FN},\ F1 = 2 \times \frac{Precision \times Recall}{Precision + Recall} \tag{13}$$

TxGNN calculates AUPRC using two strategies. The first compares each positive ground truth item vs. one negative from the list of drugs of a disease area, referred to as `AUPRC 1:1`. The second considers all positive and negative ground truth items, referred to as `AUPRC`. The first metric conveys information on the model's ability to distinguish a positive from a randomly sampled negative in direct comparison. In contrast, the latter focuses on distinguishing positives and negatives across the whole dataset. Furthermore, the `AUPRC` accounts for the positive/negative class imbalance and provides a balanced evaluation of precision and recall. We chose to predominately use the latter, which reflects a real-world scenario of identifying therapeutical opportunities within a given set of drugs.

For the SL prediction task, we compared the seed-wise performance of our model with the performance of KR4SL using metrics inherent to the KR4SL framework's code: NDCG@k, Recall@k, and Precision@k. Moreover, we computed MRR from equation 11 for both BioPathNet and KR4SL by first calculating MRR for each query gene and then averaging gene-wise MRR overall query genes.

Recall@k here is utilized to evaluate how effectively a model predicts SL relationships within its top k predictions and is defined as follows:

$$Recall@k = \frac{1}{N} \sum_{n=1}^{N} \frac{\sum_{i=1}^{k} \mathbb{1}\{G_n^{KR}(i) \in G_n^{KP}\}}{|G_n^{KP}|} \tag{14}$$

where N is the total number of query genes, G_n^{KP} is the set of known SL partner candidates of gene n and G_n^{KR} is the set of top-k SL gene partners for gene n ranked by prediction scores meaning that $G_n^{KR}(i)$ is i-th predicted SL partner for gene n.

Respectively, Precision@k is employed to assess how effectively a model predicts SL relationships within its top-k predictions, normalized by either the size of the top-k predictions or the size of the known SL relationships, whichever is smaller. Precision@k is defined as follows:

$$Precision@k = \frac{1}{N}\sum_{n=1}^{N}\frac{\sum_{i=1}^{k}\mathbb{1}\{G_n^{KR}(i) \in G_n^{KP}\}}{min\{|G_n^{KP}|, k\}} \tag{15}$$

NDCG@k is used to evaluate a model's capability to prioritize the top-k candidate genes associated with SL relationships and is defined as follows:

$$NDCG@k = \frac{1}{N}\sum_{n=1}^{N}\frac{DCG@k(n)}{IDCG@k(n)} \tag{16}$$

where $DCG@k(n)$ and $IDCG@k(n)$ are defined as follows:

$$DCG@k(n) = \sum_{i=1}^{k}\frac{2^{\mathbb{1}\{G_n^{KR}(i)\in G_n^{KP}\}}-1}{log_2(i+1)} \tag{17}$$

$$IDCG@k(n) = \sum_{i=1}^{min\{|G_n^{KP}|,k\}}\frac{1}{log_2(i+1)} \tag{18}$$

2 Supplementary Tables

2.1 Supplementary Table 1

Supplementary Table 1: Summary of the dataset for the gene function prediction task: the **number of nodes, edges and relation types** in the background regulatory graph (BRG) obtained from Pathway Commons, as well as train, validation and test set obtained from KEGG.

	Number of nodes	Number of edges	Number of relations
BRG	30,885	1,884,146	13
Train	7,070	22,702	1
Valid	2,348	3,243	1
Test	3,533	6,487	1
Unique count	31,410	1,916,513	14

2.2 Supplementary Table 2

Supplementary Table 2: **Combined Detailed Breakdown of Relation Types** in the functional annotation data set: **Number of triplets per relation type** in BRG, train, validation and test set.

Entity 1	Relation	Entity 2	Total Count	Dataset Breakdown
chem	chemical-affects	genes	618,094	BRG
genes	interacts-with	genes	517,390	BRG
genes	controls-state-change-of	genes	191,548	BRG
genes	in-complex-with	genes	191,275	BRG
genes	catalysis-precedes	genes	147,948	BRG
genes	controls-expression-of	genes	125,860	BRG
genes	KEGGPathway	KEGG	32,367	Train: 22,702; Valid: 3,227; Test: 6,438
chem	consumption-controlled-by	genes	22,659	BRG
genes	controls-production-of	chem	21,262	BRG
genes	controls-phosphorylation-of	genes	17,660	BRG
chem	used-to-produce	chem	14,747	BRG
genes	controls-transport-of	genes	7,899	BRG
chem	reacts-with	chem	4,063	BRG
genes	controls-transport-of-chemical	chem	3,741	BRG

2.3 Supplementary Table 3

Supplementary Table 3: **Number of diseases per disease area split**, as well as the number of edges used as BRG and for training, validation, and testing.

Disease area	Number of diseases	BRG	Train	Valid	Test contraindication	Test indication
Adrenal gland	6	5,728,452	33,063	4,723	303	33
Anemia	19	5,705,775	33,715	4,817	752	88
Cardiovascular	111	5,695,332	30,930	4,419	4,215	453
Cell proliferation	201	5,689,920	33,102	4,729	1,047	999
Mental health	60	5,690,512	33,443	4,778	1,567	355

2.4 Supplementary Table 4

Supplementary Table 4: **Performance comparison between BioPathNet and state-of-the-art** SL gene pair prediction method KR4SL for **unthresholded data** in **transductive setting**.

	seed 1234		seed 1235		seed 1236		seed 1237		seed 1238	
metric	KR4SL	BioPathNet	KR4SL	BioPathNet	KR4SL	BioPathNet	KR4SL	BioPathNet	KR4SL	BioPathNet
MRR	0.285	**0.313**	0.284	**0.309**	0.285	**0.309**	0.280	**0.314**	0.284	**0.312**
NDCG@10	0.336	**0.364**	0.343	**0.362**	0.341	**0.360**	0.337	**0.366**	0.340	**0.366**
NDCG@20	0.351	**0.383**	0.357	**0.381**	0.355	**0.378**	0.352	**0.383**	0.354	**0.384**
NDCG@50	0.364	**0.400**	0.369	**0.397**	0.367	**0.394**	0.365	**0.400**	0.367	**0.401**
Precision@10	0.436	**0.451**	0.436	**0.455**	0.435	**0.450**	0.433	**0.452**	0.437	**0.455**
Precision@20	0.484	**0.515**	0.483	**0.519**	0.483	**0.511**	0.480	**0.511**	0.483	**0.516**
Precision@50	0.529	**0.580**	0.531	**0.581**	0.527	**0.572**	0.528	**0.574**	0.529	**0.580**
Recall@10	0.426	**0.440**	0.425	**0.444**	0.424	**0.440**	0.422	**0.441**	0.427	**0.444**
Recall@20	0.480	**0.511**	0.479	**0.515**	0.479	**0.507**	0.476	**0.507**	0.479	**0.512**
Recall@50	0.529	**0.579**	0.530	**0.580**	0.526	**0.571**	0.527	**0.573**	0.529	**0.579**

2.5 Supplementary Table 5

Supplementary Table 5: **Performance comparison between BioPathNet and state-of-the-art** SL gene pair prediction method KR4SL for **thresholded data** in **transductive setting**.

	seed 1234		seed 1235		seed 1236		seed 1237		seed 1238	
metric	KR4SL	BioPathNet	KR4SL	BioPathNet	KR4SL	BioPathNet	KR4SL	BioPathNet	KR4SL	BioPathNet
MRR	0.338	**0.362**	0.339	**0.361**	0.336	**0.350**	0.333	**0.364**	0.339	**0.356**
NDCG@10	0.401	**0.420**	0.401	**0.416**	0.400	**0.406**	0.399	**0.418**	0.399	**0.412**
NDCG@20	0.417	**0.437**	0.417	**0.434**	0.416	**0.427**	0.414	**0.436**	0.416	**0.432**
NDCG@50	0.430	**0.453**	0.430	**0.451**	0.429	**0.443**	0.427	**0.452**	0.428	**0.448**
Precision@10	0.514	**0.525**	0.513	**0.519**	**0.515**	0.508	0.510	**0.519**	0.513	**0.517**
Precision@20	0.568	**0.583**	0.564	**0.581**	0.567	**0.579**	0.564	**0.581**	0.569	**0.584**
Precision@50	0.614	**0.641**	0.611	**0.644**	0.613	**0.641**	0.610	**0.638**	0.614	**0.642**
Recall@10	0.502	**0.514**	0.501	**0.508**	**0.503**	0.498	0.498	**0.507**	0.501	**0.506**
Recall@20	0.564	**0.579**	0.560	**0.577**	0.562	**0.575**	0.560	**0.576**	0.564	**0.580**
Recall@50	0.613	**0.640**	0.610	**0.643**	0.612	**0.640**	0.610	**0.637**	0.613	**0.641**

2.6 Supplementary Table 6

Supplementary Table 6: **Effect of negative sampling on BioPathNet performances - Drug-disease indication and contra-indication predictions.**

**Adrenal Gland Indication**	MRR	Hits@1	Hits@3	Hits@10
Baseline AN	0.73 ± 0.06	0.600 ± 0.08	0.87 ± 0.06	1.000 ± 0.000
NTA	0.83 ± 0.04	0.67 ± 0.06	1.000 ± 0.000	1.000 ± 0.000
NTA+SANS	0.81 ± 0.03	0.61 ± 0.06	1.0 ± 0.0	1.0 ± 0.0
Δ(NTA-AN)	0.1 ± 0.07	0.07 ± 0.1	0.13 ± 0.06	0
increment(NTA-AN)%	13.70%	11.70%	14.90%	0%
Δ(NTA+SANS-AN)	0.08 ± 0.06	0.01 ± 0.1	0.13 ± 0.06	0
increment(NTA+SANS-AN)%	11%	1.60%	14.90%	0%
**Adrenal Gland contra-indication**	MRR	Hits@1	Hits@3	Hits@10
Baseline AN	0.96 ± 0.04	0.94 ± 0.06	1.000 ± 0.000	1.000 ± 0.000
NTA	0.94 ± 0.03	0.88 ± 0.06	1.000 ± 0.000	1.000 ± 0.000
NTA+SANS	0.96 ± 0.04	0.92 ± 0.08	1.0 ± 0.0	1.0 ± 0.0
Δ(NTA-AN)	- 0.02 ± 0.05	- 0.06 ± 0.08	0	0
increment(NTA-AN)%	-2%	-6.30%	0%	0%
Δ(NTA+SANS-AN)	0	-0.02 ± 0.1	0	0
increment(NTA+SANS-AN)%	0%	-2.10%	0%	0%

**Anemia indicatiion**	MRR	Hits@1	Hits@3	Hits@10
Baseline AN	0.43 ± 0.02	0.35 ± 0.05	0.458 ± 0.02	0.562 ± 0.04
NTA	0.458 ± 0.004	0.417 ± 0.000	0.44 ± 0.01	0.58 ± 0.04
NTA+SANS	0.47 ± 0.03	0.44 ± 0.03	0.47 ± 0.03	0.56 ± 0.07
Δ(NTA-AN)	0.03 ± 0.02	0.07 ± 0.05	- 0.02 ± 0.02	0.02 ± 0.07
increment(NTA-AN)%	7%	20%	-4.20%	3.60%
Δ(NTA+SANS-AN)	0.04 ± 0.03	0.09 ± 0.06	0.01 ± 0.03	0
increment(NTA+SANS-AN)%	9.30%	25.70%	2.20%	0%
**Anemia contra-indication**	MRR	Hits@1	Hits@3	Hits@10
Baseline AN	0.59 ± 0.05	0.48 ± 0.07	0.63 ± 0.03	0.78 ± 0.03
NTA	0.59 ± 0.02	0.500 ± 0.03	0.62 ± 0.01	0.81 ± 0.04
NTA+SANS	0.58 ± 0.02	0.45 ± 0.05	0.67 ± 0.01	0.82 ± 0.03
Δ(NTA-AN)	0 ± 0.05	0.02 ± 0.07	-0.01 ± 0.03	0.03 ± 0.05
increment(NTA-AN)%	0%	4.20%	-1.50%	3.80%
Δ(NTA+SANS-AN)	-0.01 ± 0.06	-0.03 ± 0.08	0.04 ± 0.03	0.04 ± 0.04
increment(NTA+SANS-AN)%	-1.70%	-6.20%	6%	5.10%

**Cardiovascular indication**	MRR	Hits@1	Hits@3	Hits@10
Baseline AN	0.223 ± 0.003	0.170 ± 0.000	0.233 ± 0.005	0.33 ± 0.02
NTA	0.23 ± 0.008	0.19 ± 0.01	0.24 ± 0.02	0.301 ± 0.02
NTA+SANS	0.20 ± 0.01	0.14 ± 0.02	0.19 ± 0.01	0.34 ± 0.02
Δ(NTA-AN)	0.01 ± 0.008	0.02 ± 0.01	0.007 ± 0.02	-0.02 ± 0.03
increment(NTA-AN)%	4.50%	11.70%	3.10%	-6%
Δ(NTA+SANS-AN)	-0.02 ± 0.01	-0.03 ± 0.02	-0.04 ± 0.01	0.01 ± 0.03
increment(NTA+SANS-AN)%	-9%	-17.00%	-17%	3%
**Cardiovascular contra-indication**	MRR	Hits@1	Hits@3	Hits@10
Baseline AN	0.284 ± 0.003	0.187 ± 0.007	0.323 ± 0.005	0.480 ± 0.004
NTA	0.26 ± 0.02	0.16 ± 0.02	0.29 ± 0.03	0.43 ± 0.02
NTA+SANS	0.25 ± 0.03	0.14 ± 0.03	0.29 ± 0.04	0.44 ± 0.02
Δ(NTA-AN)	-0.02 ± 0.02	-0.03 ± 0.02	-0.03 ± 0.03	-0.05 ± 0.02
increment(NTA-AN)%	-7%	-16%	-9.30%	-10%
Δ(NTA+SANS-AN)	-0.03 ± 0.02	-0.05 ± 0.03	-0.03 ± 0.03	-0.04 ± 0.02
increment(NTA+SANS-AN)%	-10%	-26%	-9.30%	-8.30%

**Cell proliferation indication**	MRR	Hits@1	Hits@3	Hits@10
Baseline AN	0.603 ± 0.006	0.55 ± 0.01	0.63 ± 0.008	0.711 ± 0.008
NTA	0.61 ± 0.009	0.55 ± 0.03	0.65 ± 0.009	0.725 ± 0.007
NTA+SANS	0.62 ± 0.007	0.55 ± 0.02	0.67 ± 0.02	0.75 ± 0.02
Δ(NTA-AN)	0.013 ± 0.01	0	0.02 ± 0.01	0.01 ± 0.09
increment(NTA-AN)%	1.60%	0%	3%	1.40%
Δ(NTA+SANS-AN)	0.02 ± 0.009	0	0.04 ± 0.02	0.04 ± 0.02
increment(NTA+SANS-AN)%	3.40%	0%	6.30%	5.70%
**Cell proliferation contra-indication**	MRR	Hits@1	Hits@3	Hits@10
Baseline AN	0.68 ± 0.006	0.63 ± 0.007	0.710 ± 0.005	0.78 ± 0.01
NTA	0.68 ± 0.007	0.63 ± 0.007	0.710 ± 0.005	0.75 ± 0.02
NTA+SANS	0.69 ± 0.005	0.65 ± 0.006	0.699 ± 0.006	0.76 ± 0.009
Δ(NTA-AN)	0	0	0	-0.03 ± 0.03
increment(NTA-AN)%	0%	0%	0%	-3.80%
Δ(NTA+SANS-AN)	0.01 ± 0.008	0.02 ± 0.009	0	-0.02 ± 0.03
increment(NTA+SANS-AN)%	1.50%	3.20%	0%	-2.60%

**Mental Health indication**	MRR	Hits@1	Hits@3	Hits@10
Baseline AN	0.35 ± 0.006	0.29 ± 0.009	0.36 ± 0.008	0.47 ± 0.006
NTA	0.36 ± 0.006	0.31 ± 0.005	0.35 ± 0.009	0.49 ± 0.006
NTA+SANS	0.35 ± 0.005	0.30 ± 0.006	0.37 ± 0.005	0.46 ± 0.01
Δ(NTA-AN)	0.01 ± 0.008	0.02 ± 0.01	-0.01 ± 0.01	0.02 ± 0.008
increment(NTA-AN)%	2.80%	6.90%	-2.8%	4.30%
Δ(NTA+SANS-AN)	0	0.01 ± 0.03	0.01 ± 0.01	-0.01 ± 0.01
increment(NTA+SANS-AN)%	0%	3.40%	2.80%	-2.10%
**Mental Health contra-indication**	MRR	Hits@1	Hits@3	Hits@10
Baseline AN	0.21 ± 0.006	0.12 ± 0.008	0.23 ± 0.009	0.29 ± 0.02
NTA	0.22 ± 0.007	0.16 ± 0.01	0.23 ± 0.0208	0.288 ± 0.009
NTA+SANS	0.160 ± 0.008	0.11 ± 0.01	0.17 ± 0.01	0.28 ± 0.01
Δ(NTA-AN)	0.01 ± 0.009	0.04 ± 0.01	0	-0.002 ± 0.02
increment(NTA-AN)%	4.70%	33%	0%	-0.7 %
Δ(NTA+SANS-AN)	-0.05 ± 0.01	-0.01 ± 0.01	-0.06 ± 0.03	-0.01 ± 0.01
increment(NTA+SANS-AN)%	-10.60%	-8.30%	-26%	-3.4 %

2.7 Supplementary Table 7

Supplementary Table 7: **Effect of negative sampling on BioPathNet performances - Gene Function prediction.** We report metric averages across five seeds along with their standard errors (SE). Delta values represent the average differences between two settings, with the corresponding SE of the mean difference also provided. Baseline Adversarial Negative (AN) corresponds to BioPathNet with adversarial negative sampling; NTA corresponds to Negative Type Aware negative sampling and SANS corresponds to Structure Aware Negative Sampling. Percentage changes between the original BioPathNet and its ablated version are also reported. Positive changes (green) indicate performance improvements, while negative changes (red) denote declines.

Gene function prediction	MRR	Hits@1	Hits@3	Hits@10
Baseline AN	0.526 ± 0.003	0.430 ± 0.003	0.574 ± 0.004	0.711 ± 0.002
NTA	0.547 ± 0.002	0.457 ± 0.004	0.593 ± 0.006	0.723 ± 0.009
NTA+SANS	0.552 ± 0.003	0.461 ± 0.007	0.598 ± 0.005	0.728 ± 0.006
Δ(NTA-AN)	0.021 ± 0.003	0.027 ± 0.003	0.019 ± 0.007	0.012 ± 0.009
increment(NTA-AN)%	4%	6.20%	3.30%	2%
Δ(NTA+SANS-AN)	0.026 ± 0.004	0.031 ± 0.008	0.024 ± 0.008	0.017 ± 0.006
increment(NTA+SANS-AN)%	5.00%	7.30%	4.20%	2.40%

2.8 Supplementary Table 8

Supplementary Table 8: **Effect of negative sampling on BioPathNet performances - Synthetic lethality gene pair prediction.**

**Syn leth (untresholded)**	MRR	Hits@1	Hits@3	Hits@10
Baseline AN	0.3035 ± 0.0007	0.1778 ± 0.0008	0.279 ± 0.001	0.4373 ± 0.0001
NTA	0.311 ± 0.002	0.1815 ± 0.0005	0.2933 ± 0.0009	0.4434 ± 0.0003
NTA+SANS	0.3145 ± 0.0005	0.1857 ± 0.0005	0.2926 ± 0.0005	0.4429 ± 0.0002
Δ(NTA-AN)	0.008 ± 0.003	0.0037 ± 0.009	0.014 ± 0.002	0.0061 ± 0.0003
increment(NTA-AN)%	2.60%	2%	5%	1.40%
Δ(NTA+SANS-AN)	0.011 ± 0.002	0.008 ± 0.0009	0.014 ± 0.001	0.006 ± 0.0003
increment(NTA+SANS-AN)%	4%	4.50%	5%	1.40%
**Syn leth (tresholded)**	MRR	Hits@1	Hits@3	Hits@10
Baseline AN	0.3493 ± 0.0003	0.2088 ± 0.0009	0.3258 ± 0.0002	0.505 ± 0.001
NTA	0.3586 ± 0.0002	0.2151 ± 0.0004	0.3388 ± 0.0002	0.5094 ± 0.0002
NTA+SANS	0.3614 ± 0.0001	0.2165 ± 0.0004	0.3385 ± 0.0003	0.5099 ± 0.0002
Δ(NTA-AN)	0.009 ± 0.0004	0.0063 ± 0.0009	0.0130 ± 0.0003	0.0044 ± 0.0002
increment(NTA-AN)%	2.60%	3%	4%	0.90%
Δ(NTA+SANS-AN)	0.012 ± 0.0003	0.008 ± 0.001	0.013 ± 0.0003	0.0049 ± 0.0002
increment(NTA+SANS-AN)%	3.30%	3.80%	4.00%	0.97%

2.9 Supplementary Table 9

Supplementary Table 9: **Effect of negative sampling on BioPathNet performances - LncRNA-gene pair prediction.**

lncRNA-gene pairs	MRR	Hits@1	Hits@3	Hits@10
Baseline AN	0.183 ± 0.002	0.089 ± 0.002	0.198 ± 0.003	0.384 ± 0.004
NTA	0.189 ± 0.002	0.095 ± 0.003	0.204 ± 0.003	0.383 ± 0.003
NTA+SANS	0.190 ± 0.002	0.096 ± 0.003	0.203 ± 0.002	0.393 ± 0.001
Δ(NTA-AN)	0.006 ± 0.003	0.006 ± 0.003	0.006 ± 0.004	-0.001 ± 0.004
increment(NTA-AN)%	3.30%	6.60%	3%	-0.2 %
Δ(NTA+SANS-AN)	0.007 ± 0.003	0.007 ± 0.003	0.005 ± 0.003	0.009 ± 0.002
increment(NTA+SANS-AN)%	3.80%	7.90%	2.50%	2.30%

2.10 Supplementary Table 10

Supplementary Table 10: **Effect of BRG on BioPathNet performances.** We report metric averages across five seeds along with their standard errors (SE). Delta values represent the average differences between two settings, with the corresponding SE of the mean difference also provided. Positive changes (green) indicate performance improvements, while negative changes (red) denote declines.

**Gene function prediction**	MRR	Hits@1	Hits@3	Hits@10
G1 (w/o BRG)	0.455 ± 0.001	0.368 ± 0.001	0.489 ± 0.002	0.623 ± 0.001
G1+G2 (with BRG)	0.547 ± 0.002	0.457 ± 0.002	0.593 ± 0.002	0.723 ± 0.002
Δ(with BRG - w/o BRG)	0.092 ± 0.002	0.089 ± 0.002	0.104 ± 0.003	0.100 ± 0.002
increment(with BRG - w/o BRG)%	20%	24%	21.30%	16%
**Drug repurposing adrenal gland**	MRR	Hits@1	Hits@3	Hits@10
G1 (w/o BRG)	0.033 ± 0.01	0.000 ± 0.000	0.000 ± 0.000	0.12 ± 0.1
G1+G2 (with BRG)	0.83 ± 0.04	0.67 ± 0.06	1.000 ± 0.000	1.000 ± 0.000
Δ(with BRG - w/o BRG)	0.79 ± 0.04	0.67 ± 0.06	1.0 ± 0	0.87 ± 0.1
increment(with BRG - w/o BRG)%	>100%	>100%	>100%	>100%
**Drug repurposing anemia**	MRR	Hits@1	Hits@3	Hits@10
G1 (w/o BRG)	0.000 ± 0.000	0.000 ± 0.000	0.000 ± 0.000	0.000 ± 0.000
G1+G2 (with BRG)	0.458 ± 0.004	0.417 ± 0.000	0.438 ± 0.01	0.58 ± 0.04
Δ(with BRG - w/o BRG)	0.458 ± 0.004	0.417 ± 0.000	0.438 ± 0.01	0.58 ± 0.04
increment(with BRG - w/o BRG)%	>100%	>100%	>100%	>100%
**Drug repurposing cardiovascular**	MRR	Hits@1	Hits@3	Hits@10
G1 (w/o BRG)	0.129 ± 0.017	0.075 ± 0.044	0.160 ± 0.019	0.241 ± 0.039
G1+G2 (with BRG)	0.23 ± 0.01	0.19 ± 0.01	0.24 ± 0.02	0.301 ± 0.02
Δ(with BRG - w/o BRG)	0.101 ± 0.02	0.115 ± 0.05	0.15 ± 0.004	0.06 ± 0.04
increment(with BRG - w/o BRG)%	78%	>100%	94%	25%
**Drug repurposing cell proliferation**	MRR	Hits@1	Hits@3	Hits@10
G1 (w/o BRG)	0.043 ± 0.007	0.000 ± 0.000	0.063 ± 0.02	0.170 ± 0.04
G1+G2 (with BRG)	0.61 ± 0.02	0.55 ± 0.03	0.65 ± 0.01	0.725 ± 0.007
Δ(with BRG - w/o BRG)	0.567 ± 0.02	0.55 ± 0.03	0.587 ± 0.03	0.55 ± 0.05
increment(with BRG - w/o BRG)%	>100%	>100%	>100%	>100%
**Drug repurposing mental health**	MRR	Hits@1	Hits@3	Hits@10
G1 (w/o BRG)	0.012 ± 0.006	0.000 ± 0.000	0.019 ± 0.02	0.038 ± 0.02
G1+G2 (with BRG)	0.36 ± 0.01	0.31 ± 0.01	0.35 ± 0.02	0.49 ± 0.03
Δ(with BRG - w/o BRG)	0.35 ± 0.01	0.31 ± 0.01	0.33 ± 0.03	0.45 ± 0.04
increment(with BRG - w/o BRG)%	>100%	>100%	>100%	>100%
**Syn leth (unthresholded)**	MRR	Hits@1	Hits@3	Hits@10
G1 (w/o BRG)	0.1879 ± 0.0009	0.0983 ± 0.0007	0.2013 ± 0.002	0.3591 ± 0.003
G1+G2 (with BRG)	0.311 ± 0.002	0.181 ± 0.005	0.2933 ± 0.0009	0.4434 ± 0.0003
Δ(with BRG - w/o BRG)	0.123 ± 0.001	0.083 ± 0.005	0.0920 ± 0.002	0.0843 ± 0.003
increment(with BRG - w/o BRG)%	65.40%	84%	45.70%	23.50%
**Syn leth (thresholded)**	MRR	Hits@1	Hits@3	Hits@10
G1 (w/o BRG)	0.2263 ± 0.001	0.1183 ± 0.001	0.2454 ± 0.0027 0.436 ± 0.0006	
G1+G2 (with BRG)	0.3586 ± 0.0002	0.2151 ± 0.0004	0.3388 ± 0.0002	0.5094 ± 0.0002
Δ(with BRG - w/o BRG)	0.1323 ± 0.001	0.0968 ± 0.001	0.0934 +± 0.002	0.0734 ± 0.007
increment(with BRG - w/o BRG)%	58.40%	81%	30%	17%
**lncRNA-gene pairs**	MRR	Hits@1	Hits@3	Hits@10
G1 (w/o BRG)	0.189 ± 0.001	0.095 ± 0.001	0.204 ± 0.001	0.383 ± 0.001
G1+G2 (with BRG)	0.182 ± 0.001	0.089 ± 0.002	0.201 ± 0.002	0.377 ± 0.003
Δ(with BRG - w/o BRG)	0.007 ± 0.001	0.006 ± 0.002	0.003 ± 0.002	0.006 ± 0.003
increment(with BRG - w/o BRG)%	4%	6.30%	1.50%	1.60%

2.11 Supplementary Table 11

Supplementary Table 11: **Performances across five different disease area splits**, mean metrics summarized as average across contraindication and indication.

Model	Recall@20	AP@20	MRR@20	F1	AUPRC	MRR	split
dreamwalk_OC	0.11	0.167	0.193	0.008	0.09	0.199	anemia
dreamwalk_IC	0.179	0.276	0.334	0.033	0.146	0.339	
TxGNN	0.441	0.461	**0.531**	0.238	0.35	**0.533**	
BioPathNet	**0.504**	**0.493**	0.52	**0.305**	**0.412**	0.523	
dreamwalk_OC	0.207	0.286	0.305	0	0.188	0.308	adrenal_gland
dreamwalk_IC	0.319	0.466	0.534	0.002	0.25	0.539	
TxGNN	**0.601**	0.734	0.752	**0.615**	0.639	0.752	
BioPathNet	0.559	**0.879**	**0.889**	0.403	**0.694**	**0.889**	
dreamwalk_OC	0.025	0.055	0.066	0	0.036	0.073	cardiovascular
dreamwalk_IC	0.067	0.092	0.107	0	0.047	0.114	
TxGNN	0.098	0.169	0.199	0.06	0.107	0.207	
BioPathNet	**0.166**	**0.23**	**0.249**	**0.074**	**0.136**	**0.256**	
dreamwalk_OC	0.131	0.12	0.136	0.009	0.089	0.144	cell_proliferation
dreamwalk_IC	0.215	0.215	0.246	0.017	0.16	0.253	
TxGNN	0.51	0.485	0.533	0.242	0.428	0.537	
BioPathNet	**0.602**	**0.623**	**0.652**	**0.393**	**0.552**	**0.655**	
dreamwalk_OC	0.039	0.08	0.1	0.002	0.043	0.107	mental_health
dreamwalk_IC	0.081	0.125	0.155	0.003	0.072	0.163	
TxGNN	0.177	0.23	0.269	0.113	0.163	0.275	
BioPathNet	**0.219**	**0.265**	**0.297**	**0.132**	**0.187**	**0.302**	

2.12 Supplementary Table 12

Supplementary Table 12: **Best Hyperparameter Sets for Each Disease Area Split**, *dim* refers to embedding dimension, *L* is the number of hidden layers, *lr* is the learning rate, *Adv. T* the adversarial temperature used in self-adversarial negative sampling, *Aggr. Function* specifies the aggregation function, in this case PNA was always the best performing.

**Disease Area**	*dim*	*L*	*lr*	*NegSam*	*Adv. T*	*Aggr. Function*
Adrenal Gland	32	6	5e-3	64	0.5	PNA
Anemia	32	4	1e-3	64	1.0	PNA
Cardiovascular	32	6	1e-3	64	0.5	PNA
Cell Proliferation	32	6	1e-3	64	1.0	PNA
Mental Health	32	4	5e-3	64	1.0	PNA
Alzheimer	32	6	5e-3	64	1.0	PNA

2.13 Supplementary Table 13

Supplementary Table 13: **Performance comparison between BioPathNet and state-of-the-art** SL gene pair prediction method KR4SL for **unthresholded data** in **inductive setting**.

	seed 1234		seed 1235		seed 1236		seed 1237		seed 1238	
metric	KR4SL	BioPathNet	KR4SL	BioPathNet	KR4SL	BioPathNet	KR4SL	BioPathNet	KR4SL	BioPathNet
MRR	0.271	**0.354**	0.285	**0.359**	0.300	**0.354**	0.299	**0.355**	0.255	**0.346**
NDCG@10	0.344	**0.439**	0.359	**0.442**	0.366	**0.437**	0.369	**0.434**	0.332	**0.432**
NDCG@20	0.357	**0.448**	0.369	**0.450**	0.379	**0.447**	0.380	**0.444**	0.345	**0.442**
NDCG@50	0.372	**0.461**	0.382	**0.463**	0.395	**0.461**	0.396	**0.456**	0.358	**0.454**
Precision@10	0.502	**0.557**	0.513	**0.551**	0.499	**0.552**	0.506	**0.549**	0.500	**0.552**
Precision@20	0.540	**0.586**	0.543	**0.579**	0.536	**0.583**	0.540	**0.582**	0.536	**0.585**
Precision@50	0.596	**0.636**	0.591	**0.625**	0.593	**0.632**	0.594	**0.624**	0.584	**0.626**
Recall@10	0.499	**0.545**	0.509	**0.540**	0.497	**0.541**	0.503	**0.537**	0.497	**0.540**
Recall@20	0.539	**0.582**	0.542	**0.575**	0.535	**0.579**	0.540	**0.577**	0.535	**0.580**
Recall@50	0.596	**0.635**	0.591	**0.624**	0.593	**0.631**	0.594	**0.623**	0.584	**0.625**

2.14 Supplementary Table 14

Supplementary Table 14: **Performance comparison between BioPathNet and state-of-the-art** SL gene pair prediction method KR4SL for **thresholded data** in **inductive setting**.

	seed 1234		seed 1235		seed 1236		seed 1237		seed 1238	
metric	KR4SL	BioPathNet	KR4SL	BioPathNet	KR4SL	BioPathNet	KR4SL	BioPathNet	KR4SL	BioPathNet
MRR	0.316	**0.403**	0.314	**0.409**	0.314	**0.405**	0.348	**0.402**	0.261	**0.397**
NDCG@10	0.396	**0.491**	0.395	**0.497**	0.393	**0.493**	0.422	**0.490**	0.350	**0.486**
NDCG@20	0.408	**0.500**	0.408	**0.508**	0.406	**0.502**	0.434	**0.502**	0.365	**0.497**
NDCG@50	0.423	**0.514**	0.420	**0.521**	0.422	**0.514**	0.449	**0.516**	0.379	**0.509**
Precision@10	0.573	**0.620**	0.573	**0.621**	0.565	**0.622**	0.573	**0.618**	0.558	**0.617**
Precision@20	0.608	**0.647**	0.610	**0.656**	0.604	**0.649**	0.607	**0.653**	0.602	**0.650**
Precision@50	0.661	**0.696**	0.656	**0.700**	0.660	**0.695**	0.657	**0.705**	0.650	**0.695**
Recall@10	0.570	**0.608**	0.570	**0.609**	0.562	**0.610**	0.570	**0.608**	0.555	**0.606**
Recall@20	0.608	**0.643**	0.609	**0.652**	0.603	**0.645**	0.606	**0.649**	0.601	**0.646**
Recall@50	0.660	**0.695**	0.656	**0.700**	0.660	**0.694**	0.657	**0.704**	0.650	**0.694**

2.15 Supplementary Table 15

Supplementary Table 15: **Zero-shot prediction scenario for Alzheimer's disease**: diseases included and number of contraindication and indication drugs in custom data split generated following TxGNN code on disease evaluation.

Disease	ID	# contraindication	# indication
Alzheimer disease	28,780	56	8
Alzheimer disease w/o neurofibrillary tangles	83,960	2	7
Lewy body dementia	29,296	2	0
Pick disease	28,473	27	7
Dementia (disease)	37,573	28	3

2.16 Supplementary Table 16

Supplementary Table 16: **Best hyperparameter sets for gene function prediction tasks** with and without BRG, *dim* refers to embedding dimension, *L* is the number of hidden layers, *lr* is the learning rate, *Adv. T* the adversarial temperature used in self-adversarial negative sampling, *Aggr. Function* specifies the aggregation function, relevant only for BioPathNet that can choose between *sum* and *PNA*.

**Model**	*dim*	*L*	*lr*	*NegSam*	*Adv. T*	*Aggr. Function*
TransE w BRG	128	-	1e-3	512	1	-
TransE w/o BRG	512	-	5e-4	512	1	-
DistMult w BRG	128	-	1e-3	512	1	-
DistMult w/o BRG	512	-	1e-3	512	1	-
RotatE w BRG	128	-	2e-4	512	1	-
RotatE w/o BRG	512	-	5e-4	512	1	-
R-GCN w BRG	128	5	5e-4	32	1	-
R-GCN w/o BRG	256	3	1e-4	64	1	-
BioPathNet w BRG	32	6	5e-3	32	1	PNA
BioPathNet w/o BRG	32	6	5e-3	32	1	PNA

2.17 Supplementary Table 17

Supplementary Table 17: **Detailed breakdown of the relation types** in the Drug Repurposing task: Number of triplets per head, tail and relation type.

Entity 1	Relation	Entity 2	# Triplets
drug	drug_drug	drug	2,664,736
gene/protein	anatomy_protein_present	anatomy	1,444,856
gene/protein	protein_protein	gene/protein	640,170
disease	disease_phenotype_positive	effect/phenotype	144,830
gene/protein	bioprocess_protein	biological_process	137,936
biological_process	bioprocess_bioprocess	biological_process	105,698
gene/protein	cellcomp_protein	cellular_component	79,406
gene/protein	disease_protein	disease	76,278
gene/protein	molfunc_protein	molecular_function	66,242
disease	disease_disease	disease	64,262
drug	drug_effect	effect/phenotype	61,322
gene/protein	pathway_protein	pathway	40,442
effect/phenotype	phenotype_phenotype	effect/phenotype	37,444
drug	contraindication	disease	28,983
anatomy	anatomy_anatomy	anatomy	28,064
molecular_function	molfunc_molfunc	molecular_function	27,136
drug	drug_protein	gene/protein	24,188
gene/protein	anatomy_protein_absent	anatomy	18,970
cellular_component	cellcomp_cellcomp	cellular_component	9,676
drug	indication	disease	8,798
pathway	pathway_pathway	pathway	5,066
exposure	exposure_exposure	exposure	4,136
gene/protein	phenotype_protein	effect/phenotype	3,141
drug	off-label use	disease	2,420
exposure	exposure_disease	disease	2,202
exposure	exposure_bioprocess	biological_process	1,548
exposure	exposure_protein	gene/protein	1,134
disease	disease_phenotype_negative	effect/phenotype	986
exposure	exposure_molfunc	molecular_function	42
exposure	exposure_cellcomp	cellular_component	9

2.18 Supplementary Table 18

Supplementary Table 18: **Detailed breakdown of the relation types** in the synthetic lethality dataset: Number of edges per head and tail type in BRG and SL graph (transductive setting).

			Thr = 0.0		Thr = 0.3	
Entity 1	Relation	Entity 2	# Triples	Split	# Triples	Split
gene	involved in	BP	105,097	BRG		BRG
gene	part of	CC	60,679	BRG		BRG
gene	enables	MF	54,511	BRG		BRG
gene	located in	CC	51,382	BRG		BRG
gene	participates GpPW	Pathway	41,726	BRG		BRG
BP	is a	BP	33,158	BRG		BRG
gene	SL GsG	gene	35,318	Full	31,357	Full
gene	SL GsG	gene	14,858	BRG	13,161	BRG
gene	SL GsG	gene	9,878	Train	8,770	Train
gene	SL GsG	gene	3,533	Valid	3,172	Valid
gene	SL GsG	gene	7,049	Test	6,254	Test
gene	is active in	CC	8,749	BRG		BRG
MF	is a	MF	7,493	BRG		BRG
CC	is a	CC	3,224	BRG		BRG
BP	part of	BP	3,129	BRG		BRG
BP	regulates	BP	2,088	BRG		BRG
BP	negatively regulates	BP	1,853	BRG		BRG
BP	positively regulates	BP	1,829	BRG		BRG
CC	part of	CC	1,222	BRG		BRG
gene	colocalizes with	CC	874	BRG		BRG
gene	contributes to	MF	752	BRG		BRG
MF	part of	BP	722	BRG		BRG
gene	involved in	Pathway	526	BRG		BRG
gene	NOT|involved in	BP	376	BRG		BRG
gene	NOT|enables	MF	233	BRG		BRG
BP	regulates	MF	218	BRG		BRG
gene	acts upstream of or within	BP	202	BRG		BRG
BP	positively regulates	MF	200	BRG		BRG
BP	negatively regulates	MF	192	BRG		BRG
MF	has part	MF	167	BRG		BRG
gene	acts upstream of	BP	158	BRG		BRG
CC	has part	CC	154	BRG		BRG
BP	has part	BP	141	BRG		BRG
gene	NOT|part of	CC	134	BRG		BRG
BP	occurs in	CC	119	BRG		BRG
gene	NOT|located in	CC	109	BRG		BRG
BP	has part	MF	94	BRG		BRG
MF	negatively regulates	MF	50	BRG		BRG
MF	positively regulates	MF	40	BRG		BRG
MF	regulates	MF	36	BRG		BRG
MF	occurs in	CC	31	BRG		BRG
BP	is a	Pathway	30	BRG		BRG
BP	part of	Pathway	15	BRG		BRG
gene	acts upstream of positive effect	BP	15	BRG		BRG
gene	NOT|colocalizes with	CC	9	BRG		BRG
MF	part of	MF	8	BRG		BRG
gene	acts upstream of or within positive effect	BP	7	BRG		BRG
BP	happens during	BP	7	BRG		BRG
BP	negatively regulates	Pathway	5	BRG		BRG
BP	positively regulates	Pathway	5	BRG		BRG
BP	regulates	Pathway	4	BRG		BRG
gene	NOT|is active in	CC	3	BRG		BRG
gene	NOT|contributes to	MF	3	BRG		BRG
BP	has part	Pathway	2	BRG		BRG
gene	acts upstream of negative effect	BP	2	BRG		BRG
gene	acts upstream of or within negative effect	BP	2	BRG		BRG
BP	ends during	BP	1	BRG		BRG
gene	NOT|acts upstream of or within	BP	1	BRG		BRG
gene	NOT|acts upstream of or within negative effect	BP	1	BRG		BRG
MF	part of	Pathway	1	BRG		BRG
MF	regulates	BP	1	BRG		BRG
Pathway	has part	BP	1	BRG		BRG

2.19 Supplementary Table 19

Supplementary Table 19: Summary of the dataset for the synthetic lethality prediction task (transductive setting): the **number of nodes, edges and relation types** in the background regulatory graph (BRG) as well as train, validation and test set obtained from SynLethDB-v2.0 for various thresholds.

Thr = 0.0	Number of nodes	Number of edges	Number of relations
BRG	42,547	396,619	32
Train	5,444	9,878	1
Valid	2,918	3,533	1
Test	4,474	7,049	1
Unique count	42,547	417,079	32
Thr = 0.2	Number of nodes	Number of edges	Number of relations
BRG	42,547	396,221	32
Train	5,363	9,617	1
Valid	2,870	3,447	1
Test	4,407	6,887	1
Unique count	42,547	416,172	32
Thr = 0.3	Number of nodes	Number of edges	Number of relations
BRG	42,547	394,922	32
Train	4,747	8,770	1
Valid	2,544	3,172	1
Test	3,853	6,254	1
Unique count	42,547	413,118	32
Thr = 0.4	Number of nodes	Number of edges	Number of relations
BRG	42,547	392,429	32
Train	3,252	7,038	1
Valid	1,803	2,568	1
Test	2,601	5,029	1
Unique count	42,547	407,064	32
Thr = 0.5	Number of nodes	Number of edges	Number of relations
BRG	42,547	390,151	32
Train	2,597	5,540	1
Valid	1,412	2,004	1
Test	2,020	3,909	1
Unique count	42,547	398,834	32
Thr = 0.6	Number of nodes	Number of edges	Number of relations
BRG	42,547	386,705	32
Train	1,804	3,274	1
Valid	946	1,184	1
Test	1,367	2,279	1
Unique count	42,547	393,442	32
Thr = 0.7	Number of nodes	Number of edges	Number of relations
BRG	42,547	385,090	32
Train	846	2,137	1
Valid	556	764	1
Test	739	1,556	1
Unique count	42,547	389,547	32
Thr = 0.8	Number of nodes	Number of edges	Number of relations
BRG	42,547	384,900	32
Train	755	2,015	1
Valid	516	721	1
Test	664	1,466	1
Unique count	42,547	389,102	32

2.20 Supplementary Table 20

Supplementary Table 20: Summary of the dataset for the synthetic lethality prediction task (inductive setting): the **number of nodes, edges and relation types** in the background regulatory graph (BRG) as well as train, validation and test set obtained from SynLethDB-v2.0 for various thresholds.

	Number of nodes	Number of edges	Number of relations
Thr = 0.0			
BRG (training graph)	23,861	223,709	30
Train	2,265	3,314	1
Valid	2,267	3,319	1
BRG (inference graph)	20,096	156,446	27
Test	2,312	4,014	1
Unique count	30,187	373,582	30
Thr = 0.3			
BRG (training graph)	23,861	223,171	30
Train	1,877	2,873	1
Valid	1,883	2,876	1
BRG (inference graph)	20,096	156,175	27
Test	2,103	3,652	1
Unique count	30,187	371,527	30

2.21 Supplementary Table 21

Supplementary Table 21: **Best hyperparameter set for Synthetic lethality gene pair prediction task**, *dim* refers to embedding dimension, *L* is the number of hidden layers, *lr* is the learning rate, *Adv. T* the adversarial temperature used in self-adversarial negative sampling, *Aggr. Function* specifies the aggregation function.

**Model**	*dim*	*L*	*lr*	*NegSam*	*Adv. T*	*Aggr. Function*
BioPathNet (transductive setting)	32	5	5e-3	64	0.5	PNA
BioPathNet (inductive setting)	32	6	5e-3	128	0.5	PNA

2.22 Supplementary Table 22

Supplementary Table 22: Detailed breakdown of the relation types in the synthetic lethality data set (inductive setting): **Number of edges per relation type** in BRG, train, validation and test set.

	Relation	Count (thr = 0.0)	Count (thr = 0.3)
BRG (training graph)	involved in	62,838	62,838
	part of	38,896	38,896
	enables	32,625	32,625
	located in	30,572	30,572
	PARTICIPATES GpPW	24,554	24,554
	is a	19,378	19,378
	is active in	5,193	5,193
	SL GsG	4,442	3,904
	positively regulates	1,169	1,169
	negatively regulates	1,004	1,004
	regulates	923	923
	colocalizes with	533	533
	contributes to	485	485
	has part	302	302
	NOT|involved in	218	218
	NOT|enables	144	144
	acts upstream of or within	106	106
	acts upstream of	90	90
	occurs in	81	81
	NOT|part of	70	70
	NOT|located in	60	60
	acts upstream of positive effect	8	8
	acts upstream of or within positive effect	4	4
	NOT|colocalizes with	4	4
	happens during	3	4
	acts upstream of negative effect	2	2
	NOT|contributes to	2	2
	ends during	1	1
	NOT|acts upstream of or within	1	1
	NOT|acts upstream of or within negative effect	1	1
BRG (inference graph)	involved in	42,694	42,694
	part of	26,968	26,968
	enables	21,829	21,829
	located in	20,764	20,764
	PARTICIPATES GpPW	17,172	17,172
	is a	16,719	16,719
	is active in	3,547	3,547
	SL GsG	2,693	2,422
	positively regulates	1,005	1,005
	negatively regulates	814	814
	regulates	772	772
	colocalizes with	341	341
	contributes to	267	267
	has part	248	248
	NOT|involved in	158	158
	acts upstream of or within	96	96
	NOT|enables	89	89
	occurs in	76	76
	acts upstream of	66	66
	NOT|part of	63	63
	NOT|located in	48	48
	NOT|colocalizes with	5	5
	acts upstream of positive effect	4	4
	acts upstream of or within positive effect	3	3
	happens during	3	3
	ends during	1	1
	NOT|contributes to	1	1
Train	SL GsG	3,314	2,873
Valid	SL GsG	3,319	2,876
Test	SL GsG	4,014	3,652

2.23 Supplementary Table 23

Supplementary Table 23: Summary of the functional annotation data set: **number of nodes, edges and relation types** in the background regulatory graph (BRG), as well as train, validation and test sets.

	Number of nodes	Number of edges	Number of relations
BRG	15,806	1,035,133	7
Train	2,646	4,867	6
Valid	533	608	6
Test	535	609	6
Unique count	16,844	1,041,217	13

2.24 Supplementary Table 24

Supplementary Table 24: Detailed breakdown of the relation types in the functional annotation data set: **Number of edges per relation type** in BRG, train, validation and test set.

	Relation	Count
BRG	interacts-with	469,203
	in-complex-with	154,868
	controls-state-change-of	144,259
	catalysis-precedes	141,554
	controls-expression-of	114,698
	controls-phosphorylation-of	7,246
	controls-transport-of	3,305
Train	ceRNA or sponge	2,305
	expression association	1,530
	interact with protein	366
	transcriptional regulation	351
	epigenetic regulation	272
	interact with mRNA	43
Valid	ceRNA or sponge	275
	expression association	208
	interact with protein	48
	transcriptional regulation	44
	epigenetic regulation	31
	interact with mRNA	2
Test	ceRNA or sponge	247
	expression association	228
	interact with protein	52
	transcriptional regulation	47
	epigenetic regulation	28
	interact with mRNA	7

2.25 Supplementary Table 25

Supplementary Table 25: **Best hyperparameter set for LncRNA-gene target prediction task**, *dim* refers to embedding dimension, *L* is the number of hidden layers, *lr* is the learning rate, *Adv. T* the adversarial temperature used in self-adversarial negative sampling, *Aggr. Function* specifies the aggregation function.

**Model**	*dim*	*L*	*lr*	*NegSam*	*Adv. T*	*Aggr. Function*
BioPathNet	32	6	5e-3	64	0.5	PNA

3 Supplementary Figures

3.1 Supplementary Figure 1

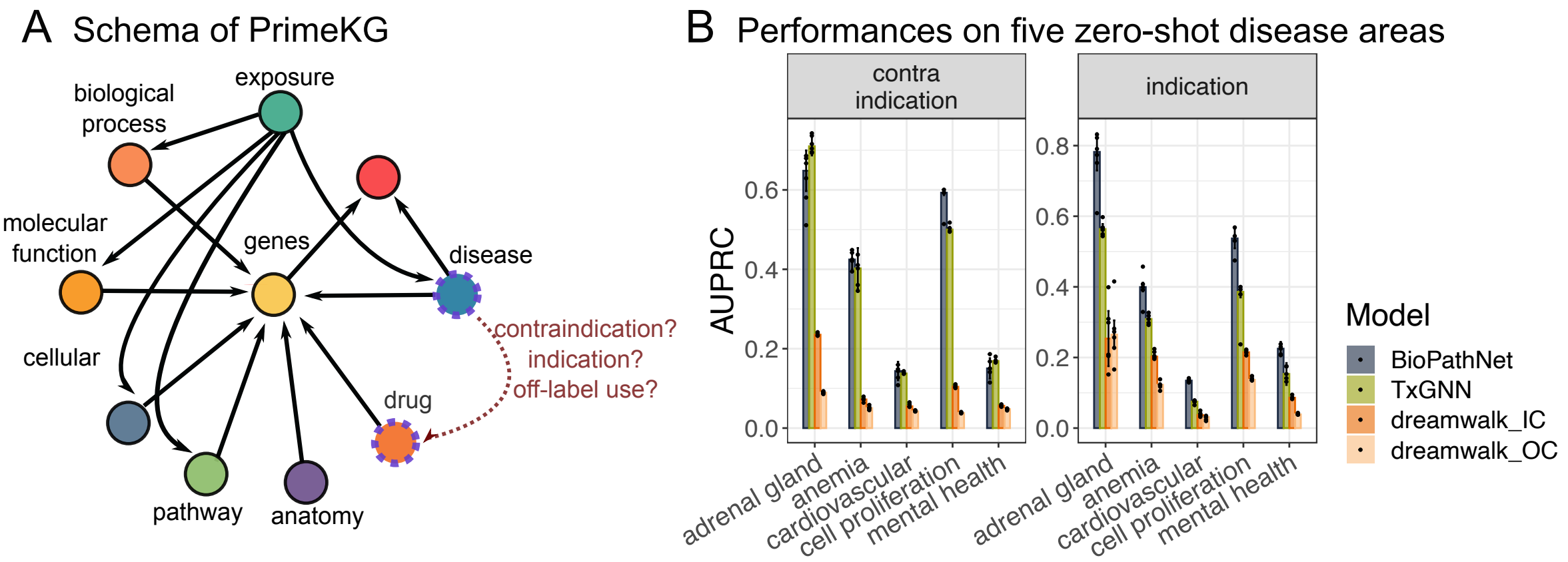

Supplementary Figure 1: **BioPathNet's performance on PrimeKG A**) PrimeKG schema: a multi-modal knowledge graph with 10 biological node types (e.g., protein, disease, drug) and over 5 million relations, for predicting drug-disease interactions. **B**) AUPRC performance in zero-shot prediction across five disease area splits (adrenal gland, anemia, cardiovascular, cell proliferation, mental health), calculated by comparing ground truth positives and negatives, then averaging across all diseases in each area. Performance is reported for BioPathNet, TxGNN [8], and DREAMWalk [9], using both inferred connections (IC) and original connections (OC) from PrimeKG [10].

3.2 Supplementary Figure 2

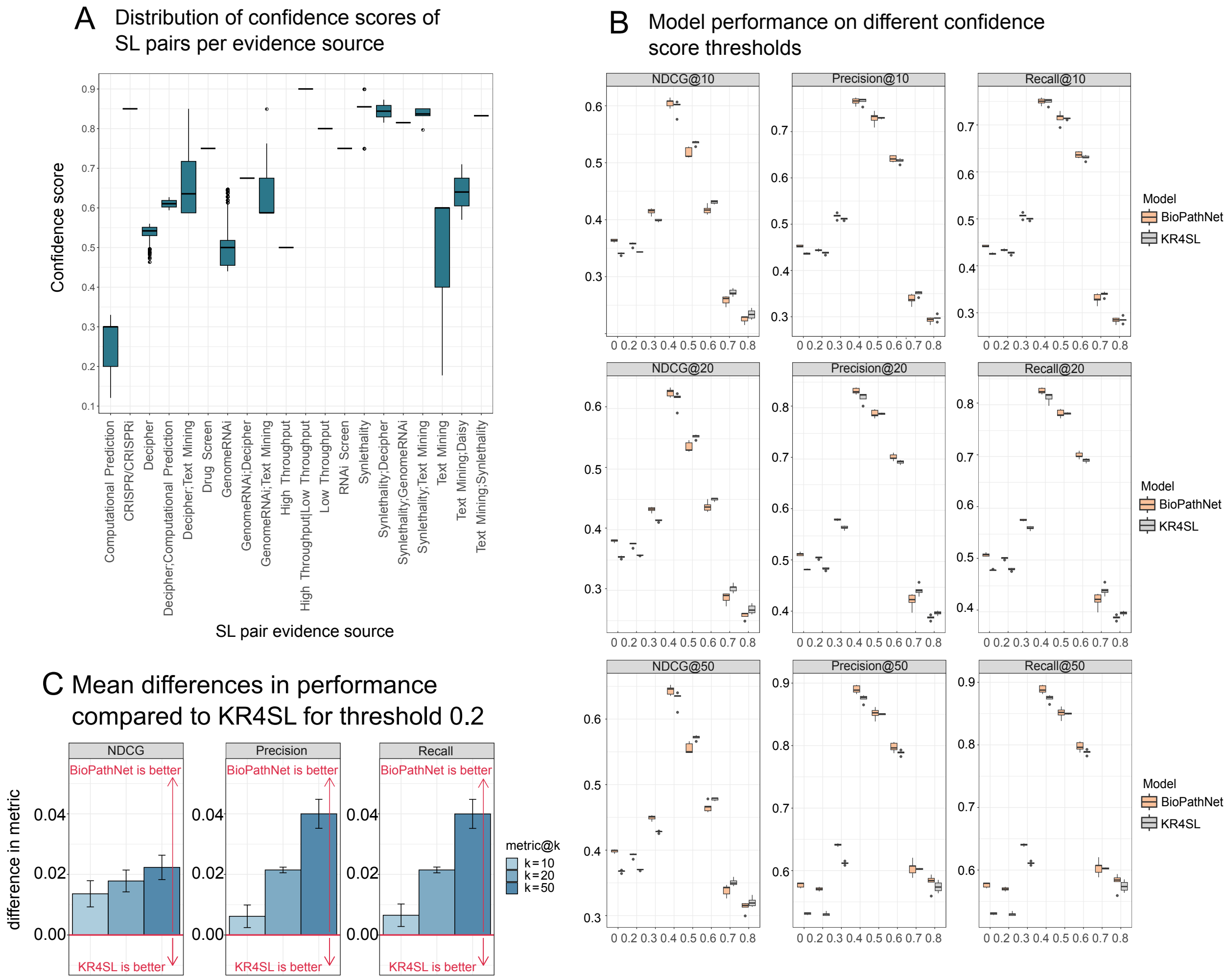

Supplementary Figure 2: **Comparison of BioPathNet with state-of-the-art SL gene pair prediction algorithm KR4SL for varying SL confidence thresholds: A**) Distribution of confidence scores of SL pairs per evidence source as provided by SynLethDB-v2.0 as the ”r.statistic_score”. **B**) Performance comparison of BioPathNet and KR4SL with performance metrics reported across thresholds applied to the confidence score of SL evidence. **C**) Difference in performances between BioPathNet and KR4SL for both methods trained on SL pairs which were filtered to have a confidence score of at least 0.2. Bars are shown for each metric (NDCG, Precision, Recall) and different k, and represent the mean difference in performance, while error bars denote standard deviations for the different seeds ($N = 5$).

3.3 Supplementary Figure 3

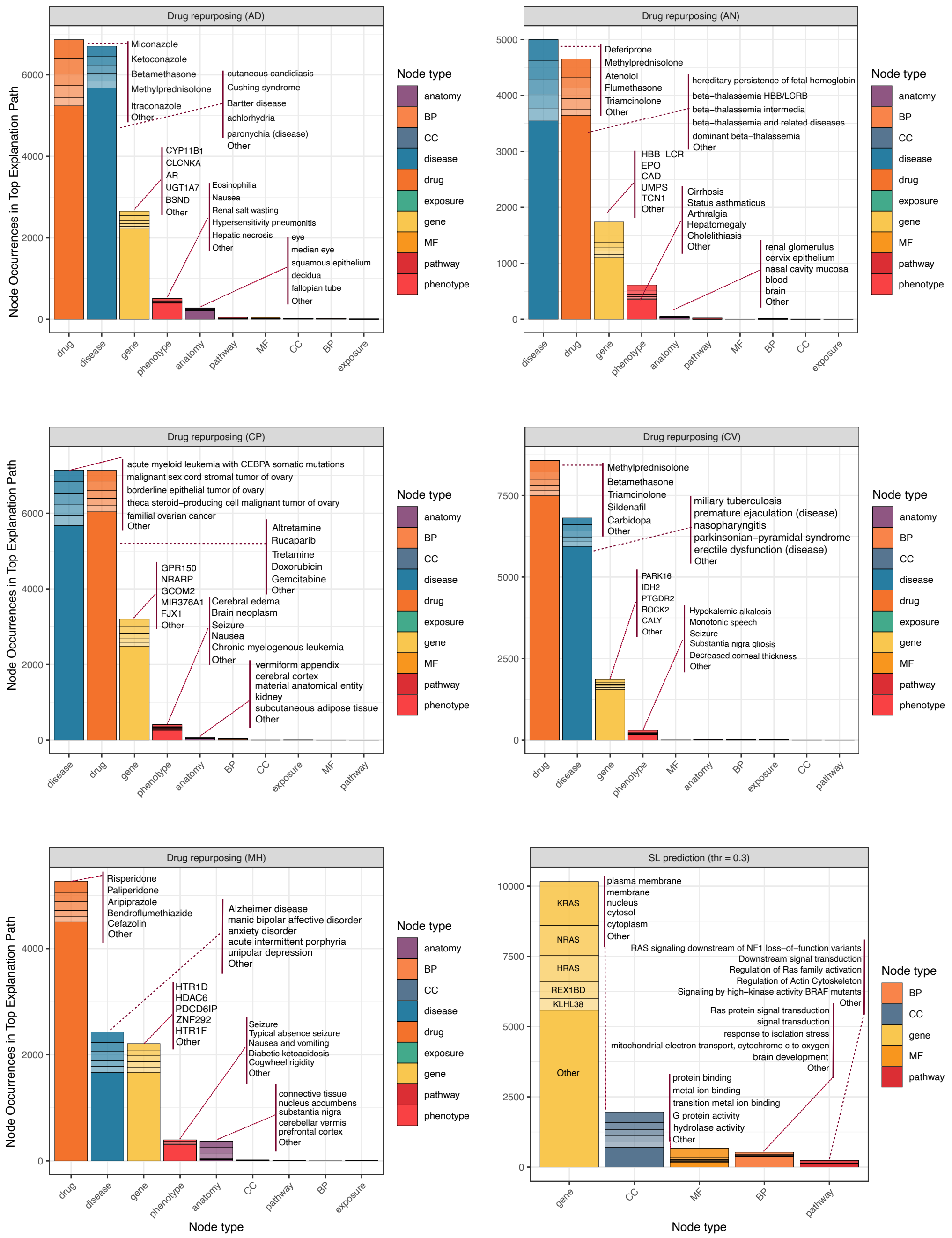

Supplementary Figure 3: **Most frequent nodes and node types in top explanation paths:** Bars show the frequency of node types appearing in the top 10 explanation paths for the top 50 predicted links (consistent across random seeds) for each prediction task: indication prediction for adrenal gland (AD), anemia (AN), cell proliferation (CP), cardiovascular (CV), and mental health (MH), as well as gene pair prediction for synthetic lethality (SL), at a confidence threshold of 0.3. Within each node type, the five most frequently occurring nodes are highlighted. The color gradient within each bar represents increasing node degree, allowing assessment of whether explanation paths are biased toward high-degree nodes.

3.4 Supplementary Figure 4

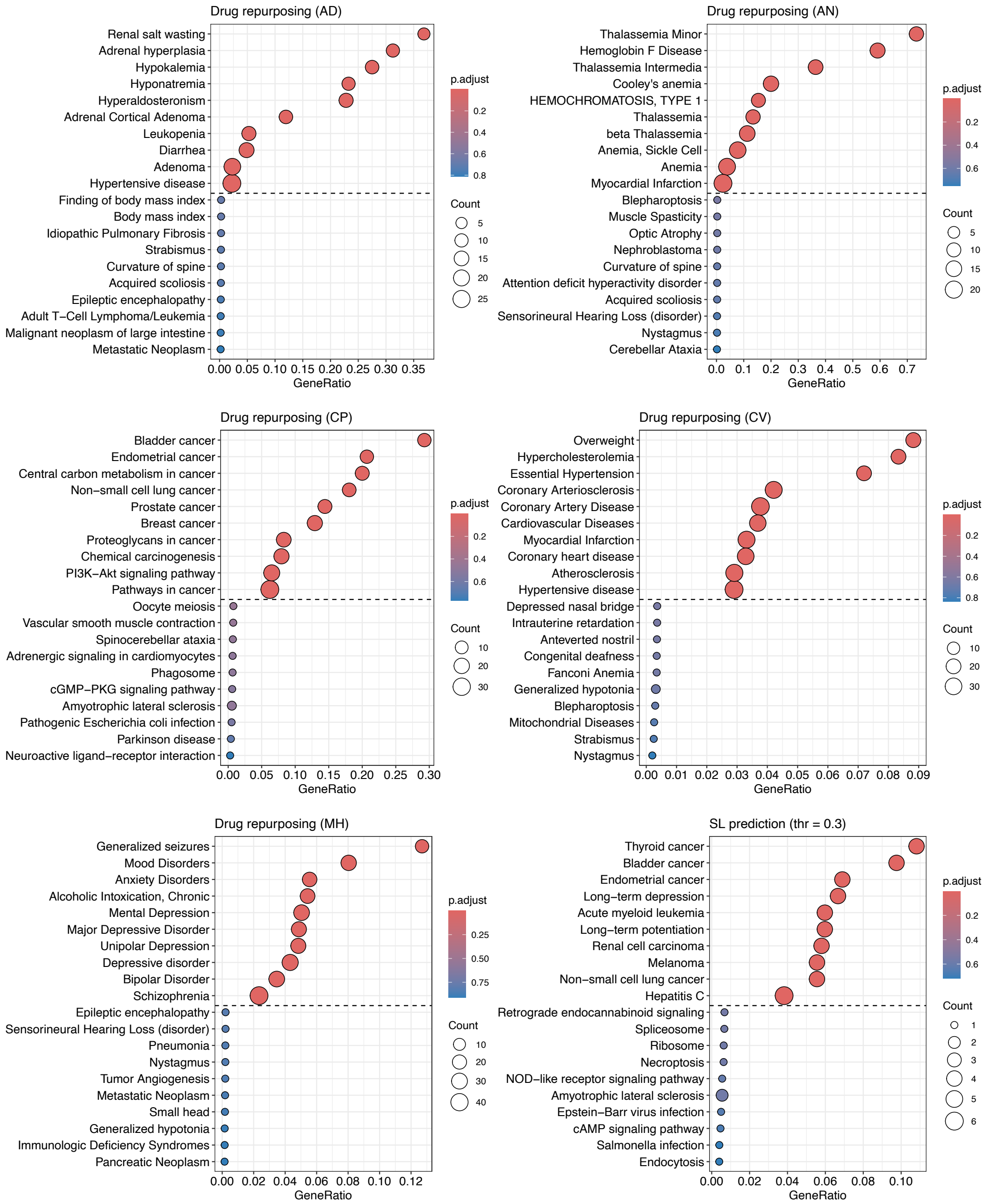

Supplementary Figure 4: **Gene enrichment analysis:** Gene enrichment analysis showing overrepresented DisGeNET terms for indication prediction across drug repurposing disease areas: adrenal gland (AD), anemia (AN), cardiovascular (CV), and mental health (MH); and KEGG pathway enrichment for synthetic lethality (SL; confidence score >= 0.3) and cell proliferation (CP). Terms were considered significantly enriched if the adjusted p-value (Benjamini–Hochberg correction) from Fisher's exact test (R package enrichR) was <= 0.01. Each panel displays the top 10 most enriched terms (red, top) and the 10 least enriched terms (control, bottom). The x-axis (GeneRatio) represents the ratio of genes from the top explanation paths that overlap with a given gene set, divided by the total size of that gene set.

3.5 Supplementary Figure 5

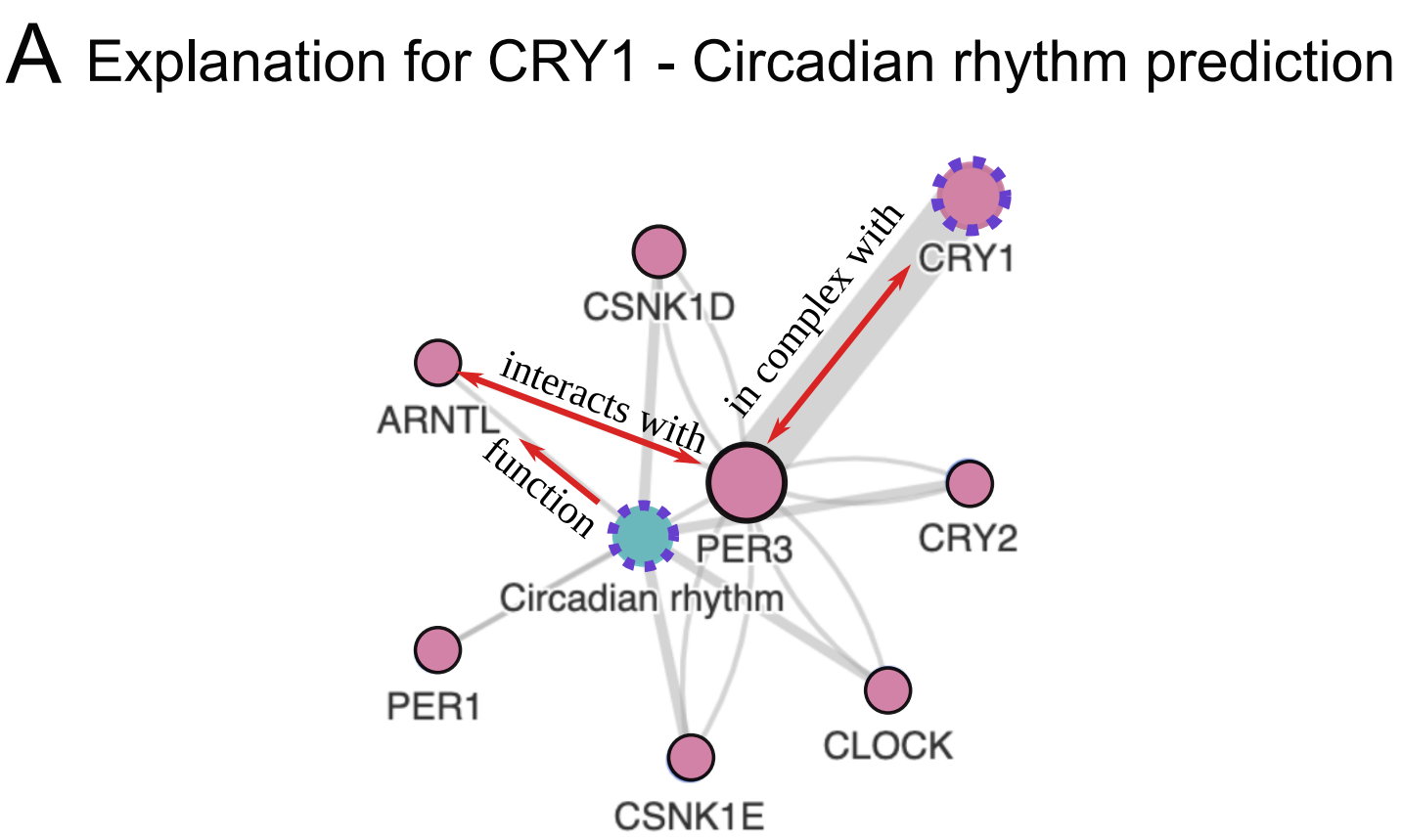

Supplementary Figure 5: **BioPathNet's explanations on Gene Function Prediction A**) The visualization highlights the significant paths employed by the BioPathNet model to predict a link between *CRY1* gene and the *Circadian rhythm*. The top 10 paths are depicted, where the width of each edge corresponds to its weight, and the path with the highest weight is highlighted in red.

3.6 Supplementary Figure 6

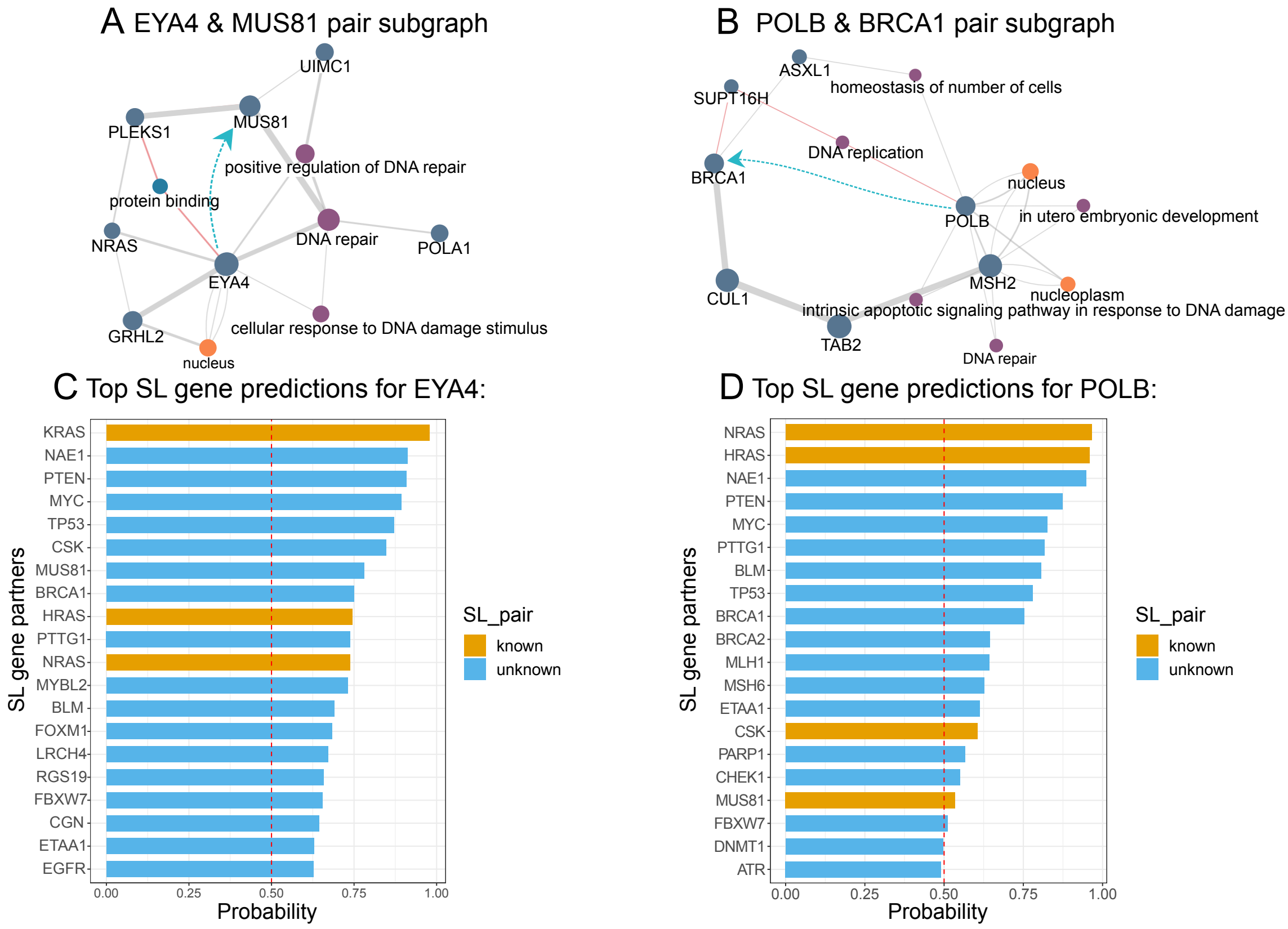

Supplementary Figure 6: **BioPathNet accurately uncovers cancer synthetic lethality gene pairs:** A) Visualization of gradients on paths important for the prediction of the *EYA4* - *MUS81* gene pair. B) Visualization of gradients on paths important for the prediction of the *POLB* - *BRCA1* gene pair. C) Top predicted SL gene partners for *EYA4*. D) Top predicted SL gene partners for *POLB*.

3.7 Supplementary Figure 7

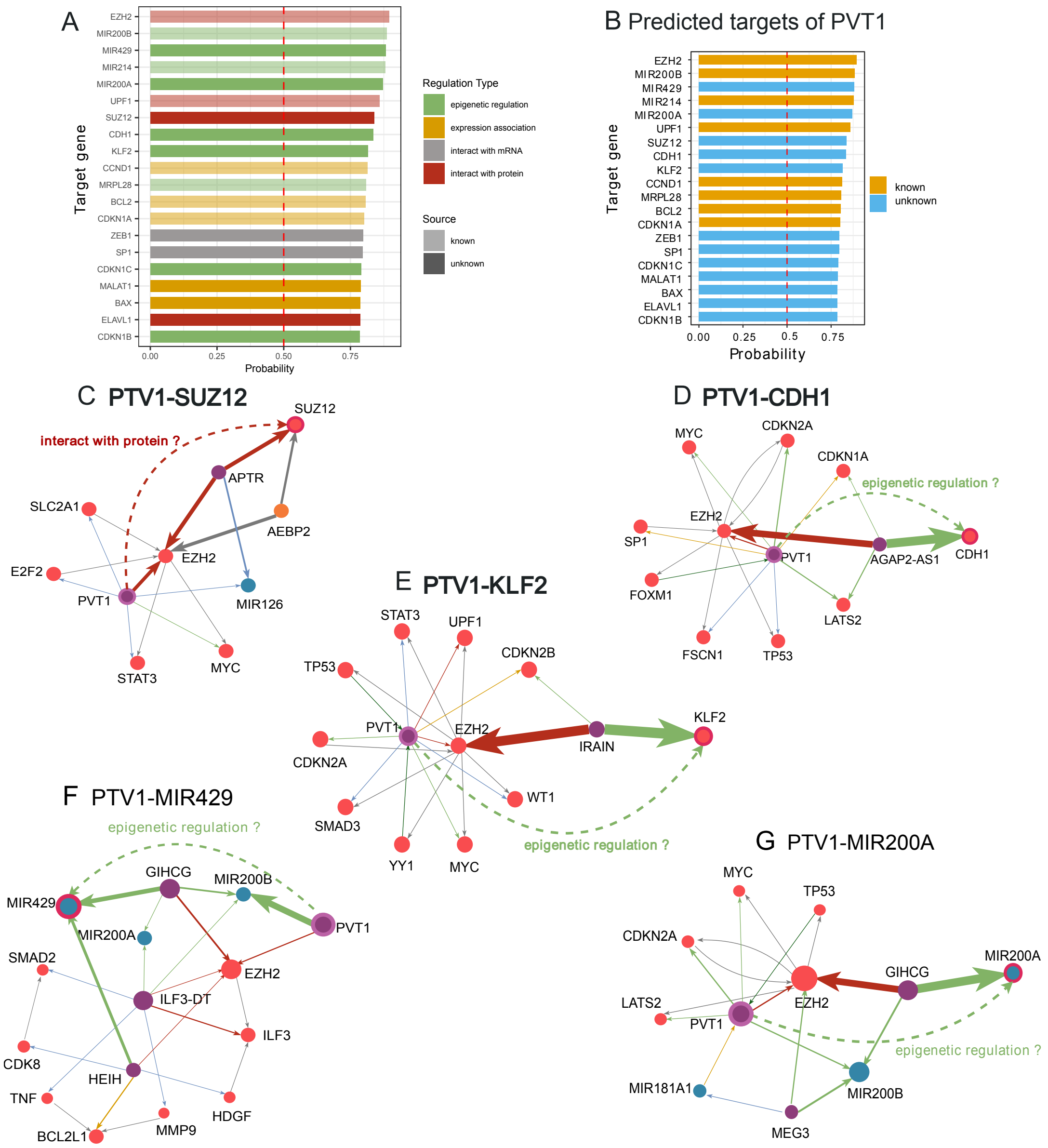

Supplementary Figure 7: **Prediction of novel lncRNA-target regulatory interactions. A** presents BioPathNet predicted targets for the cancer lncRNA PVT1, ranked by prediction probability. Additionally, regulation types are distinguished using different colors: green for epigenetic regulation, yellow for expression association, gray for interaction with mRNA, and red for interaction with protein. Known triples are shown with 50% transparency, while novel triples are displayed with full opacity. **B** BioPathNet predicted targets for the cancer lncRNA PVT1, ranked by prediction probability. Annotated targets are depicted in orange, while novel interacting partners are depicted in light blue.**C-G** Explanations for top 3-5 novel predicted targets of PVT1: SUZ12, CDH1, KLF2, MIR429 and MIR200A.

3.8 Supplementary Figure 8

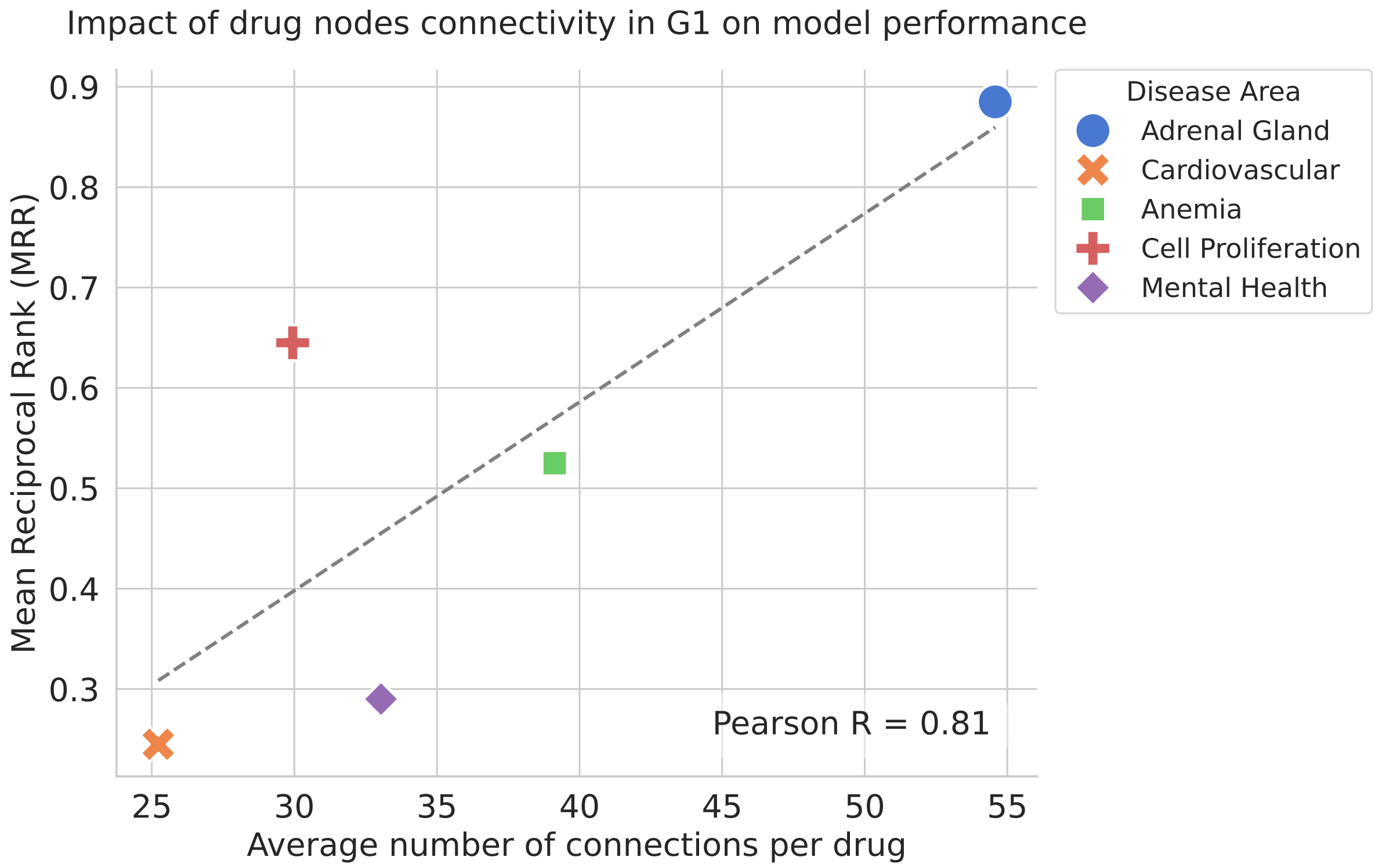

Supplementary Figure 8: **Impact of drug nodes connectivity on model performance:** Relationship between the average number of connections per drug in the G1 graph versus model's performance measured by the Mean Reciprocal Rank (MRR); pearson correlation of 0.81.

References

[1] Bang-Jensen, J., Gutin, G.: Section 2.3.4: The Bellman-Ford-Moore algorithm. Digraphs: Theory, Algorithms and Applications. Springer, London (2000)

[2] Baras, J.S., Theodorakopoulos, G.: Path problems in networks. Synth. Lect. Commun. Netw. **3**(1), 1–77 (2010)

[3] Zhu, Z., Zhang, Z., Xhonneux, L.-P., Tang, J.: Neural bellman-ford networks: A general graph neural network framework for link prediction. In: Advances in Neural Information Processing Systems, vol. 34, pp. 29476–29490. Curran Associates Inc., Virtual Event (2021)

[4] Corso, G., Cavalleri, L., Beaini, D., Liò, P., Veličković, P.: Principal neighbourhood aggregation for graph nets. Advances in Neural Information Processing Systems **33**, 13260–13271 (2020)

[5] A Python Library for Learning and Evaluating Knowledge Graph Embeddings. PyKEEN. https://github.com/pykeen/pykeen

[6] Glorot, X., Bengio, Y.: Understanding the difficulty of training deep feedforward neural networks. In: Teh, Y.W., Titterington, M. (eds.) Proceedings of the 13th International Conference on Artificial Intelligence and Statistics, pp. 249–256. PMLR, Chia Laguna Resort, Sardinia, Italy (2010)

[7] Bordes, A., Usunier, N., Garcia-Duran, A., Weston, J., Yakhnenko, O.: Translating embeddings for modeling multi-relational data. In: Advances in Neural Information Processing Systems, vol. 26. Curran Associates Inc., Lake Tahoe, NV, USA (2013)

[8] Huang, K., *et al.*: A foundation model for clinician-centered drug repurposing. Nat. Med. **30**(12), 3601–3613 (2024)

[9] Bang, D., Lim, S., Lee, S., S., K.: Biomedical knowledge graph learning for drug repurposing by extending guilt-by-association to multiple layers. Nat. Commun. **21**(1), 48 (2023)

[10] Chandak, P., Huang, K., Zitnik, M.: Building a knowledge graph to enable precision medicine. Sci. Data **10**(1), 67 (2023)
