# Supplementary material for: Enhancing link prediction in biomedical knowledge graphs with BioPathNet
Source: Nat Biomed Eng. 2026 Jan 20;10(8):1747–69. doi: 10.1038/s41551-025-01598-z (PMC13472943; doi:10.1038/s41551-025-01598-z)
Supplement: Supplementary file 2 [file 41551_2025_1598_MOESM2_ESM.pdf]

nature portfolio

Corresponding author(s): Annalisa Marsico
Last updated by author(s): 23/09/2025

Reporting Summary

Nature Portfolio wishes to improve the reproducibility of the work that we publish. This form provides structure for consistency and transparency in reporting. For further information on Nature Portfolio policies, see our Editorial Policies and the Editorial Policy Checklist.

Statistics

For all statistical analyses, confirm that the following items are present in the figure legend, table legend, main text, or Methods section.

n/a	Confirmed	
☐	☒	The exact sample size (*n*) for each experimental group/condition, given as a discrete number and unit of measurement
☒	☐	A statement on whether measurements were taken from distinct samples or whether the same sample was measured repeatedly
☒	☐	The statistical test(s) used AND whether they are one- or two-sided<br>*Only common tests should be described solely by name; describe more complex techniques in the Methods section.*
☒	☐	A description of all covariates tested
☒	☐	A description of any assumptions or corrections, such as tests of normality and adjustment for multiple comparisons
☐	☒	A full description of the statistical parameters including central tendency (e.g. means) or other basic estimates (e.g. regression coefficient) AND variation (e.g. standard deviation) or associated estimates of uncertainty (e.g. confidence intervals)
☒	☐	For null hypothesis testing, the test statistic (e.g. *F*, *t*, *r*) with confidence intervals, effect sizes, degrees of freedom and *P* value noted<br>*Give P values as exact values whenever suitable.*
☒	☐	For Bayesian analysis, information on the choice of priors and Markov chain Monte Carlo settings
☒	☐	For hierarchical and complex designs, identification of the appropriate level for tests and full reporting of outcomes
☒	☐	Estimates of effect sizes (e.g. Cohen's *d*, Pearson's *r*), indicating how they were calculated

Our web collection on statistics for biologists contains articles on many of the points above.

Software and code

Policy information about availability of computer code

Data collection	The following datasets were used for training, validation and testing of BioPathNet on different tasks.<br>Gene function prediction: we downloaded the background regulatory graph (BRG) of gene-gene and gene-chemical interactions from Pathway Commons (PC) in simple interaction format (node1, relationType, node2) from the file PathwayCommons12.All.hgnc.sif.gz (https://www.pathwaycommons.org/); functional annotations from the Kyoto Encyclopedia of Genes and Genomes (KEGG) were retrieved from ConsensusPathDB (CPDB_pathways_genes.tab file) using HGNC gene symbols (http://cpdb.molgen.mpg.de/).<br>Drug repurposing: the data split for five different disease areas, reflecting a zero-shot prediction scenario, and used for BioPathNet training, validation and testing (as explained in detail in the Method section) were downloaded from the TxGNN repository using the TxGNN code (https://github.com/mims-harvard/TxGNN from Apr 13, 2023, commit "1000aac"), and pre-processed as explained in Methods.<br>Synthetic lethality: for training and inference with BioPathNet, we used the SynLethDB-v2.0 data, as provided by KR4SL, and downloaded from their GitHub repository (https://github.com/JieZheng-ShanghaiTech/KR4SL from Dec 8, 2023 - commit "61b5c84")<br>lnRNA-target prediction: the BRG was downloaded from Pathway Common, while lncRNA-gene regulatory interactions were obtained from the https://lnctard.bio-database.com/ (lnctard2.0.gz file)<br>A bash script is provided in the repository (https://github.com/CogDisResLab/BioPathNet) to download all necessary data.<br>Custom code was used for data pre-processing as detailed in the Methods section.
Data analysis	The NBFNet code from the repository https://github.com/DeepGraphLearning/NBFNet was used as base, as subsequently modified, for our custom implementation of BioPathNet. This is based on torch and torchdrug (this last one is implemented internally at Mila https://github.com/DeepGraphLearning/torchdrug). Custom code was used for model augmentation of BioPathNet via a Background Regulatory Graph (BRG), and for performing node type-aware negative sampling, model training and model evaluation.<br>Link prediction files and corresponding explanations (the top 10 most important paths) were pre-processed using custom scripts and converted into HTML files. These files were then served through a static HTTP server (https://www.npmjs.com/package/http-server) to

generate the path interpretation plots shown in Figure 6 and Supplementary Figures 1, 4-5.
For comparison with baselines and other methods, the following repository, containing efficient current implementations of TransE, DistMul, RotatE and R-GNN (hosted at Mila), was used: https://github.com/DeepGraphLearning/torchdrug.
For HGT, custom implementation based on PyTorch Geometric (https://github.com/pyg-team/pytorch_geometric) was used.
For RAGAT, the following implementation was used: https://github.com/liuxiyang641/RAGAT.
For BIONIC, the following implementation was used: https://github.com/bowang-lab/BIONIC.
For TxGNN, the following implementation was used: https://github.com/mims-harvard/TxGNN.
For DREAMWalk, custom implementation was used.
For KR4SL, the following implementation was used: https://github.com/JieZheng-ShanghaiTech/KR4SL.
For DeepLGP, custom implementation in PyTorch that was converted from the original code (https://github.com/zty2009/LncRNA-target-gene) and modified was used.
Details on parameter tuning and training for both the baseline models and BioPathNet across various tasks are provided in the Methods section and in Supplementary File, Sections 1.2 to 1.6.

For manuscripts utilizing custom algorithms or software that are central to the research but not yet described in published literature, software must be made available to editors and reviewers. We strongly encourage code deposition in a community repository (e.g. GitHub). See the Nature Portfolio guidelines for submitting code & software for further information.

Data

Policy information about availability of data

All manuscripts must include a data availability statement. This statement should provide the following information, where applicable:

- Accession codes, unique identifiers, or web links for publicly available datasets
- A description of any restrictions on data availability
- For clinical datasets or third party data, please ensure that the statement adheres to our policy

Gene function prediction data is available from PathwayCommons and ConsensusDB (see Methods for details). For drug repurposing PrimeKG data and splits are downloaded via TxGNN. SynLethDB data, processed by KR4SL, was obtained from their GitHub, and lncRNA target prediction data from LncTarD 2.0. Further preprocessing was done to fit BioPathNet's data format, all scripts can be found in the repository https://github.com/emyyue/BioPathNet. A bash script is also provided in the repository to download all necessary data from the corresponding locations (https://github.com/emyyue/BioPathNet/blob/main/data/download.sh).

Research involving human participants, their data, or biological material

Policy information about studies with human participants or human data. See also policy information about sex, gender (identity/presentation), and sexual orientation and race, ethnicity and racism.

Reporting on sex and gender	not applicable
Reporting on race, ethnicity, or other socially relevant groupings	not applicable
Population characteristics	not applicable
Recruitment	not applicable
Ethics oversight	not applicable

Note that full information on the approval of the study protocol must also be provided in the manuscript.

Field-specific reporting

Please select the one below that is the best fit for your research. If you are not sure, read the appropriate sections before making your selection.

☒ Life sciences ☐ Behavioural & social sciences ☐ Ecological, evolutionary & environmental sciences

For a reference copy of the document with all sections, see nature.com/documents/nr-reporting-summary-flat.pdf

Life sciences study design

All studies must disclose on these points even when the disclosure is negative.

Sample size	Sample sizes, including number of network nodes used in the BRG, number of relations of different types, training, validation and test size, are reported, for each BioPathNet application, in supplementary tables 1-2 (gene function prediction), 4-5 (drug repurposing), 9-12 (synthetic lethality) and 18-19 (lncRNA -gene pair prediction).<br>Since we augment the original foreground graph with a background regulatory graph (BRG), the sample size of training, validation and test network is always sufficient to obtain accurate BioPathNet estimations of the predicted links.<br>Training, validation and test sets are chosen using label stratification (as specified for each single task in the Methods section), roughly in a 70%-10%-20% proportion.
Data exclusions	In the gene function prediction task, KEGG pathways with fewer than 10 gene annotations were excluded during data pre-processing.

Data exclusions	Additionally, 11 nodes from connected components with only 2-3 nodes were removed during graph construction. For the drug repurposing task, off-label use edges were excluded from TxGNN as we wanted to focus on drug indication and contraindication for diseases.<br>In both the drug repurposing and synthetic lethality tasks, reverse edges introduced by TxGNN and KR4SL were removed, as BioPathNet adds reverse edges by default.<br>In the lncRNA-target prediction task, firstly the gene names and gene types in LncTarD 2.0 were aligned to Ensembl human gene database, Homo_sapiens.GRCh38.110, with further removal of the genes that could not have a match in Ensembl human gene database. Additionally, the conflict of gene names and types between LncTarD 2.0 and the common human gene databases was manually resolved. Moreover, the pairs of relation type chromatin looping we re-labeled to other relation types as this relation is relatively rare (only 12 instances).
Replication	For the drug repurposing task, replication and robustness of performances and predictions is guaranteed by creating, for each disease area, 5 different disease splits (as explained in Methods), corresponding to five different seeds, to create training, validation and test. For gene function prediction, synthetic lethality prediction and lncRNA-mRNA target prediction, we run BioPathNet 5 times for 5 different random seeds.
Randomization	Randomization is garanteed by the use of five different seeds to run BioPathNet for each task.
Blinding	No blinding was used in this study.

Reporting for specific materials, systems and methods

We require information from authors about some types of materials, experimental systems and methods used in many studies. Here, indicate whether each material, system or method listed is relevant to your study. If you are not sure if a list item applies to your research, read the appropriate section before selecting a response.

Materials & experimental systems

n/a	Involved in the study	
☒	☐	Antibodies
☒	☐	Eukaryotic cell lines
☒	☐	Palaeontology and archaeology
☒	☐	Animals and other organisms
☒	☐	Clinical data
☒	☐	Dual use research of concern
☒	☐	Plants

Methods

n/a	Involved in the study	
☒	☐	ChIP-seq
☒	☐	Flow cytometry
☒	☐	MRI-based neuroimaging

Plants

Seed stocks	not applicable
Novel plant genotypes	not applicable
Authentication	not applicable
