# Supplementary material for: Enhancing link prediction in biomedical knowledge graphs with BioPathNet
Source: Nat Biomed Eng. 2026 Jan 20;10(8):1747–69. doi: 10.1038/s41551-025-01598-z (PMC13472943; doi:10.1038/s41551-025-01598-z)
Supplement: Supplementary file 3 [file 41551_2025_1598_MOESM3_ESM.pdf]

nature portfolio

Peer Review File

Enhancing link prediction in biomedical knowledge graphs with BioPathNet

Corresponding Author: Dr Annalisa Marsico

Version 0:

Decision Letter:

Dear Dr Marsico,

Thank you again for submitting to *Nature Biomedical Engineering* your manuscript, "BioPathNet: Enhancing Link Prediction in Biomedical Knowledge Graphs through Path Representation Learning". The manuscript has been seen by 3 experts, whose reports you will find at the end of this message.

You will see that the reviewers appreciate the work. However, they express concerns about the degree of support for the claims, and provide useful suggestions for improvement. We hope that with substantial further work you can address the criticisms and convince the reviewers of the merits of the study. In particular, we would expect that a revised version of the manuscript provides:

* Performance comparisons with more appropriate baselines.

* Assessment of the performance contributions of using node-type-aware negative sampling and of relational graphs, and of any potential biases arising from them.

* A clearer write-up, with thorough methodology.

When you are ready to resubmit your manuscript, please upload the revised files, a point-by-point rebuttal to the comments from all reviewers, the reporting summary, and a cover letter that explains the main improvements included in the revision and responds to any points highlighted in this decision.

Please follow the following recommendations:

* Clearly highlight any amendments to the text and figures to help the reviewers and editors find and understand the changes (yet keep in mind that excessive marking can hinder readability).

* If you and your co-authors disagree with a criticism, provide the arguments to the reviewer (optionally, indicate the relevant points in the cover letter).

* If a criticism or suggestion is not addressed, please indicate so in the rebuttal to the reviewer comments and explain the reason(s).

* Consider including responses to any criticisms raised by more than one reviewer at the beginning of the rebuttal, in a section addressed to all reviewers.

* The rebuttal should include the reviewer comments in point-by-point format (please note that we provide all reviewers will the reports as they appear at the end of this message).

* Provide the rebuttal to the reviewer comments and the cover letter as separate files.

We expect that you will be able to resubmit the manuscript within 16 weeks of receiving this message. If this is the case, you will be protected against potential scooping. Otherwise, we will be happy to consider a revised manuscript as long as the significance of the work is not compromised by work published elsewhere or accepted for publication at *Nature Biomedical Engineering*.

We hope that you will find the referee reports helpful when revising the work. Please do not hesitate to contact me should you have any questions.

Best wishes,

Pep

__
Pep Pàmies
Chief Editor, *Nature Biomedical Engineering*

____
Reviewer #1 (Report for the authors (Required)):

In this paper, the authors present BioPathNet, a deep learning framework designed to improve link prediction in biomedical knowledge graphs through path representation learning (Neural Bellman-Ford Network; NBFNet) and the integration of a Background Regulatory Graph (BRG). The authors claim that path-based reasoning in NBFNet is more applicable for capturing complex biomedical relationships compared to traditional node embedding-based approaches that are limited to one-hop connections. BioPathNet enhances the link prediction performance over complex knowledge graph by learning representations over multi-hop paths between node pairs, allowing for richer relational understanding within the knowledge graph. In addition, authors used a regulatory network as a background, as referred to as the Background Regulatory Graph, which allows the NBFNet to learn a more diverse context of the biomedical knowledge graph. Augmented with the node type-aware negative sampling strategy, the BioPathNet outperforms existing biomedical knowledge graph link prediction methods in four tasks. Authors provide performance comparison with the state-of-the-art models in gene function annotation, drug-disease indication prediction, synthetic lethality prediction, and lncRNA-mRNA interaction prediction, along with comprehensive case studies to support the interpretability of the path-based approach. By identifying influential paths, the model demonstrates its interpretability of molecular mechanisms underlying the predicted associations.

Key results reported in the manuscript are:
1. In the task of gene function prediction, BioPathNet significantly outperformed node embedding-based methods, such as TransE and RotatE, as well as relational GCNs (R-GCNs).

2. In the drug repurposing task, the model was applied to predict drug-disease associations in a zero-shot setting, showing a consistent advantage over the state-of-the-art GNN-based model TxGNN. BioPathNet's performance gains, particularly in prediction precision for novel diseases, were notable. Some of the identified potential drug-disease indications were validated by medical experts and existing literature.

3. In predicting synthetic lethal gene pairs, BioPathNet outperformed KR4SL, an existing path-based GNN model. BioPathNet's better handling of noisy data from synthetic lethality gene databases showed promising results for cancer research.

4. The model was also applied to uncover regulatory relationships between long non-coding RNAs (lncRNAs) and their gene targets, outperforming both node embedding and GNN-based methods. BioPathNet's ability to accurately predict novel interactions was validated by external datasets from CRISPR-based experiments.
We interpret BioPathNet as a promising approach of applying path-based learning framework, NBFNet, to biomedical knowledge graph by considering two hurdles of: (1) heterogeneity of the network through the node type-aware negative sampling, and (2) allowing flexible integration of external knowledge bases with background regulatory network.

Major comments
Our major concerns are provided below.

1. The authors claim that a path-based model like NBFNet is more applicable than embedding-based approaches that consider only one-hop connections. However, relatively naïve traditional models such as RotatE and TransE are selected as baselines to compare against BioPathNet. Intensive comparative experiments with recently proposed embedding-based models that demonstrate state-of-the-art performance in link prediction tasks on biomedical knowledge graphs is necessary. Some of these models consider beyond one-hop relations and integrate contextual information e.g. [DREAMwalk, DeepMPF, MHTAN-DTI]

2. This study applies NBFNet to Biomedical Knowledge Graphs and incorporates additional entity and relation information, referred to as the Biomedical Relational Graph (BRG), for message passing. Among the four tasks used to evaluate the performance of BioPathNet, two tasks—gene-function prediction and lncRNA-gene target prediction—brings Pathway Commons, an external database, as the BRG.

The remaining two tasks—drug repurposing and synthetic lethality prediction—use the unused edges (excluding the ones used in supervised training) as the BRG. However, the ablation study supporting the impact of BRG is provided only for the gene-function prediction task. Although all tasks are related to link prediction problem, the complexity and problem settings

of the tasks are different in terms of dataset composition, specific training methods applying BRG, and levels of inference difficulty. Thus, it is needed to present separate experimental results for each task to support the claim that the BRG contributes to improving the model's performance.

Moreover, we think the use of problem domain-specific networks seems reasonable. However, it is important to note that our current knowledge on any biological entity is incomplete. Thus, bringing in domain specific networks may result in significant bias from our current knowledge. Potential bias due to the application of BRGs, based on the characteristics of the origin knowledge base, should be thoroughly investigated.

Lastly, we believe that a single comprehensive and universal knowledge graph could serve as a background for all four tasks. Can this be achieved with the BRG concept? We believe achieving such a goal will improve the utility of the BRG concept in using a general background knowledge augment generated paths.

3. Use of negative sampling to utilize class-independent algorithms such as Bellman-Ford algorithm seems a good idea. It is naturally aligned to the authors claim that this stringent node type-aware negative sampling scheme ensures contrastive learning and improves decision boundary accuracy. We find this approach novel. However, although the authors' claim that they demonstrated the effectiveness of this novel negative sampling strategy, there is a lack of analysis on the impact of negative sampling on performance. The negative samples have a critical role in determining decision boundaries. This should be thoroughly investigated to further confirm the strengths of the approach compared to naive sampling that considers the network homogeneously.

Minor comments
1. At the time of this paper's submission, TxGNN was available as a preprint on arXiv. Since then, TxGNN has been published in Nature Medicine. We kindly ask the authors to verify whether there have been any significant updates or changes in the final version of the TxGNN model that could impact the comparative evaluation or relevance of BioPathNet's approach. (citation below)

2. The 'adversarial temperature value' defined by the authors needs more clearance. Is it a sampling ratio, or an annealing parameter? In addition, the adversarial temperature for negative sampling was kept constant throughout some of the experiments. Considering that performance can vary depending on the hyperparameters, the authors need to demonstrate on how and why the chosen value is optimal.

3. If a low negative sampling ratio is enough to achieve better performance than existing models, I think this can be considered a strength of the framework. Our recommendation is to put more emphasis on this point.

References
[DREAMwalk] Bang, D., Lim, S., Lee, S. et al. Biomedical knowledge graph learning for drug repurposing by extending guilt-by-association to multiple layers. Nat Commun 14, 3570 (2023). https://doi.org/10.1038/s41467-023-39301-y

[DeepMPF] Ren, Zhong-Hao, et al. "DeepMPF: deep learning framework for predicting drug–target interactions based on multi-modal representation with meta-path semantic analysis." Journal of Translational Medicine 21.1 (2023): 48.

[MHTAN-DTI] Zhang, Ran, et al. "MHTAN-DTI: Metapath-based hierarchical transformer and attention network for drug–target interaction prediction." Briefings in Bioinformatics 24.2 (2023): bbad079.

[TxGNN] Huang, K., Chandak, P., Wang, Q. et al. A foundation model for clinician-centered drug repurposing. Nat Med (2024). https://doi.org/10.1038/s41591-024-03233-x

Reviewer #2 (Report for the authors (Required)):

Link prediction (LP) is a fundamental task in AI for biomedicine. While existing embedding-based methods have shown promising results in predicting potential links in biomedical knowledge graphs (KGs), some limitations remain, such as the lack of interpretability. To address these challenges, the authors proposed BioPathNet, a graph neural network framework to enhance link prediction in KGs. As opposed to the node-embedding frameworks, BioPathNet learns representations of target biological pairs based on relations along the path. Moreover, the authors introduced an external biological regulatory graph (BRG) and a negative sampling strategy to further improve the performance of BioPathNet. To evaluate its performance, BioPathNet is compared with several baselines across four link prediction tasks. Experimental results show that BioPathNet achieves overall better performance than the baselines and also provides interpretable results. While this study shows promising results of the proposed AI method in the biomedical LP tasks, there are still several critical issues that need to be addressed.

Major Comments:
1. One of the important technical designs in BioPathNet is the negative sampling strategy (in line 158). However, the authors have not explained the goal of contrastive learning or how it is used, making it difficult to understand the design of the negative sampling strategy.

2. Compared with the backbone NBFNet, BioPathNet introduces an external BRG and a negative sampling strategy to

improve performance. Introducing an external BRG provides more biological information for the model to learn from, thus leading to better performance. However, it also creates an unfair comparison for baselines without BRG, including task-specific baselines and NBFNet. To address this, the authors should conduct experiments with baselines (including NBFNet) that incorporate BRG as well, similar to the setup in the first task of predicting gene-functional associations.

3. In comparison to NBFNet, the current work lacks a systematic study across the link prediction tasks to clearly demonstrate the effectiveness of the BRG and the negative sampling strategy. As a result, the technical differences between BioPathNet and NBFNet remain ambiguous. Ablation studies are recommended.

4. In the Results part, some of the chosen baselines are somewhat outdated, which weakens the persuasiveness of the comparison results. Despite the promising performance of BioPathNet, the conclusions may differ when incorporating more recent GNN models, such as GIN [1], HGT [2], RAGAT [3], and INDIGO [4].

5. The authors compare task-specific methods for the drug repurposing and synthetic lethality (SL) prediction tasks, but no such comparison is made for the gene function prediction and lncRNA-target interaction tasks. Including task-specific baseline models for the two LP tasks would strengthen the comprehensiveness of the study, making the conclusion more convincing.

6. Due to the incompleteness of KG data, it's better to show the performance of BioPathNet under the inductive settings, particularly since models like KR4SL and NBFNet have demonstrated the performance in the inductive scenarios.

7. The computational efficiency of models is a critical factor in practical applications. The authors should present relevant efficiency metrics, such as training time.

8. It is too early to claim that BioPathNet identifies some novel SL pairs, just relying on the evidence from the explanation subgraphs. Wet-lab biological experiments would be needed for that.

9. Why would the path-based framework be suitable for the 4 LP tasks? Please explain it from perspective of data, e.g. distribution of edges in the graphs.

10. To what extend is the performance of the method dependent on the completeness and quality of the KG and BRG? This is important for the practicality of the proposed method.

Minor Comments:
1. The writing can be more succinct and focused. For instance, Subsection 5.1 looks redundant and too general. The title of Subsection 5.5 looks vague and lack focus. Overall, the manuscript reads a bit verbose. If the authors have used any LLM (like GPT) to generate some of the text, please disclose it.

2. In Introduction, the authors claim to address the "link prediction in noisy biological knowledge graphs (KGs)" in line 147, but the definition of "noisy" is missing from the context. The authors should clarify this point to avoid confusion.

3. In line 149, "the node-embedding learning frameworks that optimize the embedding space based on one-hop relations" is confusing. Following [1], GNNs broadly follow a message passing scheme, where each node aggregates feature vectors of its neighbors to compute its new embeddings. After k iterations of aggregation, the final node embeddings capture the structural information within the node's k-hop neighborhood, not one-hop.

4. Providing an overview diagram of the model could enhance the reader's understanding of BioPathNet's architecture.

5. In line 97, the juxtaposition of "knowledge graphs (KGs)" and "heterogeneous multi-relational networks" seems odd, since KGs are a specific case of heterogeneous multi-relational networks.

6. There are some typos in Supplementary Table 7. For instance, the "AUPRC of 0.605" is labeled in bold but is actually the second-best result. Similar typos can be found in the "AUPRC 0.57".

7. The icons of the models are hard to distinguish in Supplementary Figure 3C.

References:
[1] Xu K, Hu W, Leskovec J, et al. How Powerful are Graph Neural Networks? International Conference on Learning Representations. 2018.
[2] Hu Z, Dong Y, Wang K, et al. Heterogeneous graph transformer. Proceedings of the web conference 2020. 2020: 2704-2710.
[3] X. Liu, H. Tan, Q. Chen and G. Lin. RAGAT: Relation Aware Graph Attention Network for Knowledge Graph Completion. IEEE Access, vol. 9, pp. 20840-20849, 2021.
[4] Liu S, Grau B, Horrocks I, et al. INDIGO: GNN-based inductive knowledge graph completion using pair-wise encoding. Advances in Neural Information Processing Systems, 2021, 34: 2034-2045.

The code provided is comprehensive, and the introduction to using BioPathNet is clear and easy to understand.

Reviewer #3 (Report for the authors (Required)):

The paper introduces BioPathNet, a novel graph neural network framework designed to enhance link prediction in biomedical knowledge graphs through path representation learning. The authors leverage the Neural Bellman-Ford Network (NBFNet) to combine path-based reasoning with a Background Regulatory Graph (BRG), resulting in improved accuracy and interpretability. BioPathNet was validated across several tasks such as gene function annotation, drug repurposing, and synthetic lethality prediction, demonstrating strong performance compared to state-of-the-art methods. Despite its promising contributions, several methodological concerns and areas for improvement should be addressed.

In general, the paper is difficult to read and understand. Part of this is not the fault of the authors because Results before Methods is confusing. The methods do not describe where BRG comes from and how it works and so this is a total confusing element of the paper (there are a few sentences in 5.3 and as far as I can tell, nothing else). Also, it is not clear whether the primary contributions here are the new link-prediction method or the applications. So the paper kind of goes back and forth featuring the link-prediction vs. focusing on the application results. The application results are all over the place and so hard to digest the biological strengths of the method. The general message seems to be “it can do everything” but there is not deep evaluation of this implicit claim, but instead a few examples from different areas of biomedicine which are interesting but don’t give confidence about overall performance. It may be that the authors should present one paper with the computer science method of link prediction, and then one or more application papers focusing on specific tasks and validating the performance against other methods. As it is, there is (for me) too much.

Points of Concern:
1. Node-type Aware Negative Sampling: The use of node-type aware negative sampling is a step forward, but it could introduce bias by over-representing certain node types and neglecting others, especially in imbalanced biological datasets. This might lead to suboptimal performance on rare or underrepresented node pairs, requiring more careful handling of data imbalance across various types of nodes. We suggest implementing methods that can dynamically adjusts the selection of negative samples based on graph density. This would help balance the distribution of node types and ensure that the model does not overfit to certain dense regions while ignoring sparser sections of the graph.

2. BRG and Data Leakage: How the BRG is incorporated into the model should be more clearly described. There is limited discussion of the role of the BRG in training and inference and so the reader is left uncertain about the impact these edges have on the prediction made. The paper would benefit from a more detailed explanation of how the BRG is incorporated during both the training and prediction phases. It remains unclear whether the BRG is used consistently or differently at these stages, which could have implications for the model’s generalization and performance.

It is not clearly stated if the edges present in the BRG (G2) overlap with the predicted edges. This could artificially inflate performance metrics, and it is important to ensure that the model does not predict relationships that are already present in G2 during training. Furthermore, the assumptions about what type of network is used as the BRG are not clear. If the BRG includes noisy or missing data, as is common in biological networks, techniques such as graph regularization or uncertainty estimation should be considered.

3. Baseline Selection Discussion: The selection of baseline models, while including common approaches, lacks thorough justification. The baselines chosen address generalized link-prediction tasks, but do not specifically provide points of comparison for link prediction in biological networks. A more in-depth discussion of why certain baselines were chosen, and what novel aspect of the proposed model creates the increased performance would be helpful.

Version 1:

Decision Letter:

Dear Dr Marsico,

Thank you again for submitting to *Nature Biomedical Engineering* your manuscript, "BioPathNet: Enhancing Link Prediction in Biomedical Knowledge Graphs through Path Representation Learning". The manuscript has been seen by the 3 previous experts, whose reports you will find at the end of this message.

There are relatively minor comments to address, and if you're able to address them and resubmit we will be happy to move forward with publication of the paper.

When you are ready to resubmit your manuscript, please upload the revised files, a point-by-point rebuttal to the comments from all reviewers, the reporting summary, and a cover letter that explains the main improvements included in the revision and responds to any points highlighted in this decision.

Please follow the following recommendations:

*Please submit your revised manuscript in a word doc or LaTeX format.

* Clearly highlight any amendments to the text and figures to help the reviewers and editors find and understand the changes (yet keep in mind that excessive marking can hinder readability).

* If you and your co-authors disagree with a criticism, provide the arguments to the reviewer (optionally, indicate the relevant points in the cover letter).

* If a criticism or suggestion is not addressed, please indicate so in the rebuttal to the reviewer comments and explain the reason(s).

* Consider including responses to any criticisms raised by more than one reviewer at the beginning of the rebuttal, in a section addressed to all reviewers.

* The rebuttal should include the reviewer comments in point-by-point format (please note that we provide all reviewers will the reports as they appear at the end of this message).

* Provide the rebuttal to the reviewer comments and the cover letter as separate files.

We expect that you will be able to resubmit the manuscript within 25 weeks of receiving this message. If this is the case, you will be protected against potential scooping. Otherwise, we will be happy to consider a revised manuscript as long as the significance of the work is not compromised by work published elsewhere or accepted for publication at *Nature Biomedical Engineering*.

We hope that you will find the referee reports helpful when revising the work. Please do not hesitate to contact me should you have any questions.

Best wishes,

Barbara Cheifet
Editor
Nature Biomedical Engineering

Reviewer #1 (Report for the authors (Required)):

I read and reviewed the revised manuscript and the authors' rebuttal letter, and found that the revised manuscript improved significantly, reflecting my concerns satisfactorily.

1. Task-specific Baseline Comparisons: The authors expanded the baseline models for performance comparison: DREAMwalk, RAGAT, HGT, BIONIC, DeepLGP, and KR4SL. Their efforts to adapt and reproduce these methods seem to be thorough, and the revised comparisons give a much clearer picture of where BioPathNet stands in the current landscape.
2. Ablation of the Background Regulatory Graph (BRG): They added ablation studies for all four tasks to better show the role of the BRG. The experiments help clarify its effect on both performance and computational cost. I also appreciated the additional discussion on potential biases from domain-specific graphs, as well as the supporting perturbation analyses.
3. Effectiveness of the Proposed Negative Sampling: The new results on their node-type-aware (NTA) negative sampling method are helpful to understand the contributions of the proposed study. The performance gains are significant across most tasks, and the updated figures and tables make this point clearly.
4. Generalizability of the BRG: The authors also discussed about the generalizability of the BRG. They highlight limitations due to variation in task-specific knowledge graphs, and their preliminary results support the idea that tailored graphs may still be needed for better performance.

Overall, I find the revised manuscript improved significantly. The additional experiments and clarifications contribute to improving the transparency and rigor of the work. The authors' responses are satisfactory, and I have no further major concerns.

Reviewer #1 (Remarks on code availability):

Codes for training the proposed model along with notebooks for reproduction of figure plots are provided in the GitHub repo, with well documented README file on setup and training procedures.

Reviewer #2 (Report for the authors (Required)):

The authors have done a good job in improving the manuscript significantly.

Reviewer #3 (Report for the authors (Required)):

Summary of Revisions
The authors have made substantial improvements to the manuscript in response to the previous review. Notably:
• Expanded baseline comparisons: The outdated baselines (TransE, RotatE, DistMult) were replaced with seven more recent and appropriate models, including RAGAT, HGT, R-GCN, the original NBFNet, and multiple task-specific baselines (DreamWalk, KR4SL, BIONIC, lncBIONIC, DeepLGP).
• Ablation studies were conducted for each of the four link prediction (LP) tasks to evaluate the contributions of the background regulatory graph (BRG) and node-type-aware negative sampling (NTA), now also including structure-aware negative sampling (SANS) as per our earlier suggestion.
• Bias and robustness analysis: The authors assessed model bias with respect to node degree and tested robustness to edge deletions, noise, and removal of relation types, providing insight into BRG design trade-offs.
• Clarification and documentation: Key methodological choices, such as adversarial temperature, negative sampling ratios, and the justification for excluding some baselines, have been explicitly clarified.

Major Concerns
1. The authors emphasize that BioPathNet is more interpretable than standard node-embedding models because it reasons over paths and allows visualization of which multi-hop paths contribute to a prediction. While this is promising, the evidence is anecdotal — they include a few examples of predicted paths and claim biological plausibility, but there is no quantitative measure of interpretability, such as how often the top-ranked paths align with known biological pathways or mechanisms.
2. In the drug repurposing task, BioPathNet is evaluated across multiple disease areas in a zero-shot setting. However, performance (e.g., MRR) varies dramatically. For adrenal gland or cell proliferation, performance is strong (MRR > 0.6–0.8). For mental health and cardiovascular, MRR is much lower (~0.23–0.36). The paper does not discuss possible reasons for this inconsistency.
3. SynLethDB-v2.0 includes experimental and predicted SL pairs, but the authors did not clearly state which type of edges were included. Were predicted edges used in the training set? How was label noise from the predicted pairs handled?
Minor Concerns
1. The number of negatives per positive and how this ratio is selected or tuned is not clearly summarized in the main text or methods.
2. The value of k used for k-hop neighborhood in structure-aware sampling is mentioned only indirectly in the rebutta but is not explicitly reported in the manuscript.

Reviewer #3 (Remarks on code availability):

I don't review code. You would have to pay me a consulting fee! ;)

Version 2:

Decision Letter:

Dear Dr Marsico,

Thank you for your revised manuscript, "BioPathNet: Enhancing Link Prediction in Biomedical Knowledge Graphs through Path Representation Learning". Having consulted with CHOOSE_AND_CUSTOMIZE the original reviewers OR Reviewers #X and #Y and one more expert (whose comments you will find at the end of this message), I am pleased to write that we shall be happy to publish the manuscript in *Nature Biomedical Engineering*.

We will be performing detailed checks on your manuscript, and in due course will send you a checklist detailing our editorial and formatting requirements. You will need to follow these instructions before you upload the final manuscript files.

Please do not hesitate to contact me if you have any questions.

Best wishes,

Barbara Cheifet
Editor
Nature Biomedical Engineering

Reviewer #3 (Report for the authors (Required)):

We are satisfied with the responses to our concerns and have no more comments.

Thanks to the authors for being responsive.

Version 3:

Decision Letter:

Dear Dr Marsico,

I am happy to inform you that your manuscript, "Enhancing link prediction in biomedical knowledge graphs with BioPathNet", has now been accepted for publication in *Nature Biomedical Engineering*.

Over the next few weeks, the figures will be checked for production quality, the text edited to ensure that it conforms to house style, and the manuscript typeset.

Our Articles are published about 40 days after the acceptance date (we recommend that you inform your institutional press office of this timeframe), and you will be notified of the actual publication date a few days in advance. Articles can be published any working day of the week, and are pushed live shortly after 10 am London time.

Publishing agreement. You will be asked to digitally sign a publishing agreement (grant of rights). After the signed publishing agreement has been received, the proofs of the article will be sent to you for review. If you have any queries during the production process, or you cannot meet the requested deadline for returning the proofs, please contact rjsproduction@springernature.com.

Nature Biomedical Engineering is a Transformative Journal. Authors may publish their research with us through the traditional subscription access route, or make their paper immediately open access through payment of an article-processing charge. More information about publication options is available.

You may need to take specific actions to comply with funder and institutional open-access mandates. If the work described in the accepted manuscript is supported by a funder that requires immediate open access (as outlined, for example, by Plan S) and your manuscript was originally submitted on or after January 1st 2021, then you should select the gold OA route. Authors selecting subscription publication will need to accept our standard licensing terms (including our self-archiving policies), and these will supersede any other terms that the author or any third party may assert apply to any version of the manuscript.

Acceptance of your manuscript is conditional on agreement, by all authors, with both our media embargo and confidentiality and pre-publicity policies. In particular, you may arrange your own publicity of the Article (for instance, through your institutional press office), as long as you ensure that journalists strictly adhere to the media embargo.

To assist you in disseminating the work, as soon as the Article is published you will be able to take advantage of the Springer Nature SharedIt initiative to generate a unique shareable link to the Article that will allow anyone (with or without a subscription) to read it. Recipients of the link who are subscribers will also be able to download and print the PDF.

Thank you for having submitted this work to *Nature Biomedical Engineering*.

Best wishes,

Barbara Cheifet
Editor
Nature Biomedical Engineering

Open Access This Peer Review File is licensed under a Creative Commons Attribution 4.0 International License, which permits use, sharing, adaptation, distribution and reproduction in any medium or format, as long as you give appropriate credit to the original author(s) and the source, provide a link to the Creative Commons license, and indicate if changes were made.
In cases where reviewers are anonymous, credit should be given to 'Anonymous Referee' and the source.
The images or other third party material in this Peer Review File are included in the article's Creative Commons license, unless indicated otherwise in a credit line to the material. If material is not included in the article's Creative Commons license and your intended use is not permitted by statutory regulation or exceeds the permitted use, you will need to obtain permission directly from the copyright holder.
To view a copy of this license, visit https://creativecommons.org/licenses/by/4.0/

REBUTTAL LETTER

We sincerely appreciate the time and effort of the reviewers and the editor in evaluating our manuscript. Their insightful feedback has greatly improved the clarity, rigor, and impact of our work. We have carefully addressed all comments and believe the revised version strengthens the manuscript significantly.

As requested by all three reviewers, we have added performance comparisons with more appropriate baselines. We have removed previous baselines TransE, RotatE and DistMult and added 7 new additional baselines. These include: (1) more expressive general LP methods, such as RAGAT (Relation-Aware Graph Attention Network), HGT (Heterogeneous Graph Transformer), and the original NBFNet, along with Relational Graph Convolutional Networks (R-GCNs). While we had previously tested R-GCNs on the gene function task, we have now extended this evaluation to all tasks; and (2) task-specific models, besides TxGNN, including DreamWalk (in two settings) for drug-disease indication prediction, BIONIC (in two settings) for gene function prediction and lncRNA-gene pair predictions (lncBIONIC), KR4SL (previously used only in transductive, now including also inductive setting, per reviewer 2's request) for synthetic lethality, and DeepLGP for lncRNA target prediction.
We also want to highlight that adapting task-specific models required extensive coding to align them with our framework (or align our data to their framework). Additionally, some models could not be benchmarked due to usability issues, such as missing documentation for the data or code. Further details are provided in our individual responses to the reviewers below.

Following the reviewers' suggestions, we systematically evaluated the impact of node-type-aware negative sampling (NTA) and the background regulatory graph (BRG) on BioPathNet's performance. We conducted ablation studies across all tasks, removing one design choice at a time, using five seeds per task. Results are shown in Figure 2 (E-F) and detailed in Supplementary Tables 21-25. Our findings indicate that both heterogeneous negative sampling and BRG integration consistently enhance performance (with very few exceptions) — sometimes substantially.

Additionally, based on Reviewer 3's suggestion, we implemented structure-aware negative sampling (SANS) (Ahrabian et al., https://arxiv.org/pdf/2009.11355), which restricts negative samples to a node's k-hop neighborhood using random walks. This approach makes learning more challenging by sampling from regions of similar density, mitigating the bias of uniform negative sampling, which favors denser graph areas and weakens generalization in sparser regions. To systematically assess its impact, we conducted an ablation study on both NTA and SANS across all four link prediction tasks. The results, presented in Figure 2E and Supplementary Table 21-24, show that in most cases, NTA improves BioPathNet's performance. However, the best results — at least in the majority of cases — were achieved when combining NTA and SANS. While in some instances, the improvements were substantial, in others, they were modest, and in a very few cases, performance remained unchanged or declined.

While we acknowledge that combining SANS with NTA improves performance for some tasks, we conducted perturbation experiments and benchmarks in the rest of the paper using NTA alone. SANS's random walk-based sampling is computationally expensive, making a full re-run time-consuming within the timeframe of this revision. Since BioPathNet already performs well with NTA alone (see responses below) and to ensure consistent negative sampling across experiments and baselines (see *Comparison with Baselines*), we opted for this approach. Our SANS implementation is available in the BioPathNet GitHub repository, providing a resource for future applications in biomedical graph analysis.

To address potential biases in the predictive models and the use of different knowledge graphs, we first examined whether performance biases arise from predicted triplets involving high-degree nodes, which is a very common bias in link prediction methods Aiyappa et al. (https://arxiv.org/pdf/2405.14985). By stratifying MRR performance based on the head node degree, we assess (Figure 3A) how prediction probability that the model exhibits a bias toward node degree. Additionally, we systematically assess how performance is affected if we remove certain relation types from the knowledge graph, randomly delete edges at varying percentages, or introduce random edges (noise) into the KG, allowing us to also assess the quality of the different knowledge graphs. The results of these experiments are presented in Figure 3B and Section *BioPathNet's performance and evaluation of design choices.*

As suggested by the reviewers and editor, we have refined our writing to be more focused and concise, emphasizing BioPathNet's primary contributions. We expanded explanations in *Results* and *Methods*, detailing the BRG, its role in each task, model architecture, and key concepts like adversarial temperature and node-type-aware negative sampling. We also clarified BioPathNet's key advantages over NBFNet, specifically its biomedical graph–tailored design choices. We conducted additional experiments to demonstrate BioPathNet's generalizability and effectiveness in biomedical link prediction. The revised manuscript prioritizes methodology, ablation studies, bias and noise assessments, and benchmarks, while significantly shortening the biological application section. Methodological evaluation is now distinct from application results, with biological case studies moved to the end of *Results* (Figure 6).

These changes led to substantial revisions and figure rearrangements:

- **Figure 1**: Updated to illustrate NTA and SANS negative sampling.
- **Figure 2**: New, illustrating LP tasks and ablation study results.
- **Figure 3**: Reports node-degree bias, edge perturbation, and relation-type experiments.
- **Figure 4**: Benchmarks against general LP methods.
- **Figure 5**: Benchmarks against task-specific baselines.
- **Figure 6**: Showcases link prediction examples and interpretability.
- **Figure 7 (Methods)**: Illustrates model architecture and message passing.

We hope these changes help streamline the manuscript's main message, enhance clarity and coherence, and effectively highlight the method's effectiveness.

Reviewer 1

In this paper, the authors present BioPathNet, a deep learning framework designed to improve link prediction in biomedical knowledge graphs through path representation learning (Neural Bellman-Ford Network; NBFNet) and the integration of a Background Regulatory Graph (BRG). The authors claim that path-based reasoning in NBFNet is more applicable for capturing complex biomedical relationships compared to traditional node embedding-based approaches that are limited to one-hop connections. BioPathNet enhances the link prediction performance over complex knowledge graph by learning representations over multi-hop paths between node pairs, allowing for richer relational understanding within the knowledge graph. In addition, authors used a regulatory network as a background, as referred to as the Background Regulatory Graph, which allows the NBFNet to learn a more diverse context of the biomedical knowledge graph. Augmented with the node type-aware negative sampling strategy, the BioPathNet outperforms existing biomedical knowledge graph link prediction methods in four tasks. Authors provide performance comparison with the state-of-the-art models in gene function annotation, drug-disease indication prediction, synthetic lethality prediction, and lncRNA-mRNA interaction prediction, along with comprehensive case studies to support the interpretability of the path-based approach. By identifying influential paths, the model demonstrates its interpretability of molecular mechanisms underlying the predicted associations.

Key results reported in the manuscript are:

1. In the task of gene function prediction, BioPathNet significantly outperformed node embedding-based methods, such as TransE and RotatE, as well as relational GCNs (R-GCNs).

2. In the drug repurposing task, the model was applied to predict drug-disease associations in a zero-shot setting, showing a consistent advantage over the state-of-the-art GNN-based model TxGNN. BioPathNet's performance gains, particularly in prediction precision for novel diseases, were notable. Some of the identified potential drug-disease indications were validated by medical experts and existing literature.

3. In predicting synthetic lethal gene pairs, BioPathNet outperformed KR4SL, an existing path-based GNN model. BioPathNet's better handling of noisy data from synthetic lethality gene databases showed promising results for cancer research.

4. The model was also applied to uncover regulatory relationships between long non-coding RNAs (lncRNAs) and their gene targets, outperforming both node embedding and GNN-based methods. BioPathNet's ability to accurately predict novel interactions was validated by external datasets from CRISPR-based experiments.

We interpret BioPathNet as a promising approach of applying path-based learning framework, NBFNet, to biomedical knowledge graph by considering two hurdles of: (1) heterogeneity of the network through the node type-aware negative sampling, and (2)

allowing flexible integration of external knowledge bases with background regulatory network.

We thank the reviewer for this positive assessment.

<u>Major comments:</u>

1. The authors claim that a path-based model like NBFNet is more applicable than embedding-based approaches that consider only one-hop connections. However, relatively naïve traditional models such as RotatE and TransE are selected as baselines to compare against BioPathNet. **Intensive comparative experiments** with recently proposed embedding-based models that demonstrate state-of-the-art performance in link prediction tasks on biomedical knowledge graphs is necessary. **Some of these models consider beyond one-hop relations and integrate contextual information e.g. [DREAMwalk, DeepMPF, MHTAN-DTI**]

We agree that models like RotatE, TransE, and DistMult are relatively simple, with more advanced approaches now available. However, we initially selected them for their knowledge graph specificity, task generalizability, and suitability for illustrating the benefits of path embeddings over node embeddings. Additionally, simpler models can sometimes match or outperform complex ones while being more computationally efficient. Our goal was to assess whether this holds true or not and to justify the use of a sophisticated model like BioPathNet.

In response to the feedback, we have removed those methods from the comparison. While we retained R-GCN for its expressive ability in mining heterogeneous graphs (and as the foundation of the TxGNN method), and we now also evaluated it on other tasks, other than gene function prediction, we significantly expanded the number of baselines included in the paper, as outlined above. In detail, we also compare to RAGAT (Relation-Aware Graph Attention Network), HGT (Heterogeneous Graph Transformer), and the original NBFNet on all four tasks (task-agnostic baselines suggested by reviewer 2), with DreamWalk (in two settings) and TxGNN for drug-disease indication prediction, BIONIC (in two settings) for gene function and lncRNA-gene pair predictions, KR4SL (in both transductive and inductive settings) for synthetic lethality, and DeepLGP for lncRNA-target prediction.

We appreciate the reviewer's suggestion of alternative baselines. These models are highly task-specific. DeepMPF and MHTAN-DTI, for instance, are valuable tools but are specifically designed for drug-target interaction (DTI) prediction, with many hard-coded components tailored to that task. While we acknowledge those recent drug-gene interaction methods in the introduction, their task-specific nature and, unfortunately, lack of reproducible code or data description prevented benchmarking them against BioPathNet. Thus, we decided to focus on DreamWalk, as this is a recent method tailored to predict drug-disease indication prediction from biomedical knowledge graphs, making it more straightforward to compare to BioPathNet and

TxGNN . However, in this case as well, we had to make substantial coding modifications to successfully run the method.

The DREAMWalk algorithm repurposes drugs by exploring associations via multi-layer random walks. Its hard-coded hierarchical biomedKG integrates fixed components: a drug-drug and disease-disease similarity network, along with links connecting drugs and diseases to a PPI network. By leveraging semantic information and Guilt-by-Association across layers, the model identifies expressive paths in the network. Random walks generate embedding vectors using a heterogeneous Skip-gram model, which are then fed into an XGBoost classifier to predict drug-disease associations and repurposing probabilities.
The authors do not provide the underlying DREAMWalk graph needed for training, which in the original paper is constructed from the ATC hierarchy for drugs and the MeSH Ontology for diseases. While they outline their graph construction process, replicating it is time-consuming and beyond this paper's scope. Thus, we reconstructed its key components in two different ways to ensure comparability with TxGNN and BioPathNet. In the first experiment, we mapped DrugBank IDs from PrimeKG to the ATC hierarchy and used the DREAMWalk code to compute drug-drug semantic similarity. For diseases, we leveraged the MONDO hierarchy (which includes MeSH) in PrimeKG to compute semantic similarity. Finally, we integrated protein-protein interactions (PPIs), gene-protein, and protein-disease interactions from PrimeKG. We call this variant of DREAMWalk DreamWalk_IC (Inferred Connections). In a second experiment, instead of constructing the disease and drug graphs using semantic similarity from the DreamWalk algorithm, we directly extract these connections from PrimeKG. This allows us to evaluate whether this approach improves DREAMWalk's performance. We refer to this variant as DREAMWalk_OC (Original Connections). We used DreamWalk's default parameters to generate random walks from the graph, which were then used to create embedding vectors. These vectors map entities into a shared embedding space, which an XGBoost classifier then utilizes to predict drug-disease associations. The classifier was trained on the same triples (train and validation set) used for BioPathNet and TxGNN and performance was computed on the same test set.

This is now included in the Result's subsection *BioPathNet versus task-specific baselines* in *Comparison with baselines*, and more methodological details on the DREAMWalk framework are given in Extended Methods (Supplementary File).

2. This study applies NBFNet to Biomedical Knowledge Graphs and incorporates additional entity and relation information, referred to as the Biomedical Relational Graph (BRG), for message passing. Among the four tasks used to evaluate the performance of BioPathNet, two tasks—gene-function prediction and lncRNA-gene target prediction—brings Pathway Commons, an external database, as the BRG.
The remaining two tasks—drug repurposing and synthetic lethality prediction—use the unused edges (excluding the ones used in supervised training) as the BRG. However, **the ablation study supporting the impact of BRG is provided only for the gene-function prediction task.** Although all tasks are related to link prediction problem, the complexity and

problem settings of the tasks are different in terms of dataset composition, specific training methods applying BRG, and levels of inference difficulty.

a. **Thus, it is needed to present separate experimental results for each task to support the claim that the BRG contributes to improving the model's performance.**

We thank the reviewer for this comment and completely agree on the importance of demonstrating the impact of the BRG across the diverse link prediction tasks examined in this study. We conducted systematic ablation studies of the BRG across all four tasks, including all disease indication sub-tasks, using five different seeds. The results, shown in Figure 2F, illustrate the percentage change in BioPathNet's performance compared to its BRG-ablated version. Detailed performance values for all tasks, with and without BRG ablation, are provided in Supplementary Table 25.

The results are also described in *BioPathNet's performance and evaluation of design choices*, Results section. Using a BRG for message passing on top of the training graph from G1 KG improved performance across all tasks, with gains of 1.5–6.3% for lncRNA-mRNA target prediction, 16–24% for gene-function prediction, and up to 84% for synthetic lethality. However, in the zero-shot drug-repurposing task — where a 'new' disease is simulated by removing known treatments and similar diseases — removing the BRG severely disrupted connectivity, leaving the new 'diseases' nearly unconnected, preventing meaningful path-based predictions and causing a sharp performance drop. For fairness, we also ran a BRG ablation in a non-zero-shot setting with randomly split data. However, as noted by TxGNN's authors, this setup is too simplistic due to leakage from similar diseases in the training graph (G1), and it is not useful in practical application, making drug-disease prediction a trivial task and therefore leading to the BRG having no impact on the prediction performance (data not shown).

Beyond improving prediction performance, the use of BRG for message passing only significantly reduces computational time — sometimes drastically — compared to training on the full training graph from G1 + BRG, especially for large KGs like those in gene function prediction and drug repurposing, which can contain up to 5 million edges (see new Figure 4B for a computational time assessment). For example, for drug repurposing and with the PrimeKG, without splitting the KG into G1 for training and BRG for message passing, link prediction becomes computationally infeasible. This is crucial, as while NBFNet and path-embedding methods are highly accurate, they are neither practical nor sustainable without this design in comparison to GNN-based methods. Making such methods accessible for large-scale biomedical KGs, is one of the key, if not the most important, advantages of BioPathNet, which we now emphasize more clearly in the paper.

b. Moreover, we think the use of problem domain-specific networks seems reasonable. However, it is important to note that our current knowledge on any biological entity is incomplete. Thus, bringing in domain specific networks may result in significant bias from our current knowledge. **Potential bias due to the application of BRGs, based on the characteristics of the origin knowledge base, should be thoroughly investigated.**

We acknowledge that our understanding of biological entities and their connections remains incomplete and noisy and that domain-specific networks can introduce biases based on existing knowledge. While fully addressing all biases is challenging, we analyzed key factors influencing predictions, revealing how knowledge sources, graph quality, and structural differences impact link prediction.
Biological networks, particularly PPIs, exhibit *study bias*, where well-studied proteins — especially disease-related ones — have more recorded interactions, leading to overrepresentation of known pathways. To assess this, we examined bias toward high-degree nodes, a known issue in link prediction. Models tend to favor well-connected nodes, making them statistically more likely to form new links. This can overemphasize hubs, reducing generalization and performance for low-degree node predictions.

By computing and visualizing MRR stratified by node degree across all tasks (Figure 3A), we find no correlation between performance and query node degree for three of the four tasks — gene function prediction, synthetic lethality, and lncRNA — suggesting no bias toward genes, lncRNAs, or pathway hubs. However, for drug repurposing, we observe a weak positive correlation (0.23), indicating a tendency of the model to associate drug indications with well-characterized drugs or diseases.

We next assessed the impact on BioPathNet performance (measured by the MRR) of introducing random noise into the BRG by either adding random edges (10–80%) or removing edges at varying proportions (10–80%), as well as removing the top 5% highest-degree nodes. Additionally, we investigated whether specific relation types contribute more to performance or if BioPathNet primarily benefits from the overall connectivity provided by the BRG. To test this, we removed the top five relation types (based on edge count) for each task and evaluated performance changes. Due to the computational cost of these perturbation experiments we could not perform these analyses for every single relation, therefore we restricted it to the top 5. The results, summarized in a new figure, Figure 3B, show that the effects of these perturbations vary by task and depend on the BRG's density and quality, and that not all relations are equally important, with some leading to a decrease in performance when corresponding edges are depleted but other being redundant or, in some cases, detrimental to link prediction. Finally, across all tasks, we observe a substantial performance drop when removing the top 5% of high-degree nodes from the BRG. This aligns with expectations, as predictive paths are more likely to traverse network hubs.

Overall, our analysis underscores that connectivity between the training graph from G1 and G2 is more critical for performance than individual relation types, and that BioPathNet is able to find alternative paths, when certain edges are deleted, as long as the connectivity of the graph is not heavily affected. In fact, for each task, there is a negative correlation between shortest path's lengths in G1 and performance, highlighting that disrupting connectivity is the key. By conducting the first ablation study on relation types in heterogeneous biomedical KGs, we reveal that certain relations are redundant or even detrimental to prediction tasks. These

findings highlight the future need for improved strategies to assess and denoise biomedical knowledge graphs.

The results of this analysis are described in subsection 'BioPathNet's performance and evaluation of design choices', Results section and partly in the paper Discussion.

Lastly, we believe that a single comprehensive and universal knowledge graph could serve as a background for all four tasks. Can this be achieved with the BRG concept? We believe achieving such a goal will improve the utility of the BRG concept in using a general background knowledge augment generated paths.

We understand the reviewer's perspective that a general model for biomedical link prediction — essentially a foundation model capable of handling multiple tasks with the same KG or BRG — seems ideal. However, while we aim here at a generally applicable method, we believe different link prediction tasks may not necessarily benefit from this approach. Our experiments suggest that: (1) constructing a 'universal' KG requires a detailed analysis of various knowledge graphs from different projects, to understand what a universal KG could really be, which is a big task in itself. The landscape of biomedical KGs is complex, with diverse resources and pipelines for their construction, requiring significant effort to navigate and optimize them in the future. (2) preliminary results indicate that specific tasks perform better with dedicated KGs or BRGs. For example, replacing the synthetic lethality's BRG (SL pairs + GO graph) with the gene function's BRG (PathwayCommons, primarily PPI links) resulted in comparable or lower MRR (0.35 for thresholded data, 0.298 for unthresholded data; data not shown) compared to using the original task-specific BRG. Similarly, substituting the PrimeKG portion of the BRG with PathwayCommons led to a significant performance drop in drug repurposing, as the latter lacks drug-gene connections, severely limiting the available paths between training graph from G1 and BRG. As noted by Lobentanzer et al. (https://www.nature.com/articles/s41587-023-01848-y), building task-specific KGs is crucial, as a generalized biomedical knowledge representation may not suit diverse research tasks (as our preliminary experiments showed). If tasks rely on different background knowledge and benefit from distinct prior information, a unified approach may not be necessary. For instance, drug repurposing requires a different KG structure than gene function prediction, which benefits from PPI networks and gene-metabolite relationships within specific pathways. The recent preprint about the KGWAS method (Huang et al., medRxiv) leverages known variant-to-gene, gene-to-gene and gene-to-pathway links to build a task specific knowledge graph for the prediction of rare variant disease associations. While creating dedicated KGs can be time-consuming and inefficient, tools like BioCypher now streamline this process by offering a modular framework that maps database content onto a common ontological space, enabling standardized KG construction.

We have expanded this in the Discussion part of the paper.

3. Use of **negative sampling to utilize class-independent algorithms** such as Bellman-Ford algorithm seems a good idea. It is naturally aligned to the authors claim that this stringent node type-aware negative sampling scheme ensures contrastive learning and improves decision boundary accuracy. We find this approach novel. However, although the authors' claim that they demonstrated the effectiveness of this novel negative sampling strategy, there is a lack of analysis on the impact of negative sampling on performance. The negative samples have a critical role in determining decision boundaries. This should be thoroughly investigated to further confirm the strengths of the approach compared to naive sampling that considers the network homogeneously.

We thank the reviewer for this comment and agree that there was a lack of an in depth-analysis of the impact of different negative sampling strategies on BioPathNet performance. We now conducted the suggested ablation study, systematically assessing the contributions of both node-type-aware negative sampling (NTA) and SANS on all four LP tasks, using five different seeds per task to account for uncertainty. The results are presented in the newly added Figure 2E and detailed further in Supplementary Tables 22-24.
Our findings show that, in nearly all cases, NTA (more so than SANS) improves BioPathNet's performance. While in some cases the improvements are substantial, in others they are modest, there are a few instances where performance remains unaffected.
We observe no improvement in contraindication prediction with either NTA or SANS; unlike drug-disease indication, performance remains unchanged or, in some cases, declines. Since predicting drug contraindications is not the primary focus of drug repurposing task (which prioritizes drug-indication prediction), we did not explore this further but report the findings in Supplementary Table 23.
Overall, we demonstrate the benefit of these design choices.

Minor comments
1. At the time of this paper's submission, TxGNN was available as a preprint on arXiv. Since then, TxGNN has been published in Nature Medicine. We kindly ask the authors to verify whether there have been any significant updates or changes in the final version of the TxGNN model that could impact the comparative evaluation or relevance of BioPathNet's approach. (citation below)

We acknowledge this concern and have addressed it by reviewing all changes in the TxGNN GitHub repository since our comparison. Specifically, we compared the new commit f378c51 to 1000aac (the version used in our study). The key updates include the addition of four more diseases, minimal changes to the disease data split, and a new version of the KG in their paper. Since all methods are benchmarked on the same data, these data-related changes have little impact on performance. Updates to TxGNN's interpretability module also do not affect performance.

However, we noted a modification in how TxGNN computes auxiliary embeddings, which could impact performance and create an unfair comparison. To address this, we re-ran experiments using the latest TxGNN version (as of 20/03/25) on the same data split for adrenal gland, anemia, cell proliferation, cardiovascular, and mental health, repeating each experiment across three seeds.

Results show a slight improvement in TxGNN's performance for drug indication (MRR increased from 0.356 to 0.378), but BioPathNet remains significantly stronger (MRR = 0.513, a 36% advantage). For contraindication, the new TxGNN version showed no notable improvement (MRR = 0.559, similar to the previous version), while BioPathNet achieved 0.532.

Thus, while we acknowledge the TxGNN update and suggest to use its latest version for future comparisons, we believe that our analysis remains valid as presented.

2. The 'adversarial temperature value' defined by the authors needs more clearance. Is it a sampling ratio, or an annealing parameter? In addition, the adversarial temperature for negative sampling was kept constant throughout some of the experiments. Considering that performance can vary depending on the hyperparameters, the authors need to demonstrate on how and why the chosen value is optimal.

The self-adversarial negative sampling was introduced as an optimization trick in RotatE, and has been used in many papers (NBFNet, A*Net, OTE, Ultra). Instead of using 1 positive sample and k uniformly-weighted negative samples, the self-adversarial negative sampling softly redistributes the weights of negative samples based on their losses (some sort of "hardness"), thereby forcing the model to discriminate hard negative samples from positive samples.
We attach here the definition from the RotaTE paper, for the reviewer's inspection

any meaningful information. Therefore, we propose an approach called self-adversarial negative sampling, which samples negative triples according to the current embedding model. Specifically, we sample negative triples from the following distribution:

$$p(h'_j, r, t'_j | \{(h_i, r_i, t_i)\}) = \frac{\exp \alpha f_r(\mathbf{h}'_j, \mathbf{t}'_j)}{\sum_i \exp \alpha f_r(\mathbf{h}'_i, \mathbf{t}'_i)} \quad (5)$$

where α is the temperature of sampling. Moreover, since the sampling procedure may be costly, we treat the above probability as the weight of the negative sample. Therefore, the final negative sampling loss with self-adversarial training takes the following form:

$$L = -\log \sigma(\gamma - d_r(\mathbf{h}, \mathbf{t})) - \sum_{i=1}^{n} p(h'_i, r, t'_i) \log \sigma(d_r(\mathbf{h}'_i, \mathbf{t}'_i) - \gamma) \quad (6)$$

The temperature *alpha* controls the softness of this weight redistribution mechanism, where infinity gives you the original uniform distribution and infinitesimal gives you a one-hot distribution on the hardest negative sample in the batch. We have now clarified this in the Methods section, and added a new paragraph, *Negative sampling* in BioPathNet, in *BioPathNet*

- *Biomedical Knowledge Graph Completion.* There we also refer to the RotatE paper for full definition.

We do not keep the adversarial temperature constant during training across the different tasks, but we tune it together with the other parameters in {0.2, 0.5, 1, 2, 5}, and choose the best value based on the validation performance. As it can be seen in the Supplementary File, the choice of the adversarial temperature parameter is not always the same for each task: for example, in Supplementary Tables 3, 7, 13 and 20 one can notice that this value is task-dependent.

3. If **a low negative sampling ratio is enough to achieve better performance than existing models**, I think this can be considered a strength of the framework. **Our recommendation is to put more emphasis on this point.**

While the number of negatives are lower in BioPathNet compared to TransE/RotatE/DistMult, it is e.g. in the same range for RGCN. Rather, the advantage lies in decreasing the negative sampling space and generating more difficult/appropriate negative samples to improve model learning.

-

Reviewer 2

Link prediction (LP) is a fundamental task in AI for biomedicine. While existing embedding-based methods have shown promising results in predicting potential links in biomedical knowledge graphs (KGs), some limitations remain, such as the lack of interpretability. To address these challenges, the authors proposed BioPathNet, a graph neural network framework to enhance link prediction in KGs. As opposed to the node-embedding frameworks, BioPathNet learns representations of target biological pairs based on relations along the path. Moreover, the authors introduced an external biological regulatory graph (BRG) and a negative sampling strategy to further improve the performance of BioPathNet. To evaluate its performance, BioPathNet is compared with several baselines across four link prediction tasks. Experimental results show that BioPathNet achieves overall better performance than the baselines and also provides interpretable results. While this study shows promising results of the proposed AI method in the biomedical LP tasks, there are still several critical issues that need to be addressed.

Major comments:

1. One of the important technical designs in BioPathNet is the negative sampling strategy (in line 158). However, the authors have not explained the goal of contrastive learning or how it is used, making it difficult to understand the design of the negative sampling strategy.

We thank the reviewer for this comment and apologize that this was not clear in the text. BioPathNet (like the original NBFNet) does not explicitly use a contrastive type of loss function,

which usually is the combination of two terms, one aimed at minimizing the distance between positives, and the other one at maximizing the distance between negatives. The loss function used in BioPathNet for heterogeneous graphs is the one in equation 6 (Method section).
For negative sampling, first of all BioPathNet uses the self-adversarial negative sampling introduced as an optimization trick in RotatE, and has been used in many papers (NBFNet, A*Net, OTE, Ultra). Basically, instead of uniformly sampling *n* negative samples per positive sample, the weights of negative samples are softly redistributed based on the current model embeddings, thereby forcing the model to discriminate based on hard negative samples from positive samples. A parameter called *adversarial temperature* controls the softness of this weight distribution and represents a hyper parameter for BioPathNet.

To further improve the decision boundary and ensure biologically meaningful sampling, BioPathNet employs a *node-type aware (NTA)* negative sampling strategy. Specifically, when selecting negative samples during training, they are drawn exclusively from triplets with the same node type as the corresponding positive example.This approach not only narrows the sampling space but also increases the difficulty of the learning task, encouraging the model to develop a more precise decision boundary with challenging negative samples. Additionally, as discussed in the general response to the reviewers, we explored further improvements to negative sampling beyond NTA by incorporating local graph structure, following the *Structure-Aware Negative Sampling (SANS)* approach suggested by Reviewer 3.
Details on the negative sampling strategy are now provided in the Methods under *Negative Sampling in BioPathNet* and are also explained intuitively, with examples from various LP tasks, in the *Results* section under *BioPathNet accurately predicts links across different biomedical knowledge graphs (KGs)*, specifically in the *Overview of the different link prediction tasks* subsection.

2.

a. Compared with the backbone NBFNet, BioPathNet introduces an external BRG and a negative sampling strategy to improve performance. Introducing an external BRG provides more biological information for the model to learn from, thus leading to better performance. However, **it also creates an unfair comparison for baselines without BRG**, including task-specific baselines and NBFNet.

We appreciate the reviewer’s concern and would like to clarify the role of the Biomedical Relational Graph (BRG) in BioPathNet. The BRG serves as a background knowledge source, incorporating additional entity and relation information solely for message passing, and captures relationships between entities beyond those for which we aim to predict links (the training graph G1), thereby augmenting the foreground G1 graph. Importantly, the edges of the BRG are not used for evaluation or loss computation; they are not the target of prediction but rather enhance learning by providing contextual information (see Methods, where we further clarify this and explain how we modify the NBFNet training algorithm accordingly).

For example, in drug-repurposing, the foreground training graph (G1) is a bipartite graph connecting drugs and diseases, while the BRG (G2) includes additional relationships, such as disease-gene, disease-disease, gene-meta-annotation, and drug-gene connections. Together, G1 and G2 form the full knowledge graph (KG). In BioPathNet, only G1 is used for training (i.e., a subset of the links), while G2 is solely used for message passing. This approach aligns with many biomedical problems. For example, when the goal is to predict gene-function relationships (forming the G1), we do not want to evaluate gene-gene interactions or function-function similarities. However, gene-gene interactions remain valuable in learning, as genes in the same pathway often share functions, and therefore it makes sense to use them in a BRG. Depending on the characteristics of G1 and BRG, G1 alone may suffice for good performance (e.g., in gene function prediction), whereas in other cases, the BRG is essential for achieving strong results.

Among the four tasks used to evaluate the performance of BioPathNet, two tasks — gene-function prediction and lncRNA-gene target prediction — brings Pathway Commons, an external database, as the BRG. The remaining two tasks — drug repurposing and synthetic lethality prediction — use the unused edges (excluding the ones used in supervised training) as the BRG.
Therefore, as it is a key design choice of BioPathNet, together with the negative sampling, we do not believe it creates an unfair comparison to other methods, particularly to NBFNet.

b. **To address this, the authors should conduct experiments with baselines (including NBFNet) that incorporate BRG as well**, similar to the setup in the first task of predicting gene-functional associations.

Related to the concern above, and to fully address the reviewer's concern we benchmark BioPathNet against the original NBFNet, HGT, R-GCN and RAGAT (general link prediction baseline) using the full graph in the evaluation (G1+BRG) for training, following the design choices of each method.

To be completely fair in the comparison, we also leverage our node-type-aware (NTA) negative sampling scheme in the training of R-GCN, RAGAT and HGT (not to have the negative sampling as confounding factor when assessing the contribution of path-embedding architectures, such as BioPathNet compared to node-embedding based heterogeneous GNN architectures). The results are reported in Figure 4A.
Also all task-specific baselines were trained on the full graph (G1+BRG), used according to the specific design choices of each method.
One can observe that the performance of HGT and NBFNet closely matches BioPathNet for gene function and lncRNA target prediction, with NBFNet performing on par with BioPathNet in the synthetic lethality task. However, training NBFNet on large biomedical knowledge graphs proved unfeasible. Training time analysis (Figure 4B) showed that NBFNet's training was significantly slower — up to 9 times longer for lncRNA target prediction and nearly 50 times longer for gene function prediction, where a single seed took over two weeks.

For drug repurposing, which leverages the largest graph, training NBFNet (and RAGAT) was impossible due to memory errors and excessive runtime, even on multiple GPUs. This highlights not only the biological relevance of using the BRG for message passing in BioPathNet — enhancing interpretability and prediction explanations (see Results, *BioPathNet's Interpretability and Human-Curated Evaluation*) — but also its practical advantage. The BRG for message passing enables training path-based methods on large biomedical KGs, such as PrimeKG (>5 million edges), a task that would be prohibitive for NBFNet without this design choice.

For more details about the performance comparison with general link prediction methods please refer to the updated Results section, *Comparison with baselines*. For the newest results of the benchmark against task-specific baselines please refer to Figure 5.

3. In comparison to NBFNet, the current work lacks a systematic study across the link prediction tasks to clearly demonstrate the effectiveness of the BRG and the negative sampling strategy. As a result, the technical differences between BioPathNet and NBFNet remain ambiguous. Ablation studies are recommended.

We thank the reviewer for this comment and completely agree on the importance of demonstrating the impact of BioPathNet design choices on the diverse link prediction tasks examined in this study.

We conducted systematic ablation studies of the BRG across all four tasks, including all disease indication sub-tasks, using five different seeds. The results, shown in Figure 2F, illustrate the percentage change in BioPathNet's performance compared to its BRG-ablated version. Detailed performance values for all tasks, with and without BRG ablation, are provided in Supplementary Table 25. The results are also presented, as an increment (in percentage points) of the basic version of BioPathNet versus the BRG- ablated version in Figure 2F, Results section *BioPathNet's performance and evaluation of design choices*. Using a BRG for message passing on top of the G1 KG improved performance consistently across all tasks, with gains of 1.5–6.3% for lncRNA-mRNA target prediction, 16–24% for gene-function prediction, and up to 84% for synthetic lethality.
In the zero-shot drug-repurposing task — where a 'new' disease is simulated by removing known treatments and similar diseases — removing the BRG severely disrupted connectivity, leaving the new 'diseases' nearly unconnected, preventing meaningful path-based predictions and causing a sharp performance drop. For fairness, we also ran a BRG ablation in a non-zero-shot setting with randomly split data. However, as noted by TxGNN's authors, this setup is too simplistic due to leakage from similar diseases in the training graph (G1), making drug-disease prediction a trivial task and therefore leading to the BRG having no impact on the prediction performance.

We also systematically conducted the ablation study on the node-type-aware negative sampling (NTA) and Structure Aware Negative Sampling (SANS) separately on all four LP tasks, using

five different seeds per task to account for uncertainty (like done for the BRG). The results are presented in Figure 2E and detailed further in Supplementary Tables 21-24.
Our findings show that, in nearly all cases, NTA (more so than SANS) improves BioPathNet's performance. While in some cases the improvements are substantial, in others they are modest, and there are a few instances where performance remains unaffected.
The only case where we did not observe any improvement of either NTA or SANS in contraindication prediction; unlike drug-disease indication, performance remains unchanged or, in some cases, declines. Since predicting drug contraindications is not the primary focus of drug repurposing task (which prioritizes drug-indication prediction), we did not explore this further but report the findings in Supplementary Tables 21-24.
Overall, we demonstrate the benefit of both design choices, use of BRG and negative sampling for performance on biomedical LP with BioPathNet.

As last remark, the benefit of these design choices is not merely reflected on the method performance. For the NTA, while in the original setting and application of NBFNet node type does not matter, in our applications ensures biologically meaningful negative sampling by matching node types. Early experiments also showed that NTA stabilizes performance across hyperparameter variations, simplifying training. Additionally, using BRG for message passing, as in BioPathNet, improves scalability for link prediction on large biomedical knowledge graphs and enables more expressive interpretation rules, as detailed in the manuscript.

4. In the Results part, some of the chosen baselines are somewhat outdated, which weakens the persuasiveness of the comparison results. Despite the promising performance of BioPathNet, the conclusions may differ when incorporating more recent GNN models, such as GIN [1], HGT [2], RAGAT [3], and INDIGO [4].

We definitely agree with the reviewer and updated our benchmarking accordingly, including some of the suggested models.

INDIGO performs inductive knowledge graph completion, making it unsuitable for most of our tasks. GIN, while potentially applicable to graph completion, was originally designed and validated for subgraph classification. Therefore, we focused our comparisons on BioPathNet across all four LP tasks against RAGAT (Relation-Aware Graph Attention Network), HGT (Heterogeneous Graph Transformer), and the original NBFNet, as well as Relational Graph Convolutional Networks (R-GCNs). While R-GCNs were previously tested on the gene function task, we have now extended their evaluation to all tasks.
Results are reported (for MRR and Hits@1-3-10) in Figure 4A and in the Results section *Comparison with baselines.*
Indeed, we observed that these baselines outperform the original RotatE, TransE, and DistMult, with HGT and NBFNet occasionally matching BioPathNet's performance or trailing slightly behind. Meanwhile, R-GCN and RAGAT performed less effectively. However, our benchmarking experiments consistently demonstrate BioPathNet's superior performance on average over

general GNN-based LP methods, with its MRR exceeding all other methods in every case (Figure 4A).

The results of the comparison of BioPathNet against TransE, RotaE and DistMult on gene function prediction have been removed from the current version of the paper.

5. The authors compare task-specific methods for the drug repurposing and synthetic lethality (SL) prediction tasks, but no such comparison is made for the **gene function prediction and lncRNA-target** interaction tasks. **Including task-specific baseline models for the two LP tasks would strengthen the comprehensiveness of the study, making the conclusion more convincing**.

The reviewer is correct, and we have expanded this section to include additional task-specific baselines.

Drug repurposing: Alongside TxGNN, we benchmarked BioPathNet also against DREAMWalk (Bang et al. Nature Communications 2023), a drug repurposing deep learning method using multi-layer random walks. DREAMWalk constructs a fixed biomedical KG including a drug-drug and disease-disease similarity network, along with links connecting drugs and diseases to a PPI network. It generates embeddings via a heterogeneous Skip-gram model and classifies drug-disease associations with XGBoost. Since the original graph (built from ATC and MeSH) was unavailable (the authors only provide a demo graph in their github), we reconstructed it in two ways: (1) **DreamWalk_IC** (Inferred Connections), mapping DrugBank and MONDO (MeSH-inclusive) hierarchies used in PrimeKG to compute drug-drug and disease-disease semantic similarity (with DREAMWalk code), and (2) **DreamWalk_OC** (Original Connections), directly extracting connections from PrimeKG. Both variants used DREAMWalk's default parameters, training on BioPathNet/TxGNN's dataset splits and evaluating on the same test set. DREAMWalk_IC showed better performance on this task (Figure 5C). Results are summarized in Figure 5 B-C and under 'Comparison with baselines', Results section. Details about comparison to DREAMWalk and training are reported in Methods, *Comparison with baselines*, and Extended Methods (supplementary material).

Gene function prediction: to benchmark BioPathNet for this task we selected BIONIC (Bang et al., *Nature Methods* 2022) as a baseline due to its primary application in functional module discovery and gene-function link prediction. BIONIC integrates biological networks using GCNs to learn unified gene embeddings from multiple input networks in either an unsupervised or semi-supervised manner. It processes three input networks — a protein-protein interaction network, a gene co-expression network, and a genetic interaction network — through GCN layers, generating network-specific features that are aggregated via stochastic gene dropout to produce unified embeddings for downstream tasks. Training can optimize network reconstruction (unsupervised) or incorporate functional labels (supervised). Since BIONIC's original implementation only provides yeast networks, we used human networks from the BERTWalk paper (https://github.com/raminass/BERTwalk/tree/main/inputs), which employs a BERT-style masked language model with network propagation regularization for the same task.

BIONIC performed comparably to BERTWalk and outperformed iCell and similar methods in gene function prediction. Its well-documented codebase and flexible deep learning framework further made it our top choice for benchmarking this task. We tested two training scenarios in BIONIC: (1) a semi-supervised approach with gene-KEGG functional triples from BioPathNet's training and validation sets and (2) an unsupervised approach, where we extracted unified gene embeddings and trained an MLP to minimize cross-entropy loss between positives and sampled negatives. The latter performed better and was used for benchmarking.
Although showing good performances for both tasks, BioPathNet surpassed BIONIC on both gene function prediction and lncRNA-target prediction LP tasks. Results are summarized in Figure 5Aand under *Comparison with baselines* Results section. Details about comparison to BIONIC and training are reported in Methods, *Comparison with baselines* and in Extended Methods (supplementary file).

lncRNA-target prediction: Despite the release of several databases collecting RNA-molecule interactions—including lncRNA associations with proteins, DNA, and RNA—systematic methods for predicting lncRNA-target interactions remain scarce. Most approaches focus on RNA-RNA interactions based on biochemical properties or lncRNA-protein interactions. In contrast, BioPathNet addresses a broader question: can we prioritize lncRNA-target interactions (physical, regulatory, direct, and indirect) using link prediction?

After reviewing the literature, we considered three benchmarking tools: **PLPIHS** (Xiao et al., Sci Rep 2017), a deep learning framework leveraging heterogeneous networks of lncRNA-lncRNA similarities, lncRNA-protein associations, and PPIs; and **GAE-LGA** (Gao et al. Brief Bioinform 2022), a recent tool integrating multi-omics data with graph autoencoders to predict lncRNA-PCG associations using LncTarDB2.0 — the same KG as BioPathNet and **DeepLGP** (Zhao et al., Bioinformatics 2020), a general deep learning method for prioritizing lncRNA target genes. DeepLGP, integrates genomic location, expression, and miRNA interactions to construct a gene interaction network. A GCN encoder extracts features, which are combined with original lncRNA and gene features and fed into a CNN classifier to identify true lncRNA-gene pairs. Ideally, we would compare BioPathNet to GAE-LGA, as both use the same dataset. However, our attempts to reproduce GAE-LGA's results on its original data revealed significant training leakage, leading to random MRR and Hits scores. Since fixing these issues was beyond this paper's scope, we excluded GAE-LGA and benchmarked against DeepLGP instead.
DeepLGP was trained on LncRNA2Target v2.0. To ensure fairness, we converted its code to PyTorch, trained on 5 seeds for 30 epochs, and selected the best checkpoint. Since BioPathNet and DeepLGP use different datasets, each was trained separately but evaluated on their shared test set (6 lncRNAs, 19 target genes). Training on the small intersection of their training sets was infeasible due to data limitations.
Given BIONIC's flexibility and the lack of reliable baselines for lncRNA-mRNA target prediction, we also benchmarked BioPathNet against BIONIC for this task (referred to as lncBIONIC). We constructed two input networks for feature embedding: one from the LncTarDB2.0 KG (treating it as a homogeneous network, as BIONIC is not designed for heterogeneous graphs) and another from the PPI section of the Pathway Commons BRG, both used in BioPathNet. lncBIONIC was trained identically to the gene function prediction task.

Results are summarized in Figure 5E and under 'Comparison with baselines', Results section. Details about comparison to DeepLGP and to lncBIONIC and training are reported in Methods, 'Comparison with baselines'.

Finally for the synthetic lethality task, we benchmarked BioPathNet against KR4SL also in inductive setting (see dedicated answer below).

6. Due to the incompleteness of KG data, it's better to show the performance of BioPathNet under the **inductive settings**, particularly since models like KR4SL and NBFNet have demonstrated the performance in the inductive scenarios.

We thank the reviewer for bringing this up. Since BioPathNet is derived from NBFNet, it should support inductive learning and is expected to perform well in this setting. We implemented the inductive setting as an additional dataset and task class in the BioPathNet repository, making the inference on inductive graphs possible. Given that most baselines here are designed for transductive learning, we specifically evaluated BioPathNet's inductive capability on the synthetic lethality task, benchmarking it against KR4SL, which has been shown to outperform transductive methods in this scenario. We trained using the pre-processed KR4SL dataset, splitting SL genes 60:40 into training and test sets. Subgraphs were generated to ensure test genes remained unseen during training. The training set was further divided into training and validation subsets, with no SL gene or BRG node overlap. Validation loss, based on MRR, guided hyperparameter selection, and final performance was evaluated on the test set.

BioPathNet outperforms KR4SL in inductive learning for the SL task on both thresholded and unthresholded data (Figure 5D), demonstrating its effectiveness in this setting.

7. The computational efficiency of models is a critical factor in practical applications. The authors should present relevant efficiency metrics, such as training time.

Yes, we agree that this aspect is crucial, and we appreciate the opportunity to perform this analysis. We now report the training time (in hours) versus performance (MRR) for each method across all four tasks, where applicable, in Figure 4B. The results clearly show that path-based methods, such as NBFNet and BioPathNet, generally achieve higher accuracy than GNN-based methods but require longer training times for larger KGs (e.g., gene function prediction and drug repurposing). This is expected, as path-embedding methods — despite leveraging dynamic programming and the Bellman-Ford algorithm for scalability — must enumerate possible paths and consider broad network neighborhoods. However, among path-based methods, BioPathNet is the most scalable, notably faster than KR4SL for synthetic lethality prediction and significantly faster than NBFNet. In particular, it was not feasible at all to train NBFNet on the drug repurposing task, on the full graph (training graph from G1+BRG). This efficiency is due to BioPathNet's use of the BRG exclusively for message passing rather than loss evaluation, as

explained earlier. We believe this is the key to enabling scalable and practical applications of path-embedding link prediction on large biomedical KGs.

It is also important to note that while BioPathNet could be run on GPUs for all tasks (on an HPC), running NBFNet on training graph from G1+BRG was infeasible on CPUs and required four GPUs. Similarly, RAGAT could only be executed on GPUs across all tasks except for synthetic lethality. As a result, the reported training times may reflect computations on different hardware resources. We discuss these training time results further in the Results section, *Comparison with Baselines*.

8. It is too early to claim that BioPathNet identifies some novel SL pairs, just relying on the evidence from the explanation subgraphs. Wet-lab biological experiments would be needed for that.

We acknowledge that identifying novel SL pairs (or novel interactions in general) requires further validation through wet-lab experiments. The manuscript's focus has now shifted toward the AI methodology, emphasizing the impact and evaluation of different model components across four LP tasks, along with rigorous ablation studies and benchmarking. Consequently, we have toned down our biological claims, highlighting that BioPathNet primarily ranks unknown links in a graph and provides explanations for putative novel relationships. Where possible, we validate these predictions based on existing literature or medical expert opinion, demonstrating their biological or medical relevance. Ultimately, while BioPathNet can propose novel interactions to guide downstream experiments, their validation must come from experimental studies.

9. **Why would the path-based framework be suitable for the 4 LP tasks**? Please explain it from the perspective of data, e.g. distribution of edges in the graphs.

We are not entirely sure if we fully interpret the question correctly, but our explanation is as follows: path embeddings can be seen as a generalization of logical rules. Logical rules are classical methods in the field of link prediction (LP). When LP is performed using logical rules, one typically counts the number of grounded paths (i.e., instances of the left-hand side of a logical rule) that satisfy all the rules learned from the training set. Path embeddings generalize these logical rules in the sense that, instead of relying on explicit rule counting, they represent paths as high-dimensional vectors, and the "counting" process is carried out by neural networks in a nonlinear manner. For all four LP tasks presented in the paper, the learning problem can be formulated as learning logical rules on the specific knowledge graph (KG).

For example, consider a logical rule for gene function prediction (the LP task of assigning genes to functional pathways). As illustrated in Figure 2A, if gene *x* is assigned to pathway *y*, gene *x* interacts with gene *z* in a protein-protein interaction (PPI), and gene *z* phosphorylates gene *w*, then it is likely that gene *w* is also assigned to pathway *y*. This can be expressed as the following logical rule:

function(y,x) ∧ interact(x,z)∧ phosphorylation(z,w) → function(y,w)

In the context of drug repurposing (Figure 2B), consider a scenario where drug *x* is indicated for disease *y*, and gene *z* is involved in both diseases *y* and *w*. In this case, it is likely that drug *x* could also be an indication for disease *w*. The corresponding logical rule can be formulated as:

indication(x,y) ∧ disease(y,z) ∧ disease(w,z) → indication(x,w)

For synthetic lethality (Figure 2C), imagine that gene *x* and gene *z* form a synthetic lethality pair, and that genes *z* and *w* share the same molecular function. This suggests that *x* and *w* are also likely to constitute a synthetic lethality pair. The logical rule in this case can be expressed as:

synth_leth(x,z) ∧ contributes_to(z,y) ∧ contributes_to(w,y) → synth_leth(x,w)

Finally, in the context of lncRNA regulation (where the LP task involves predicting regulatory lncRNA-gene interactions), consider a scenario where lncRNA *x* regulates gene *z*, gene *z* interacts with gene *y*, and gene *y* interacts with gene *w* in a PPI network (Figure 2D). Based on this, it is likely that lncRNA *x* also regulates gene *w*. The corresponding logical rule can be formulated as:

lncRNA_regulation(x,z) ∧ interact(z,y) ∧ interact(y,w) → lncRNA_regulation(x,w)

These examples illustrate how logical rules can model underlying biological relationships, which are implicitly captured by path embeddings in our approach. The training edges distribution is used to learn the model weights and then we apply the model to the test edge distribution to infer new edges based on the 'learned rules'.

We have now clarified this aspect in the Results, *Overview of the different link prediction tasks*, as part of *BioPathNet accurately predicts links across different biomedical KGs* and we hope that this provides an exhaustive explanation for the reviewer, as well as makes it clear why a path-embedding method is highly suitable for these four tasks.

10. To what extend is the performance of the method dependent on the completeness and quality of the KG and BRG? This is important for the practicality of the proposed method.

We acknowledge this concern and agree it is crucial for some tasks. While we cannot determine the completeness of KGs, we computed their sparsity using ∣E∣/∣V∣×(∣V∣−1)|E| / |V| \times (|V| - 1)∣E∣/∣V∣×(∣V∣−1) for directed graphs (the closer this measure is to 1, the higher the connectivity of the graph). Across the four analyzed graphs, this measure ranged from 0.01 to 0.03, yet MRR performance varied significantly across tasks, suggesting density alone does not determine performance. We chose not to include this in the paper, as sparsity is only an indicative measure and does not fully capture graph completeness, which depends on the underlying biological process.

While fully addressing KG and BRG biases is challenging, we analyzed key factors influencing predictions, showing how knowledge sources, graph quality, and structure affect link prediction. As noted in responses to Reviewer 1, biological networks, exhibit study bias, where disease-related proteins have more recorded interactions, overrepresenting known pathways. To assess this, we examined high-degree node bias — a common issue in link prediction — where models favor well-connected nodes, potentially reducing generalization for low-degree predictions.

By computing and visualizing MRR stratified by node degree across all tasks (Figure 3A), we find no correlation between performance and query node degree for three of the four tasks — gene function prediction, synthetic lethality, and lncRNA — suggesting no bias toward genes, lncRNAs, or pathway hubs. However, for drug repurposing, we observe a weak positive correlation (0.23), indicating a tendency of the model to associate drug indications with well-characterized drugs or diseases.

We assessed BioPathNet's performance (MRR) under BRG perturbations, adding or removing 10–80% of edges and deleting the top 5% highest-degree nodes. We also tested whether performance relied on specific relation types or overall connectivity by removing the five most frequent relations per task. Due to computational constraints, we focused on the top five rather than all relations. Results (Figure 3B) show task-dependent effects, with some relations crucial for performance while others are redundant or even detrimental. Notably, removing high-degree nodes significantly reduced performance, as predictive paths often traverse network hubs.

Overall, our analysis underscores that connectivity between training graph from G1 and G2 is more critical for performance than individual relation types, and that bioPathNet is able to find alternative paths, when certain edges are deleted, as far as the connectivity of the graph is not heavily affected, as explained in *BioPathNet's performance and evaluation of design choices*. By conducting the first ablation study on relation types in heterogeneous biomedical KGs, we reveal that certain relations are redundant or even detrimental to prediction tasks. These findings highlight the future need for improved strategies to assess and denoise biomedical knowledge graphs.
The results of this analysis are described in subsection *BioPathNet's performance and evaluation of design choice*', Results section and partly in the paper Discussion.

Minor Comments:
1. The writing can be more succinct and focused. For instance, Subsection 5.1 looks redundant and too general. The title of Subsection 5.5 looks vague and lacks focus. Overall, the manuscript reads a bit verbose. If the authors have used any LLM (like GPT) to generate some of the text, please disclose it.

Thank you for the comment. We have not used ChatGPT for text generation but used ChatGPT and Grammarly for revising and editing syntax and grammar (no use in generating content). We are not sure whether this should be disclosed and how, so we added a sentence to the paper Acknowledgements: ‘we acknowledge the use of chatGPT and Grammarly for text editing’.
Overall, we have now revised the manuscript for conciseness and we hope that this also improves the paper’s clarity.
We have shortened and eliminated redundancy in section 1.5, which we believe is still necessary (to a reader not expert in the field) to set the general problem and introduce the mathematical notation.

2. In Introduction, the authors claim to address the “link prediction in noisy biological knowledge graphs (KGs)” in line 147, but the definition of “noisy” is missing from the context. The authors should clarify this point to avoid confusion.

We have clarified the definition of “noisy” as false positives from experiments, incomplete data and knowledge/experimental bias in the Introduction.

-

3. In line 149, “the node-embedding learning frameworks that optimize the embedding space based on one-hop relations” is confusing. Following [1], GNNs broadly follow a message passing scheme, where each node aggregates feature vectors of its neighbors to compute its new embeddings. After k iterations of aggregation, the final node embeddings capture the structural information within the node’s k-hop neighborhood, not one-hop.

We apologize for not being precise there, of course we agree with your statement. While the embedding space is optimized based on one-hop relations in the early KGE methods (RotatE, DistMult, TransE), this is no longer the case for RGCN and other GNN approaches. We have thus adapted the text correspondingly in the Introduction.

4. Providing an overview diagram of the model could enhance the reader’s understanding of BioPathNet's architecture.

We added a new figure, Figure 7, to the Method section of the paper to explain the architecture and message passing/aggregation scheme in detail, we thank the reviewers for the suggestion.

-

5. In line 97, the juxtaposition of “knowledge graphs (KGs)” and “heterogeneous multi-relational networks” seems odd, since KGs are a specific case of heterogeneous multi-relational networks.

We have changed the sentence to 'Recent efforts use heterogeneous multi-relational networks, more specifically knowledge graphs (KGs)'

6. There are some typos in Supplementary Table 7. For instance, the "AUPRC of 0.605" is labeled in bold but is actually the second-best result. Similar typos can be found in the "AUPRC 0.57".

We thank the reviewer for pointing this out, we corrected this.

7. The icons of the models are hard to distinguish in Supplementary Figure 3C.

We have now increased fonts and symbols everywhere in Supplementary ex Figure 3 (now Supplementary Figure 4).

Reviewer 3

The paper introduces BioPathNet, a novel graph neural network framework designed to enhance link prediction in biomedical knowledge graphs through path representation learning. The authors leverage the Neural Bellman-Ford Network (NBFNet) to combine path-based reasoning with a Background Regulatory Graph (BRG), resulting in improved accuracy and interpretability. BioPathNet was validated across several tasks such as gene function annotation, drug repurposing, and synthetic lethality prediction, demonstrating strong performance compared to state-of-the-art methods. Despite its promising contributions, several methodological concerns and areas for improvement should be addressed.

In general, the paper is difficult to read and understand. Part of this is not the fault of the authors because Results before Methods is confusing. The methods do not describe where BRG comes from and how it works and so this is a total confusing element of the paper (there are a few sentences in 5.3 and as far as I can tell, nothing else).

We sincerely apologize for any confusion. The Background Relational Graph (BRG) serves as a supplementary knowledge source, incorporating additional entity and relationship information exclusively for message passing. It enhances the foreground training graph (G1) by capturing relationships beyond those being predicted, enriching the learning process without being used for evaluation or loss computation (as explained a bit more in detail in Methods now). The BRG's edges are not the target of prediction but provide crucial contextual information.

For instance, in drug repurposing, the foreground graph (G1) is a bipartite drug-disease network, while the BRG (G2) includes disease-gene, disease-disease, gene-meta-annotation, and drug-gene relationships from the rest of PrimeKG. Together, G1 and G2 form the full knowledge graph (KG). In BioPathNet, only G1 is used for training and message passing,

while G2 only supports message passing. This makes sense for other biomedical applications. For example, when predicting gene-function relationships (G1), gene-gene interactions or disease-disease interactions and gene-chemical interactions (G2) are not evaluated but still aid learning, as, for example, genes in the same pathway often share functions. Depending on the task, G1 alone may yield already good performance (e.g., gene function prediction), although the used of the BRG clearly improves this performance (Figure 2F, Supplementary Table 25) whereas in others (e.g., drug repurposing), the BRG is crucial and without its use the task performance is really compromised.

Among the four tasks in BioPathNet's evaluation, gene-function and lncRNA-target prediction use Pathway Commons as the BRG, while drug repurposing and synthetic lethality prediction rely on unused edges within their respective KGs. In drug repurposing, the BRG consists of all PrimeKG edges except drug-disease indication/contraindication links. For synthetic lethality, the BRG includes gene-GO term/pathway connections and additional synthetic lethality gene-gene interactions distinct from those in G1. We have now clarified this in detail in the Results section (Figure 1), under *BioPathNet accurately predicts links across different biomedical Knowledge Graphs (KGs)*, paragraph *Overview of the different link prediction tasks*, and expanded the Methods subsection I*ncorporation of a Background Relational Graph (BRG)* within *BioPathNet - Biomedical Knowledge Graph Completion.*

We have also demonstrated that incorporating an external BRG or a subset of a large KG for message passing not only impacts model performance but also:

1. **Enables scalability** – Unlike NBFNet and other link prediction methods (e.g., RAGAT, NBFNet) that require training on the full graph, BioPathNet's design allows learning on large biomedical KGs. This makes tasks such as drug repurposing and gene function prediction — infeasible due to computational constraints for e.g. NBFNet — achievable.

2. **Enhances biological interpretability** – The BRG provides richer contextual information, improving the explainability of predictions, as discussed in the subsection *"BioPathNet's Interpretability and Human-Curated Evaluation."*

Also, it is not clear whether the primary contributions here are the new link-prediction method or the applications. **So the paper kind of goes back and forth featuring the link-prediction vs. focusing on the application results. The application results are all over the place and so hard to digest the biological strengths of the method.**

The primary goal of the paper is to enable path-embedding learning for link prediction on biomedical knowledge graphs by introducing the BioPathNet method. We state clearly in the Results section *BioPathNet: Path embedding as alternative to node embedding for graph completion on biomedical KGs* that we do not provide an entirely novel method for link prediction, but we build on NBFNet, originally not designed for biomedical task, but tested on other kind of heterogeneous graphs, such as FB15k-237, an LP dataset from the Freebase knowledge graph, and WN18RR , a dataset derived from WordNet. Extending NBFNet (which is not applicable out-of-the box to many of the LP tasks presented here), BioPathNet introduces crucial design choices that, not only enable scalable applicability of the method to biomedical KGs, but also take into account the specifics of knowledge graphs (such as different node types, which need to be taken into account during training), different types of relation or relevant contextual information that can be leveraged for specific link prediction tasks, and by that improves prediction performance and biological interpretability over its NBFNet baseline.

Building on NBFNet—which is not directly applicable out-of-the-box to many LP tasks presented here—BioPathNet introduces key design choices that enable scalable application to biomedical KGs. It accounts for critical biomedical KG-specific features, such as node types (essential for training), diverse relation types, and relevant contextual information tailored to specific link prediction tasks. These enhancements improve both prediction performance (as demonstrated in Figure 4) and biological interpretability over the NBFNet baseline. We have made clear now in Intro, Results and Discussion that this is the main contribution of BioPathNet.

Following the reviewer's recommendation, we have significantly revised the paper's writing and structure. In the initial sections of the results, we now focus on the methodology, highlighting the differences between node embedding and path embedding (Figure 1), as well as key design choices unique to BioPathNet — such as negative sampling schemes and the use of a BRG (now also illustrated in Figure 1C-G) — compared to prior baselines. We provide evaluation of BioPathNet on different graphs, corresponding to different LP tasks in biomedicine (Figure 2A-D, Table 1). We provide comprehensive ablation studies to demonstrate the impact of these design choices (Figure 2E-F), conduct extensive perturbation experiments to evaluate the effect of noise and BRG edges on BioPathNet's performance while identifying potential biases (Figure 3), and offer a more thorough benchmarking analysis against both general LP (Figure 4A) and task-specific baselines (Figure 5), complemented by a runtime analysis for all baselines (Figure 4B).

Importantly, we have separated the methodological assessment from the application results. The biological case studies, previously interwoven with methodological discussions, are now placed at the end of the results section (Figure 5). Their descriptions have been streamlined, with speculative aspects minimized and we utilize them to

demonstrate BioPathNet's interpretability — showing how retrieved explanations provide biological context for predictions. Even though they represent only a few examples, they are crucial for demonstrating that the predictions are biologically meaningful.

We hope these changes help streamline the manuscript's main message, enhance clarity and coherence, and effectively highlight the method's effectiveness.

The general message seems to be "it can do everything" but there is not deep evaluation of this implicit claim, **but instead a few examples from different areas of biomedicine which are interesting but don't give confidence about overall performance**.

We do not claim that BioPathNet is a universal solution for all link prediction tasks, and we apologize if our initial presentation gave that impression. We recognize the importance of thoroughly supporting its capabilities across different tasks with rigorous experimentation. BioPathNet is though a general framework designed to be applicable to various biomedical link prediction tasks. It is not specific to drug repurposing or any single application but is, in theory, adaptable to tasks that meet the following criteria (although its application in other tasks remains to be demonstrated):

1. The link prediction problem can be framed as learning meaningful biological rules or paths in large biomedical knowledge graphs.
2. The type of biological nodes is crucial for training.
3. The task benefits from contextual information, which can be formalized as a Background Regulatory Graph (BRG).

We view this generalizability as a key strength. To demonstrate this, we tested BioPathNet across different networks (as done in NBFNet, which was evaluated on multiple web-based datasets) to address distinct link prediction problems. We specifically chose tasks that reflect significant biomedical challenges at the moment in the community while also illustrating how BioPathNet's design choices impact performance. For this reason, we retained all four case studies — gene function prediction, drug repurposing, synthetic lethality, and lncRNA-target prediction — in the manuscript.

We acknowledge the reviewer's request for more extensive evaluation and benchmarking, which we have now addressed through rigorous ablation studies, perturbation analyses, and comprehensive benchmarking experiments. While BioPathNet demonstrates strong performance in certain tasks (e.g., drug-disease indication prediction and gene function annotation), there is room for improvement in others. For instance, more accurate knowledge graphs could enhance performance, as demonstrated by our ability to improve results through denoising original connections. In the case of lncRNA-target prediction, a more reliable training set is critical, and tailoring BioPathNet specifically for this task in

future work could yield a dedicated, improved method. These points are now discussed in detail in the manuscript's Discussion section.

It may be that the authors should present one paper with the computer science method of link prediction, and then one **or more application papers focusing on specific tasks and validating the performance against other methods. As it is, there is (for me) too much.**

We acknowledge the reviewer's concern. Building on our response above, we believe the revised manuscript now focuses primarily on methodology rather than specific biological applications. The four knowledge graphs and tasks serve to demonstrate BioPathNet's general applicability across different datasets — an essential point in supporting our claim of generalizability to various biomedical link prediction tasks. However, the focus is not on the individual applications or their specific benefits, which might be the topic of future manuscripts.

While we have expanded the benchmarking of each task against task-specific baselines, we acknowledge that further work is needed to refine each application, conduct more in-depth evaluations, and validate performance on independent, orthogonal datasets. We have made this clear in the manuscript's Discussion section.

Major comments:

1. Node-type Aware Negative Sampling: The use of node-type aware negative sampling is a step forward, but it could introduce bias by over-representing certain node types and neglecting others, especially in imbalanced biological datasets. This might lead to suboptimal performance on rare or underrepresented node pairs, requiring more careful handling of data imbalance across various types of nodes. We suggest implementing methods that can dynamically adjusts the selection of negative samples based on graph density. This would help balance the distribution of node types and ensure that the model does not overfit to certain dense regions while ignoring sparser sections of the graph.

We acknowledge the reviewer's concern that node-type-aware negative sampling (NTA) may disproportionately sample overrepresented types. For instance, in drug-disease indication prediction, diseases that are overrepresented in the dataset are sampled more frequently, whereas less common diseases may have fewer negative samples, potentially affecting performance across different disease splits. However, a purely uniform or node-type-agnostic negative sampling strategy is also biologically unrealistic — sampling drug-gene interactions as negatives when the goal is to predict drug-disease interactions would not make sense.

We fully agree that incorporating local graph structure into negative sampling could further improve the method. We appreciate the reviewer's suggestion, which led us to implement a structure-aware negative sampling (SANS) approach based on Ahrabian et al. (https://arxiv.org/pdf/2009.11355). Specifically, we reimplemented their algorithm, which leverages the knowledge graph (KG) structure to generate negative samples by restricting them to the k-hop neighborhood of the head node. Additionally, we used random walks to approximate a node's local neighborhood. The rationale behind this method is that sampling negatives from regions with similar density (or similar graph properties) makes the contrastive learning task more challenging. Nodes within the same neighborhood — but without a direct connection — are more likely to be functionally related than distant nodes. This approach should also mitigate biases introduced by uniform sampling, where negatives from denser graph regions are selected more often, potentially leading to poorer generalization in sparse areas.

We have introduced this concept in Figure 1G (*BioPathNet: Path Embedding as an Alternative to Node Embedding for Graph Completion on Biomedical KGs*) and detailed it in Methods (*Negative Sampling in BioPathNet*). To assess its impact, we conducted an ablation study on NTA and SANS across all four link prediction tasks, using five random seeds per task for robustness, as in the BRG analysis. Results (Figure 2E, Supplementary Tables 21-24) show that NTA generally improves BioPathNet's performance, with the best results often achieved by combining NTA and SANS. While improvements were substantial in some cases and modest in others, performance remained unchanged in a few instances. In drug repurposing, SANS's contribution varied across disease splits, sometimes enhancing and sometimes hindering performance.

The only exception was contraindication prediction, where neither NTA nor SANS provided improvements, and in some cases, performance even declined. Since predicting drug contraindications is not the primary objective of the drug repurposing task (which focuses on drug indication prediction), we did not explore this further but have reported the findings in Supplementary Tables 21-24.

While we acknowledge in the manuscript that SANS, when combined with NTA, improves performance for some tasks—and we provide our implementation in the BioPathNet repository—we conducted perturbation experiments and benchmarks using BioPathNet with NTA alone (as stated in the Rebuttal letter). This decision was based on two key reasons: (1) SANS, due to its random walk-based negative sampling, is significantly more computationally expensive than NTA (even with a walk length of 2, see Methods); and (2) to ensure consistency, we applied the same NTA sampling procedure across all experiments and baselines (see Methods, *Comparison with Baselines*).

2.

a. BRG and Data Leakage: How the BRG is incorporated into the model should be more clearly described. There is limited discussion of the role of the BRG in training and inference and so the reader is left uncertain about the impact these edges have on the prediction made. The paper would benefit from a more detailed explanation of how the BRG is incorporated during both the training and prediction phases. It remains unclear whether the BRG is used consistently or differently at these stages, which could have implications for the model's generalization and performance.

We hope the response above clarifies the role and usage of the BRG in training across all tasks.

We have now also explicitly clarified in the *Methods* section, *Incorporation of a Background Regulatory Graph*, that the same BRG used for message passing during training — intended to learn the distribution of rules, important edges, relations, and paths relevant to the task — is also used during inference in the transductive setting. By definition, transductive learning implies that the training and inference graphs are identical, meaning (novel) link predictions are made on nodes that were already present in the training graph.

In contrast, for the inductive setting, e.g. in synthetic lethality predictions (see *Results* section, *BioPathNet versus task-specific baselines* in *Comparison with baselines*), the BRG used during inference is by definition different from the one used for training. Specifically, in this task part of the original BRG is used for training, while another portion is reserved for inference. In this case, the inference graph contains nodes that were not present in the training graph.

Importantly, the model's loss and final performance are not evaluated on the edges of the BRG but only on the edges of G1 that were left out to form the test set — these edges were never seen during training (this is also now clarified in Methods, *BioPathNet training and LP*). As a result, there is no data leakage, and generalization is preserved.

b. It is not clearly stated if the **edges present in the BRG (G2) overlap with the predicted edges**. This could artificially inflate performance metrics, and it is important to ensure that the model does not predict relationships that are already present in G2 during training. **Furthermore, the assumptions about what type of network is used as the BRG are not clear.** If the BRG includes noisy or missing data, as is common in biological networks, techniques such as graph regularisation or uncertainty estimation should be considered.

Edges in the BRG (G2) never overlap with the predicted edges, as predictions are made on entities in G1, and the BRG contains different relation types than those in G1. For example,

when predicting gene-function relationships, the BRG includes gene-gene interactions and gene-chemical associations, which are not part of the prediction task. This holds for the gene function, drug repurposing, and lncRNA-target prediction datasets.

However, in the synthetic lethality task, G2 includes not only gene-biological function/pathway relationships but also additional SL gene-gene interactions. As a result, the model may predict an SL relationship that already exists in the BRG, potentially inflating performance evaluation. To prevent this, we carefully filter out any predicted SL pairs that are present in the BRG when evaluating on the test set, as detailed in the Methods section under *Model Evaluation*.

Yes, we implement regularization in BioPathNet (as in the original NBFNet) by incorporating normalization layers (LayerNorm) in the architecture, which standardizes node embeddings across features to stabilize training, improve generalization, and prevent overfitting. Ultimately, it acts as a regularizer by reducing sensitivity to small variations in node features, helping prevent overfitting. This clarification has now been added to the methods section (new Figure 7: Model Architecture, paragraph *Neural Formulation* in Methods). NBFNet also includes another regularization technique — dropping one-hop edges during training to encourage learning longer paths and reduce overfitting. However, in our early BioPathNet experiments, this had no effect on test performance, so we did not use it in the current setup. Nonetheless, given BioPathNet's adaptability to multiple biomedical KG tasks, we retain this option in the code for potential future applications.

-

We believe that the incompleteness and noise in biological networks or knowledge graphs introduce uncertainty into predictions during evaluation. To address this, we account for uncertainty at multiple stages of our experiments by using different random seeds (for all analyzed tasks), varying disease splits for drug repurposing and SL edge confidence score thresholds for synthetic lethality tasks, and sampling different negative examples at each epoch.

3. Baseline Selection Discussion: The selection of baseline models, while including common approaches, lacks thorough justification. The baselines chosen address generalized link-prediction tasks, but do not specifically provide points of comparison for link prediction in biological networks. A more in-depth discussion of why certain baselines were chosen, and what novel aspect of the proposed model creates the increased performance would be helpful.

We compared BioPathNet to general link prediction baselines for three main reasons: (1) BioPathNet is not task-specific and can handle diverse biomedical LP tasks, making it fair to compare against general-purpose models; and (2) unlike most existing KGE-based methods that rely on node embeddings, BioPathNet uses path embeddings, and we aimed

to evaluate the advantages of this approach, or more generally if design choices in the algorithm and architecture is making a difference for these kind of task.
Initially, we benchmarked BioPathNet against simpler KGE models (RotatE, TransE, and DistMult) to assess the benefits of path embeddings over node embeddings, particularly for gene function prediction. However, reviewers highlighted the need to compare against more advanced models that incorporate larger neighborhoods and graph heterogeneity.

In response, we moved the simpler KGE results to the supplementary material and expanded our baseline comparisons. We retained R-GCN, as the foundation of TxGNN, therefore already leveraged for biomedical link prediction) and included three additional state-of-the-art baselines in LP tasks — RAGAT (Relation-Aware Graph Attention Network), HGT (Heterogeneous Graph transformer), and original NBFNet — evaluating them across all four tasks.

We agree with the reviewer that in contrast, task-specific baselines are optimized for a particular task, potentially giving them an advantage on that task over more generalized models and we apologize that we did not provide in the first place a comparison to task-specific baselines.

As suggested by reviewers 2 and 3, we have incorporated several task-specific baselines:
DREAMWalk (optimized in two settings) and TxGNN were chosen for drug-disease prediction, as both have shown strong performance in drug repurposing despite their differing underlying models.

BIONIC (optimized in two settings) was chosen to benchmark BioPathNet for function prediction due to its proven ability to generate gene embeddings from multiple networks and its strong performance in gene function assignment. It matches or outperforms newer methods in semi-supervised settings, performing comparably to BERTWalk—a BERT-style masked language model for gene function prediction—and surpassing iCell and similar approaches. Its well-documented codebase and flexible deep-learning framework further supported its selection. Given the scarcity of robust lncRNA-gene pair prediction models, we also adapted BIONIC as an additional baseline for this task.

KR4SL (evaluated in both transductive and inductive settings, as requested by Reviewer 2) was selected to benchmark BioPathNet on the synthetic lethality task due to its path-embedding approach and superior performance over previous methods in predicting SL pairs. We further extended this benchmark to the transductive setting (see Answer 6 of Major comments to Reviewer 2).

DeepLGP was selected as an additional baseline to benchmark BioPathNet for lncRNA-target prediction. While few deep learning methods exist for this task, some lack

publicly available code, and one exhibited severe data leakage during training, rendering its evaluation unreliable (results omitted).
Adapting these models required a significant coding effort to align them with our framework or vice versa. Baseline selection was overall based on relevance to biomedical link prediction tasks, demonstrated superior performance, usability, and well-documented code/data for reproducibility (arguably the most critical criterion).
We justify these choices in the results section *Comparison with Baselines* and provide a more detailed response in our reply to **reviewer 2, point 5 of Major comments**. Methodological details can be found in *Comparison with Baselines* (Methods section) and Supplementary File, paragraph *Baseline Methods used for comparison*. Benchmarking results are reported in Figures 4 (task-agnostic baselines) and 5 (task-specific baselines).

Overall, we emphasize in the Discussion and Conclusion that we believe that BioPathNet's performance is driven by its backbone model — path embedding in a neural formulation — and key design choices, namely the BRG and negative sampling, which make it both effective and feasible for biomedical KGs.

REBUTTAL LETTER

We sincerely thank the reviewers and editor for their time and effort in evaluating our manuscript. We appreciate the opportunity to proceed with publication pending minor revisions, and have carefully addressed all remaining comments point by point below, and in their respective place in the manuscript. Changes are highlighted in red in the manuscript. Besides changes in the text, to address the remaining concerns from the reviewers we have added three more supplementary figures (see details below):

- **Supplementary Figure 6**, provides a global summary of the most important nodes and node types within the top explanation paths for both the drug repurposing and synthetic lethality tasks. This directly addresses the reviewer's concern regarding the use of anecdotal evidence by offering a more comprehensive view beyond individual examples.
- **Supplementary Figure 7**, further addresses the issue of anecdotal evidence, but from a quantitative perspective. It presents a biological and medical term enrichment analysis of the gene nodes found in the top explanation paths for drug repurposing and synthetic lethality. This allows for a more systematic comparison with established biological pathway and disease gene databases.
- **Supplementary Figure 8**, responds to the reviewer's comment regarding the variability in drug-indication prediction performance across different disease splits. It provides an explanation, which then aids in the discussion part of the manuscript, of this observed variability.

Reviewer 3

1. The authors emphasize that BioPathNet is more interpretable than standard node-embedding models because it reasons over paths and allows visualization of which multi-hop paths contribute to a prediction. While this is promising, the evidence is anecdotal — they include a few examples of predicted paths and claim biological plausibility, but there is no quantitative measure of interpretability, such as how often the top-ranked paths align with known biological pathways or mechanisms.

The paper provides examples of interpretability, each illustrating how BioPathNet's top-ranked paths for a given triplet can be naturally interpreted, to highlight the tool's practical utility: for a specific prediction made, users are interested in the underlying molecular mechanisms or explanations that led to that particular example. These interpretations are done on a per-triplet basis. While such analysis could, in principle, be extended to all triplets, systematic evaluation is challenging. Verifying alignment with known biology is limited by the variability across knowledge graphs—each with distinct entity types and relationships—and differs significantly from resources like GO or other

ontologies. A systematic comparison across graphs would require specialized tools, which lies beyond the scope of this work.

We acknowledge that it is important to get a more general and/or quantitative understanding about the plausibility of the explanations, beyond few examples, and to address the reviewer concern here we did the following:

First, we wanted to get a global view of the important nodes in paths driving the top predictions for the different link prediction tasks, beyond a few examples.

For each drug- indication prediction task (e.g., adrenal gland), we selected the top 50 predictions from the test set that were consistently recovered across most of the five random seeds—ensuring robust predictions before extracting explanations. For each such triplet, we extracted the top 10 most influential explanation paths. We then summarized the most frequent node types occurring across these paths in a bar plot (new **Supplementary Figure 6**), and, within each type, highlighted the five most frequently traversed nodes. This analysis was performed for all five disease-specific indication tasks and for synthetic lethality prediction. Due to the broader functional diversity of genes and lncRNAs involved in general cellular processes (compared to specific disease area in the drug repurposing task or cancer), we did not conduct this aggregation for those tasks, where such summaries would be less informative.

This analysis is quantitative in terms of assessing the importance of nodes and node types traversed by the top explanation paths, while also allowing a qualitative evaluation of whether the identified nodes are biologically meaningful for the task. Unlike anecdotal insights based on a few examples, it provides a broader overview of important features at a global level for the task at hand.

Our results elucidate that e.g. *existing drugs* and *disease terms* are the node types that most explain disease-drug indication, followed by connections with *genes* and *phenotypes*. The most frequent nodes along the explanation paths align well with the task predictions. For instance, in the cell proliferation disease split, the top disease-related nodes all pertain to cancer subtypes or processes such as somatic mutations and uncontrolled cell growth. Similarly, the top drug nodes—such as Tetramin, Rucaparib, and Altretamine—are chemotherapeutic, antineoplastic, or cancer maintenance treatments, demonstrating that the model learns plausible new indications by repurposing drugs already used in oncology.

In line with perturbation experiments assessing the impact of different graph elements on BioPathNet's performance for synthetic lethality prediction, gene–gene interactions—and thus the node type *gene*—emerge as the most important contributors to explaining predicted lethal pairs. This is followed by substantially lower contributions

from *cellular component (CC)*, *molecular function (MF)*, *biological processes (BP)*, and *pathway*. Nonetheless, the specific nodes frequently traversed in top explanation paths are highly relevant in the cancer context. For example, proto-oncogenes KRAS, NRAS, and HRAS—members of the RAS family and among the most commonly mutated genes in cancer—are among the most influential. Other key nodes include the plasma membrane compartment, critical for maintaining cancer cell homeostasis and often associated with overexpressed proteins involved in growth responses, as well as signaling pathways such as the RAS/BRAF cascade, which play a major role in carcinogenesis.

Similarly consistent results are observed across the other disease splits, as detailed in the Results section under *"BioPathNet's interpretability and human-curated evaluation"*, specifically in a new paragraph titled *"Global explanation analysis"*.

To enable a more systematic comparison of our explanations with established pathways and biomedical knowledge bases, we also conducted an over-representation analysis on the most important genes—defined as those most frequently appearing nodes in the top 10 explanation paths for the top 50 predicted links (as previously described). Specifically, we analyzed DisGeNET terms for four disease splits from the drug repurposing task (DisGeNET being a comprehensive knowledge base that integrates information on human diseases, symptoms, and associated molecular mechanisms from the literature), and KEGG pathways for the synthetic lethality and cell proliferation tasks, to allow for a more detailed comparison with known cancer-related pathways.

The results are presented in the new **Supplementary Figure 7**, which highlights the top 10 enriched terms as well as the least enriched terms (used as a control). These findings reveal a strong concordance between the nodes in our explanation paths and established biomedical knowledge—for example, a notable overlap with known KEGG cancer pathways in the context of synthetic lethality and cell proliferation. The DisGeNET enrichment analysis further reinforces the biological relevance of our explanations, showing that the genes involved in the top explanation paths are significantly enriched for terms directly related to the disease area for which predictions are made. For instance, in the cardiovascular disease split, the top enriched terms include *Hypertensive Disease*, *Atherosclerosis*, *Coronary Heart Disease*, *Myocardial Infarction*, *Cardiovascular Diseases*, *Coronary Artery Disease*, and *Hypertension*, supporting the plausibility of the extracted explanations. Similarly striking patterns are observed for the mental health, adrenal gland, anemia, and cell proliferation splits, further demonstrating the alignment between our model's explanations and established biomedical knowledge.

We believe the analysis now extends well beyond a few anecdotal—though still informative—examples, offering a more comprehensive and systematic evaluation.

2. In the drug repurposing task, BioPathNet is evaluated across multiple disease areas in a zero-shot setting. However, performance (e.g., MRR) varies dramatically. For adrenal gland or cell proliferation, performance is strong (MRR > 0.6–0.8). For mental health and cardiovascular, MRR is much lower (~0.23–0.36). The paper does not discuss possible reasons for this inconsistency.

It is indeed true that performance on the drug indication task varies significantly across different disease splits. Similar trends in Recall@100 were observed in the original TxGNN paper, where the adrenal gland and *cell proliferation* splits showed the highest performance, anemia performed moderately, and mental health and cardiovascular had the lowest scores. The fact that different models reproduce this performance pattern suggests that the variability is more likely driven by intrinsic characteristics of the disease splits—the complexity of the disease and the richness of available data (e.g., known drugs or molecular mechanisms represented in the graph)—rather than by the specific modeling approach.

We explored this further and made a striking observation: there is a strong Pearson Correlation ($r = 0.81$) between the performance of each drug repurposing task—measured by mean reciprocal rank (MRR)—and the average connectivity of drugs in the training set within the G1 graph for each disease. This suggests that predictive performance is largely influenced by how well sub-diseases in a certain split are connected to drugs known to treat other conditions, rather than by other graph properties. These findings are summarized in Supplementary Figure 8 and further discussed in the *Discussion* section of the paper.

3. SynLethDB-v2.0 includes experimental and predicted SL pairs, but the authors did not clearly state which type of edges were included. Were predicted edges used in the training set? How was label noise from the predicted pairs handled?

We acknowledge that the synthetic lethality (SL) dataset from SynLethDB-v2.0 may contain noise or false positives, as it aggregates evidence from multiple sources. Each SL pair in SynLethDB-v2.0 is assigned a confidence score reflecting the level of support for the interaction. This issue is explicitly addressed in our manuscript. Unlike the original KR4SL paper, which trains on the entire SL dataset regardless of confidence scores or the nature of the evidence (experimental vs. computational), we conduct

training and downstream analysis using two datasets (see *Methods: Synthetic lethality (SL) prediction task - Identifying novel SL gene pairs*): (i) the full dataset ("unthresholded data") for direct comparison with KR4SL, and (ii) a filtered version ("thresholded data") that includes only SL pairs with a confidence score above 0.3 for training, validation, and testing.

As shown in Supplementary Figure 3A, this threshold effectively removes all SL pairs supported solely by computational predictions. Thus, potential noise from low-confidence pairs has already been addressed. Furthermore, Supplementary Figure 3B reports performance comparisons between BioPathNet and KR4SL across varying confidence thresholds, highlighting how prediction accuracy changes with data quality. This approach ensures a fair and robust evaluation. Please also note that in the relevant *Methods* section (*Synthetic lethality (SL) prediction task - Identifying novel SL gene pairs)* we write:

‘In transductive setting, BioPathNet was trained and evaluated on SynLethDB SL pairs across confidence thresholds from 0.1 to 0.8. For final training, pairs below 0.3 were excluded, removing most, if not all, computationally predicted interactions (Supplementary Figure 3A). The final dataset included 8,770 training, 3,172 validation, 6,254 test, and 13,161 known SL pairs in the BRG (Supplementary Table 10). The filtered set is referred to as 'thresholded data,' while the original is 'unthresholded data', which includes all SL pairs from SynLethDB-v2.0 without filtering by confidence score. ‘

Minor Concerns

1. The number of negatives per positive and how this ratio is selected or tuned is not clearly summarized in the main text or methods.

The ratio of positive to negative samples during training is a hyperparameter in our model and it is optimized during training, based on the MRR on the validation set. The optimal values for each task are reported in the corresponding supplementary tables: Supplementary Table 3 (gene function prediction), Supplementary Table 7 (drug repurposing), Supplementary Table 13 (synthetic lethality), and Supplementary Table 16 (lncRNA-target prediction).

This hyperparameter was tuned within a range informed by the original NBFNet implementation—typically 32 or 64 negatives per positive. Details of this optimization process are provided in the Extended Methods section of the Supplementary Material. For most tasks, a 1:64 ratio yielded the best results, with 1:32 optimal in a few cases. In the inductive setting of the synthetic lethality task, performance improved with a higher

number of negatives, so we extended the search range to include 1:128 (see *Section 1.4: Synthetic lethality gene pair prediction task*).

This hyperparameter, among others, was also tuned for the baseline methods compared to BioPathNet, as mentioned in the Method's subsection *Comparison with baselines*: 'All baseline methods were re-trained using optimal parameters—either selected based on validation MRR when possible or set to the default values recommended by the original authors—to ensure a fair comparison', and their optimal settings are reported in *Section 1.6: Baseline methods used for comparison* (Extended Methods, Supplementary Material).

While each supplementary table is referenced in the Methods section discussing BioPathNet training, we now explicitly clarify that the number of negatives per positive is a hyperparameter optimized during training (see *Methods: BioPathNet training on four different biomedical tasks)* and refer to Extended Methods and supplementary tables for more details.

2. The value of k used for k-hop neighborhood in structure-aware sampling is mentioned only indirectly in the rebuttal but is not explicitly reported in the manuscript.

We thank the reviewer for this comment. Now we report the value of k=2 for the SANS negative sampling, specified in the paragraph *Negative Sampling in BioPathNet, Methods* section.
